# Symmetry-guided fusion converts weak liver pyruvate kinase inhibitors into potent allosteric activators

**DOI:** 10.1039/d6md00604c

**Published:** 2026-09-24

**Authors:** Leticia Monjas, Alexandra Rodriguez Rivero, Anders Foller Füchtbauer, Oscar Nilsson, Liliana Håversen, Aleksei Lulla, Paul Brear, Mikael Rutberg, Per-Olof Bergh, Jan Borén, Adil Mardinoglu, Marko Hyvönen, Morten Grøtli

**Affiliations:** a Department of Chemistry and Molecular Biology, University of Gothenburg Medicinaregatan 7b SE-413 90 Gothenburg Sweden grotli@chem.gu.se; b Department of Molecular and Clinical Medicine, University of Gothenburg and Sahlgrenska University Hospital Bruna Stråket 16 SE-413 45 Gothenburg Sweden; c Department of Biochemistry, University of Cambridge 80 Tennis Court Road Cambridge CB2 1GA UK; d Science for Life Laboratory, KTH - Royal Institute of Technology Tomtebodavägen 23 SE-171 65 Stockholm Sweden

## Abstract

Structure-guided exploitation of symmetry within multimeric protein interfaces offers an opportunity to convert partial ligand-binding poses into higher-affinity modulators. Here, we report a class of symmetrical “Z-shaped” allosteric activators of human liver pyruvate kinase (PKL), generated by symmetry-guided fusion of binding poses identified in inhibitor-bound crystal structures. The resulting ligands span the inter-subunit pocket at the tetrameric dimer–dimer interface and display substantially enhanced activity, with several analogues showing nanomolar activation potency. In HepG2 PKM2-knockout cells, the compounds increased PKL activity, maximal mitochondrial respiratory capacity and glycolytic proton efflux without altering PKL expression, Ser113 phosphorylation-dependent localisation, cell proliferation or triglyceride accumulation under the conditions examined. These findings establish symmetry-guided ligand fusion as a productive design strategy in this PKL system and provide structurally defined chemical tools for investigating the metabolic consequences of direct PKL activation.

## Abstract 2

Structure-guided exploitation of symmetry within multimeric protein interfaces offers an opportunity to convert partial ligand-binding poses into higher-affinity modulators.

## Graphical abstract

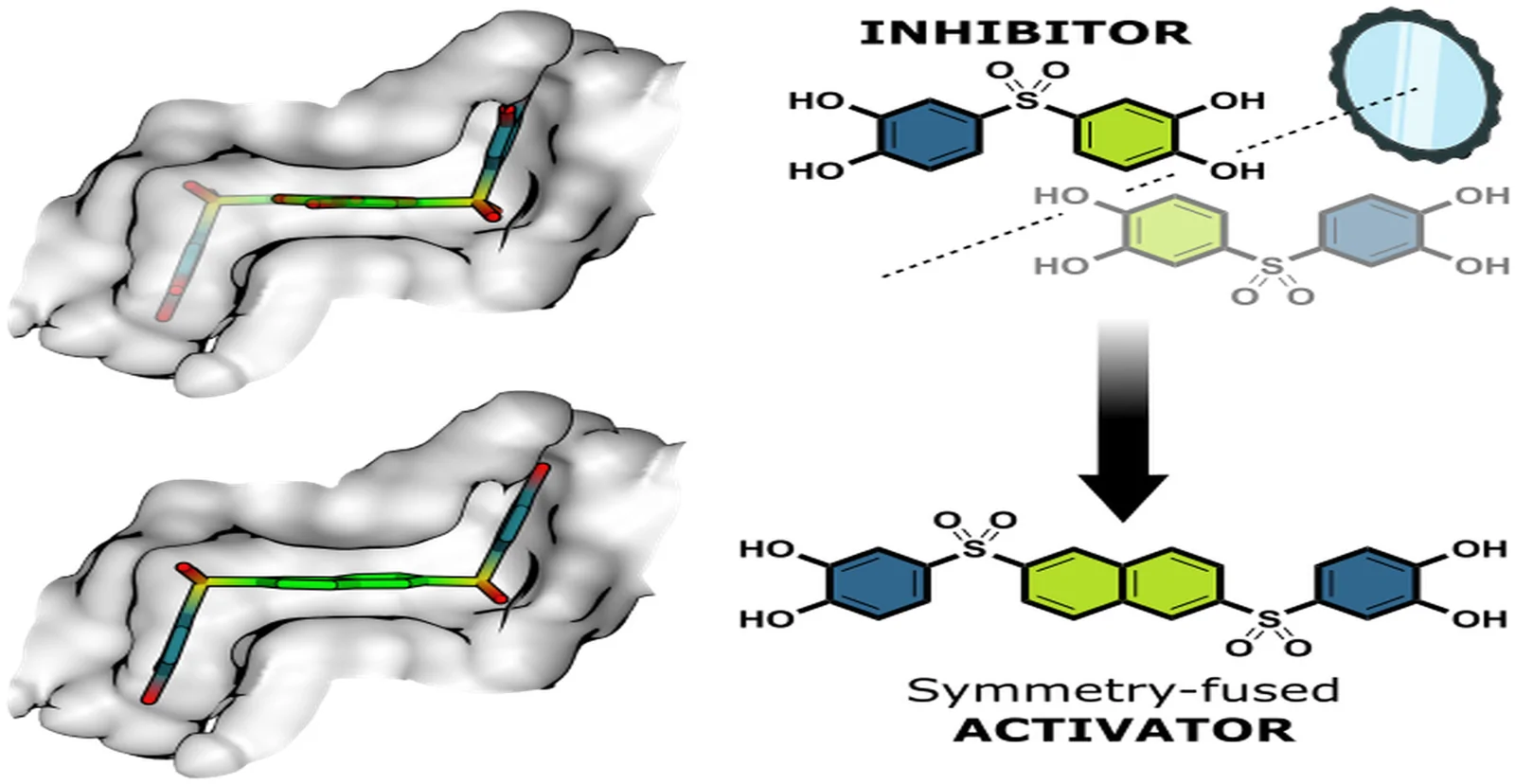

## Introduction

Human liver pyruvate kinase (PKL) is a glycolytic enzyme that plays a central role in hepatic energy regulation by catalysing the transfer of a phosphate group from phosphoenolpyruvate (PEP) to adenosine diphosphate (ADP), generating pyruvate and adenosine triphosphate (ATP).^1^ This is the rate-limiting step of glycolysis and governs the production of pyruvate, a key metabolic intermediate that fuels the tricarboxylic acid (TCA) cycle and links cytosolic glycolysis to mitochondrial oxidative metabolism.^2,3^ Consequently, PKL indirectly influences *de novo* hepatic lipogenesis, which depends on citrate generated in mitochondria.^4^ In this sense, PKL acts as a central control point coordinating glycolysis, gluconeogenesis and lipid synthesis, and its activity is tightly regulated by hormonal and nutritional signals.^5^

For example, phosphorylation by protein kinase A (PKA) during fasting decreases its catalytic efficiency and diverts PEP towards gluconeogenesis.^6^ Dysregulation of PKL has been implicated in metabolic disorders including metabolic-associated steatotic liver disease (MASLD),^7,8^ insulin resistance and type 2 diabetes, where impaired control of glycolytic and lipogenic flux contributes to metabolic dysfunction.^4,9^ A detailed mechanistic understanding of PKL regulation is therefore essential for assessing its therapeutic potential.

In mammals, pyruvate kinase exists in four different isoforms: PKL (in liver), PKR (in red blood cells), PKM2 (in embryonic and in proliferating cells), and PKM1 (in brain, in skeletal muscle, and in heart).^10,11^ Activation of PKR and PKM2 as pharmacological interventions has been explored extensively. Mutations in PKR can cause pyruvate kinase deficiency, a condition where erythrocytes are destroyed prematurely due to insufficient glycolytic ATP production.^12^ This led to the development of allosteric PKR activators such as mitapivat, approved by the U.S. Food and Drug Administration and the European Medicines Agency for treatment of pyruvate kinase deficiency,^12–15^ and etavopivat, currently in phase III clinical trials for sickle cell disease.^16^

PKM2 has also been extensively investigated as a pharmacological target because of its role in metabolic reprogramming.^17–19^ Allosteric PKM2 activation decreases the accumulation of glycolytic intermediates and can enhance mitochondrial respiration.^20^ In contrast, the downstream metabolic consequences of direct pharmacological activation of PKL remain comparatively underexplored. Whether direct activation of PKL enhances mitochondrial respiration, alters glycolytic flux, or promotes lipogenesis in hepatocytes has remained unclear. PKL can, similarly to PKM2, be phosphorylated (at Ser113 in the case of PKL) and translocate to the nucleus, although the physiological implications of this process are not fully understood.^6^ PKL overexpression has been associated with mitochondrial enrichment and increased cell growth,^8^ suggesting that its influence may extend beyond glycolysis alone. However, it remains unclear whether direct activation of PKL in hepatocytes enhances mitochondrial respiration, alters glycolytic flux, or influences lipid metabolism in a manner analogous to PKM2 activation.^8^

We have long been interested in developing PKL modulators for hepatic metabolic regulation.^21–24^ Nain-Perez *et al.* 2023, reported a series of urolithin D-derived allosteric PKL modulators targeting a cryptic site at the interface between two protomers, in which ligand geometry strongly influenced functional outcome: linear sulfone-sulfonamides acted as activators, whereas non-linear analogues were inhibitors.^22^ We subsequently identified a covalent PKL activator that modifies Cys436 and increases enzyme activity.^25^ Together, these studies established that PKL activity can be modulated through structurally and mechanistically distinct approaches and highlighted the importance of ligand binding mode in determining functional outcome.

In this work, we describe the structure-guided design of novel PKL activators, inspired by a serendipitous finding of symmetrically bound ellagic acid derivatives. Starting from inhibitor-bound crystal structures, we exploited symmetry-related binding poses within the inter-subunit pocket to design single ligands spanning both sides of the interface. Here, we outline the design, synthesis, and biochemical characterisation of these compounds. We further demonstrate that they increase mitochondrial respiration and glycolytic flux without altering PKL expression, localisation, or triglyceride accumulation in HepG2 PKM2-knockout cells under the conditions examined.

## Results and discussion

Ellagic acid has previously been identified as an allosteric inhibitor of PKL.^21^ Structural derivatisation of ellagic acid and its naturally occurring metabolite urolithin D (1) yielded sultone inhibitor 2 (IC_50_ = 3 μM, Fig. S3), which was shown by X-ray crystallography to occupy the allosteric pocket of PKL located at the dimer-dimer interface of the tetrameric enzyme (Fig. 1). The crystal structure revealed two symmetry-related binding positions for 2 flanking the two central Phe38 residues coming from two neighbouring subunits of the enzyme.

**Fig. 1:**
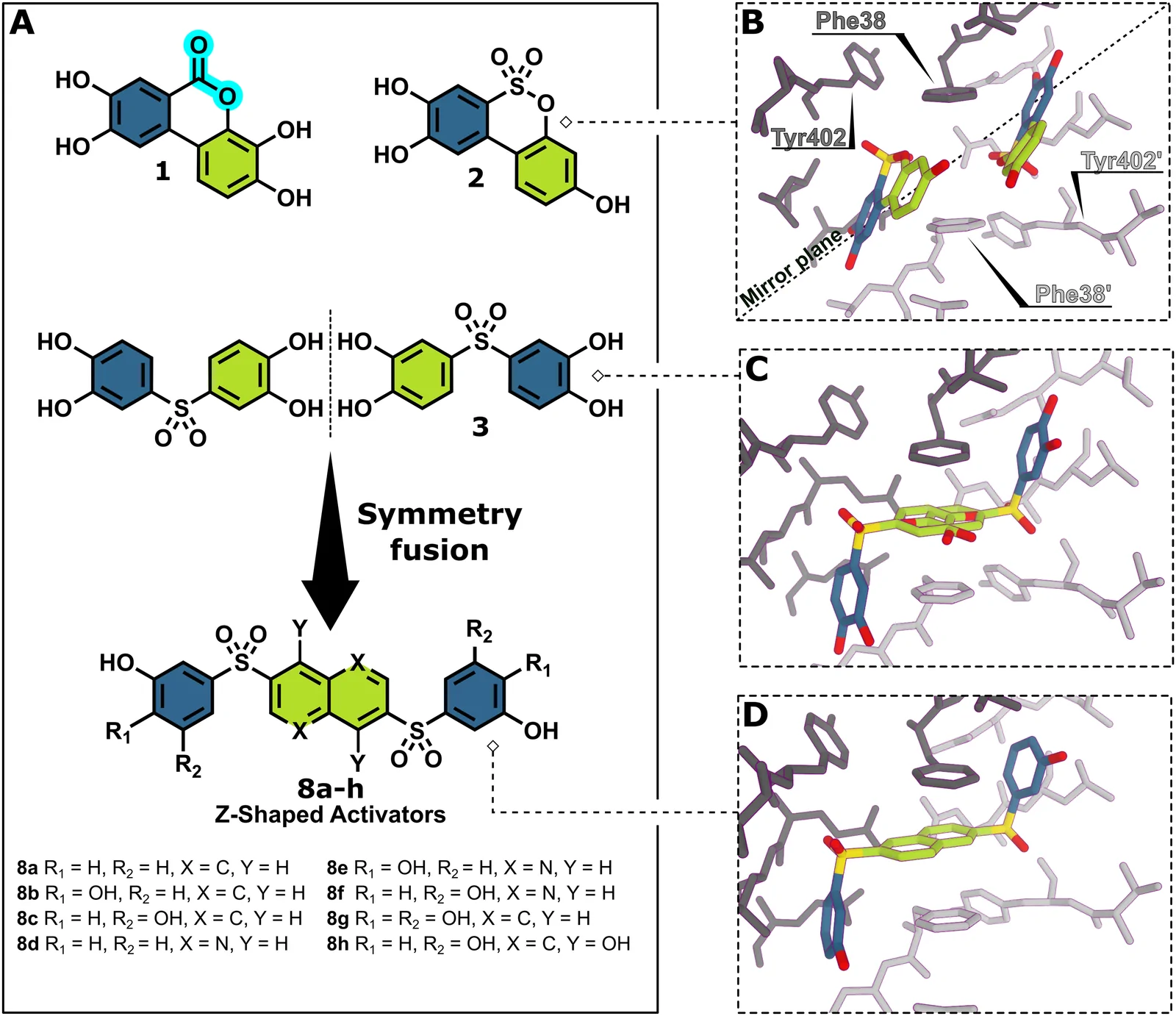
Design and structural basis of Z-shaped activators derived from Urolithin D (1). A) Bioisosteric replacement of the lactone in urolithin D with sultone afforded compound 2. Subsequent opening of the sultone ring gave sulfone 3. Fusion of symmetry-related binding poses of 3 provided a scaffold for Z-shaped activators 8a–8h. B) X-ray crystal structure of 2 in complex with PKL (PDB ID: 7FRW), showing symmetry-related binding poses across the mirror plane. C) X-ray crystal structure of 3 in complex with PKL (PDB ID: 7FRV). D) X-ray crystal structure of 8a in complex with PKL (PDB ID: 14CO).

Further structural modification, including ring opening and contraction of the sulfone linkage, produced derivatives S3, S4, and 3 (Fig. 1, S1 and S4). X-ray crystallographic analysis of these compounds revealed an altered binding configuration relative to the parent scaffold. Recombinant human PKL was expressed and purified as described in the SI. Crystallisation, X-ray diffraction data collection and structure determination are described in detail in the Supporting Information. In the case of compound 3, one aromatic ring is positioned between the side chains of two Phe38 residues, while the second ring interacts with the edge of the Phe38 side chain to form a T-shaped π–H interaction (Fig. 1C). Because the allosteric pocket exhibits twofold symmetry across the subunit interface, two molecules of 3 were observed bound at approximately equal occupancies. Superposition of these symmetry-related binding conformations suggested that fusion of the two poses could generate a single ligand spanning both sides of the pocket, with a “Z-shaped” geometry (Fig. 1 and S2). A series of fused analogues (8a–8h) were subsequently designed and synthesised to investigate the influence of hydroxyl substitution patterns and variations in the central aromatic core on activity.

To evaluate PKL activation in a cellular background without a contribution from PKM2, we used HepG2 PKM2-knockout cells. In lysates prepared from these cells, all Z-shaped compounds activated PKL following a short incubation period at ambient temperature (Fig. 2A). Compounds 8a, 8c, 8d and 8f exhibited the highest efficacy, producing approximately 250% activity at 10 μM, comparable to the positive controls (mitapivat and TEPP-46) under the same conditions. Compounds 8b and 8e displayed lower activation (approximately 150%). Following 48 h of treatment of live HepG2 PKM2 knockout cells, all compounds except 8e markedly increased PKL activity (approximately 400% activation relative to vehicle control) (Fig. 2B). Compounds 8g and 8h were excluded from testing due to chemical instability.

**Fig. 2:**
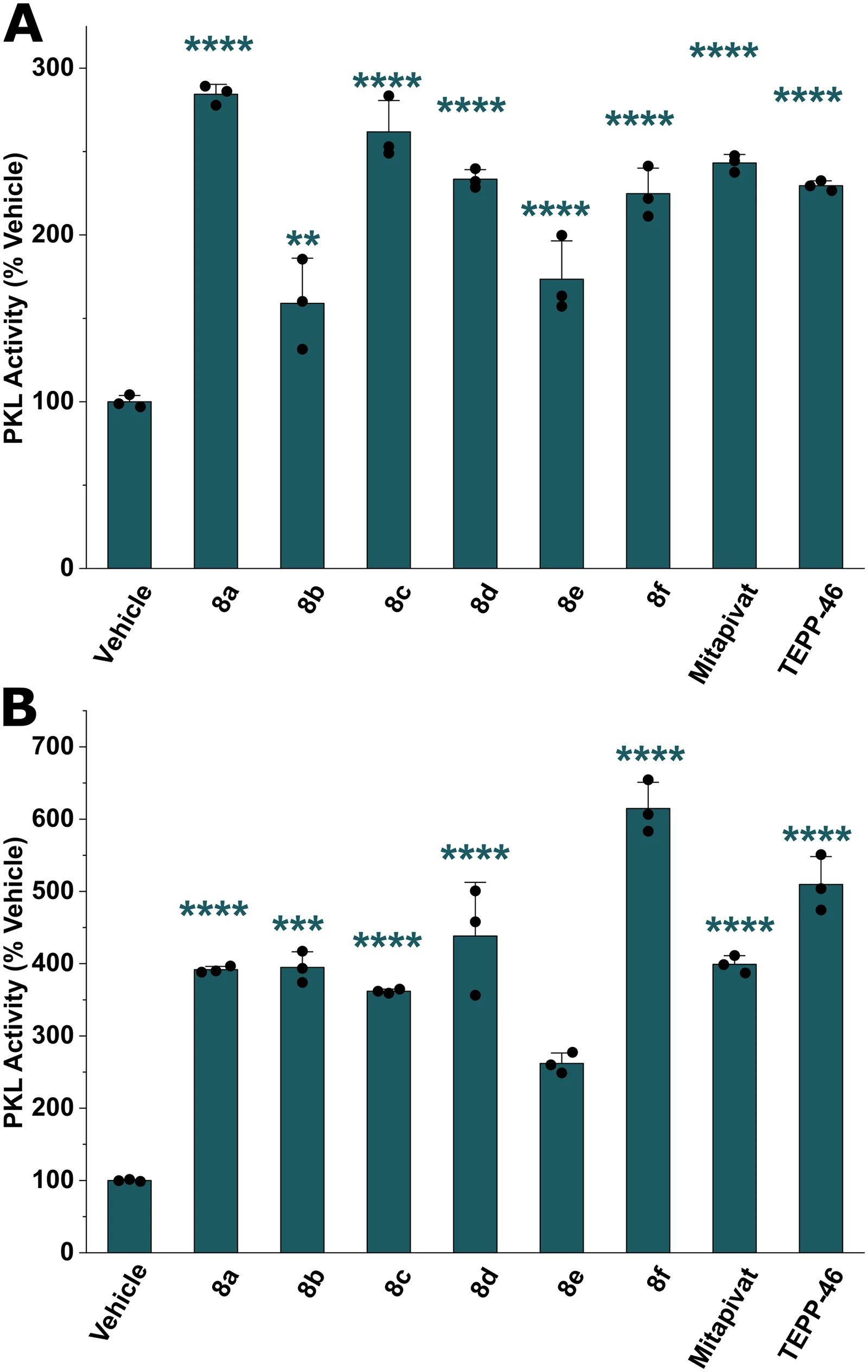
A) PKL activity of HepG2 PKM2 knockout cell lysates preincubated for 5 min at room temperature with a 10 μM solution of compounds 8a–8f, mitapivat, TEPP-46 or vehicle (DMSO) control. B) PKL activity after treatment for 48 h of HepG2 PKM2 knockout cells with a 10 μM solution of compounds 8a–8f, mitapivat, TEPP-46 or vehicle. Mean ± SD, *n* = 3, individual data points shown as black dots, **p < 0.01, ***p < 0.001, ****p < 0.0001, one-way ANOVA followed by Dunnett's multiple comparison test.

Potencies were determined using a biochemical Kinase-Glo assay (Fig. S3). Mitapivat (EC_50_ = 46 nM) exhibited potency comparable to TEPP-46 (EC_50_ = 17 nM) while displaying higher maximal activation (*E*_max_ = 514% *versus* 136%, respectively). Among the Z-shaped ligands, 8d (EC_50_ = 28 nM) and 8f (EC_50_ = 63 nM) showed nanomolar potencies, whereas 8a and 8b (EC_50_ = 297 nM and 617 nM, respectively) were less potent. Compound 8c displayed slightly higher maximal efficacy (*E*_max_ = 256%) compared with the other Z-shaped analogues (approximately 200%). Comparison of 8d with 8a, and of 8f with 8c, indicates that incorporation of a naphthyridine core enhances potency. The relatively low potency of 8b suggests that the 3,4-dihydroxy substitution pattern is less favourable for binding. The potency of 8e could not be determined owing to low solubility.

Crystal structures of PKL in complex with ligands 8a–8h were determined and revealed highly similar binding modes (Fig. 3 and S4). The Z-shaped ligands span the inter-subunit pocket, with their fused aromatic cores positioned between the Phe38 side chains. The approximately 106° angle between the central core and terminal benzene rings favours additional T-shaped π–H interactions with Phe38, while water-mediated hydrogen bonds connect the sulfonyl groups to residues including Tyr402, Asp330 and Asp366 (Fig. S2). Comparison with established pyruvate kinase activators highlights distinct ways of engaging this pocket. TEPP-46 occupies a broadly similar region despite its asymmetric structure, whereas mitapivat adopts a different orientation and forms a hydrogen bond with Lys323 (Fig. 3B and C). Thus, the crystallographic data support the intended symmetry-guided fusion strategy while demonstrating that structurally distinct activators can exploit different interaction patterns within the same inter-subunit pocket.

**Fig. 3:**
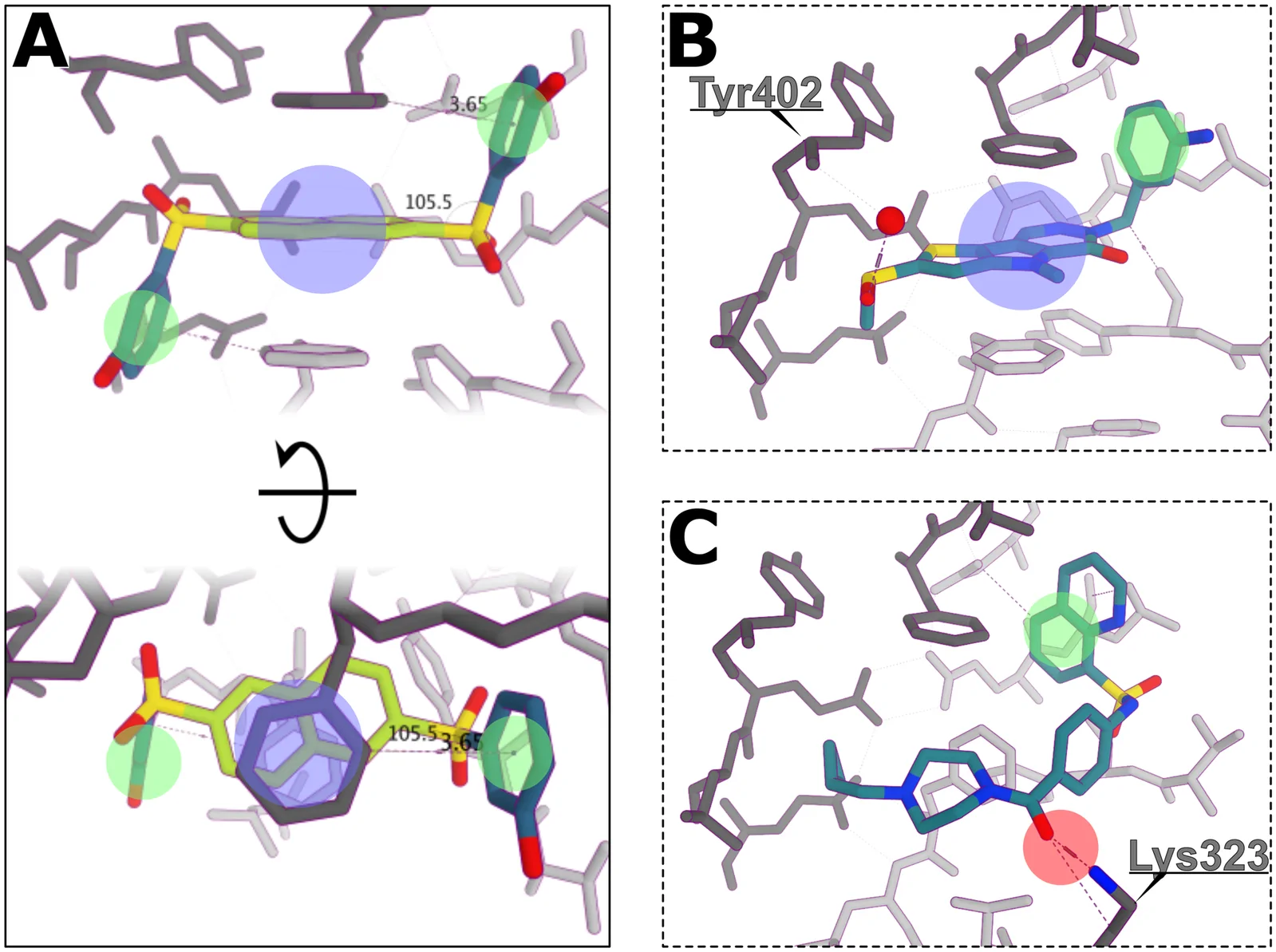
Comparison of binding poses of 8a, TEPP-46, and mitapivat. A) Side and top-down perspectives of X-ray crystal structure of 8a in complex with PKL (PDB ID: 14CO). C–S–C planar angle and distance between benzene rings of 8a and Phe38 are indicated. B) X-ray crystal structure of TEPP-46 in complex with PKL (PDB ID: 14CL). C) X-ray crystal structure of mitapivat in complex with PKL (PDB ID: 14CK). Blue spheres indicate aromatic π–π interactions between aromatic rings of ligands and side chains of Phe38 residues, while green spheres indicate T-shaped π-interactions with Phe38. Red sphere indicates hydrogen bond between carbonyl group of mitapivat and side chain of Lys323.

The isoform selectivity of the Z-shaped activators was not systematically evaluated in the present study. The human pyruvate kinase isoforms share substantial sequence and structural similarity, and the inter-subunit allosteric pocket targeted by the Z-shaped compounds is conserved between PKL and PKM2.^25,26^ Consistent with this conservation, structurally related ligands targeting this pocket have previously been shown to bind to and activate both PKL and PKM2.^27^ Accordingly, cross-reactivity of the present Z-shaped activators with PKM2 cannot be excluded. The use of PKM2-knockout HepG2 cells eliminates PKM2 as a contributor to the cellular pyruvate kinase activity measured here but should not be interpreted as evidence of molecular selectivity of the compounds for PKL over PKM2. Comprehensive biochemical profiling against the other pyruvate kinase isoforms will be required to establish the isoform-selectivity profile of this compound class.

To investigate whether activation was associated with altered oligomeric distribution, lysates from compound-treated cells were analysed by native PAGE followed by immunoblotting with PKL-specific antibodies (Fig. S5A). Three principal bands were detected. Based on their relative electrophoretic mobility and the expected molecular weight of PKL (∼58 kDa per protomer), these bands were assigned as species consistent with monomeric, dimeric and tetrameric forms of PKL.

Band intensities were quantified by densitometric analysis and expressed as the percentage of total PKL immunoreactive signal detected in each band (Fig. S5B–D). These values reflect relative protein distribution and do not correspond to particle numbers, nor do they account for molecular weight differences between oligomeric species.

Most compounds produced only modest changes in the apparent distribution of PKL among the detected bands. Compounds 8c and 8f reduced the apparent proportion of PKL signal detected in the lowest-molecular-weight band and increased signal in higher-molecular-weight species, whereas mitapivat produced a similar but less pronounced shift. A comparable enrichment of the highest-molecular-weight band was observed when recombinant PKL was pre-incubated with the compounds before native PAGE separation (Fig. S5E), and in-gel activity assays showed that catalytic activity was confined to this species (Fig. S5F).^28^

These observations could reflect ligand-induced stabilisation of higher-order PKL assemblies; however, native PAGE can promote partial dissociation or redistribution of multimeric proteins during electrophoresis.^29^ Consequently, the apparent enrichment of higher-molecular-weight species cannot be interpreted as evidence for altered oligomerisation in solution. Instead, the data are consistent with the Z-shaped ligands stabilising a catalytically competent tetramer against dissociation under the electrophoretic conditions. Future studies using solution-based approaches such as mass photometry could provide an orthogonal means of assessing ligand-dependent changes in the oligomeric distribution of recombinant PKL.

HepG2 PKM2-knockout cells were selected as a defined cellular system in which PKM2 does not contribute to the measured cellular pyruvate kinase activity, enabling the effects of compound treatment on PKL-dependent cellular metabolism to be examined without this potential confounding contribution. To assess whether PKL activation influenced mitochondrial function, HepG2 PKM2 knockout cells were treated with compounds (10 μM, 4 h) and analysed using the Seahorse Mito Stress Test (Fig. 4A). All active Z-shaped ligands increased maximal mitochondrial respiration relative to the vehicle control following FCCP treatment (Fig. 4B).

**Fig. 4:**
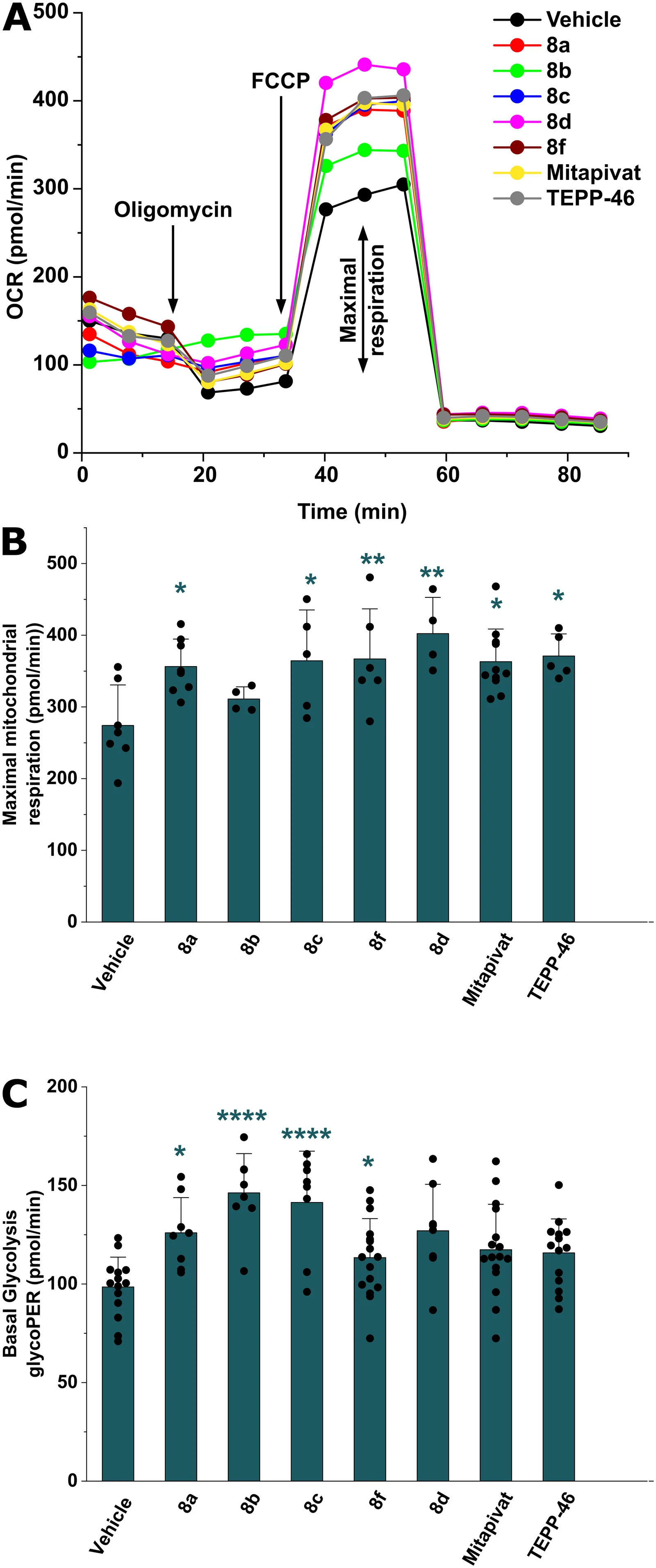
A) Oxygen consumption rate profile (OCR) as assessed by Seahorse Mito Stress Test after treatment of HepG2 PKM2 knockout cells for 4 h with 10 μM solution of the compounds 8a–8f, mitapivat, TEPP-46 or vehicle (DMSO) control. B) Maximal mitochondrial respiration calculated after the test performed as in A, *n* = 4–8. C) Glycolytic proton efflux rate (glycoPER), calculated after performing Seahorse Glycolytic rate assay test on HepG2 PKM2 knockout cells treated with the compounds as in A, mean ± SD, *n* = 8–16, individual data points shown as black dots, *p < 0.05, **p < 0.01, ****p < 0.0001, One-way ANOVA followed by Dunnett's multiple comparison test.

Glycolytic proton efflux rate (glycoPER) was determined using the Seahorse Glycolytic Rate Assay. This assay calculates glycolytic proton efflux from extracellular acidification and oxygen-consumption measurements after correction for mitochondrial CO_2_-derived acidification, providing a quantitative functional readout of glycolytic activity. Treatment with compounds 8a–8d increased glycoPER (Fig. 4C), indicating increased glycolytic proton efflux under the experimental conditions examined.

Given that increased glycolytic flux could potentially increase the supply of carbon for *de novo* lipogenesis,^30^ we examined whether PKL activation influenced triglyceride (TG) accumulation under steatotic conditions. HepG2 PKM2 knockout cells were cultured for seven days in lipid-inducing medium in the presence of compounds (10 μM). Lipidomic analysis of TG species revealed no significant differences relative to vehicle control (Fig. S6). Thus, the increased glycolytic and mitochondrial activity observed following PKL activation was not accompanied by increased triglyceride accumulation under the conditions tested.

No effects on cell proliferation were observed after 72 h with 10 μM compounds (Fig. S7A). Quantitative PCR analysis demonstrated minimal changes in PKL mRNA expression, with slight reductions observed only for 8f and mitapivat (Fig. S7B).^31–33^ Furthermore, none of the compounds altered PKL Ser113 phosphorylation or its cytosolic-nuclear distribution (Fig. S7D and E and S8). Together, these observations support a direct effect of the compounds on PKL activity rather than an indirect effect mediated by altered PKL expression or localisation.

Overall, pharmacological PKL activation in HepG2 PKM2-knockout cells was associated with increased glycolytic flux and mitochondrial respiratory capacity without increased triglyceride accumulation. Although glycoPER provides a quantitative functional readout of glycolytic proton efflux, it does not directly determine intracellular pyruvate or lactate concentrations, lactate production, or the partitioning of pyruvate between mitochondrial oxidation and lactate formation. Accordingly, the present data do not establish how compound-mediated PKL activation affects pyruvate fate. Direct targeted metabolite measurements and isotope-tracing studies will be required to resolve these questions. The cellular studies were performed in HepG2 PKM2-knockout cells. This model provides a defined cellular context in which PKM2 does not contribute to the measured pyruvate kinase activity and was therefore suitable for establishing whether the lead compounds elicit metabolic effects consistent with PKL activation. However, as a single transformed hepatic cell model, it cannot establish whether these effects extend to primary hepatocytes or other hepatic systems. Validation in primary human hepatocytes and complementary hepatic models will be required to define the broader physiological relevance of the present findings.

## Conclusions

In summary, we have identified a novel class of symmetrical “Z-shaped” ligands that act as potent allosteric activators of human PKL. These compounds were derived from structural analysis of inhibitor-bound complexes and rationally designed through symmetry-guided fusion of binding poses within the inter-subunit allosteric pocket at the dimer-dimer interface. This approach unexpectedly transformed an inhibitor-derived scaffold into activators.

Biochemical and cellular studies demonstrate that the Z-shaped ligands robustly enhance PKL activity in HepG2 PKM2 knockout hepatocytes. The structural and native-PAGE data are consistent with ligand-induced stabilisation of a catalytically competent tetrameric conformation, although the latter does not establish changes in oligomeric state in solution.

In the defined HepG2 PKM2-knockout cellular model, compound-mediated PKL activation increased glycolytic proton efflux and maximal mitochondrial respiratory capacity without altering PKL expression or localisation, cell proliferation, or triglyceride accumulation under the conditions examined.

Collectively, these findings show that compound-mediated PKL activation was associated with increased glycolytic proton efflux and maximal mitochondrial respiratory capacity in HepG2 PKM2-knockout cells, without increased triglyceride accumulation under the conditions examined. The present experiments do not resolve pyruvate partitioning between mitochondrial oxidation and lactate formation, and whether these effects are reproduced in primary hepatocytes or other physiologically relevant hepatic systems remains to be established. These “Z-shaped” ligands therefore provide a foundation for further optimisation and offer new insight into the metabolic consequences of direct PKL activation. More broadly, these findings illustrate the potential of exploiting symmetry-related ligand poses as a structure-guided design strategy for ligands targeting inter-subunit pockets in multimeric proteins. Whether this approach is transferable to other protein systems will require further investigation.

## Author contributions

Conceptualisation: L. M., A. R. R., M. G., M. H. and J. B.; Methodology: L. M., A. R. R., A. F. F., O. N., L. H., A. L., P. B., M. R. and P.-O. B.; Validation: all; Data interpretation: all; Writing - original draft: O. N.; Writing - review & editing: all; All authors have read and agreed to the published version of the manuscript.

## Conflicts of interest

Authors J. B. and A. M. are co-owners and board members of ScandiEdge Therapeutics AB, which partly funded this study. All other authors declare no competing interests.

## Supplementary Material

MD-OLF-D6MD00604C-s001

## Data Availability

The data supporting this article have been included as part of the supplementary information (SI). Supplementary information: the SI contains experimental procedures, compound synthesis and characterisation, protein expression and purification, crystallisation and structure-determination methods, and supplementary structural, biochemical and cellular data. See DOI: https://doi.org/10.1039/d6md00604c.
