# Supplementary material for: Symmetry-guided fusion converts weak liver pyruvate kinase inhibitors into potent allosteric activators
Source: RSC Med Chem. 2026 Sep 24. Online ahead of print. doi: 10.1039/d6md00604c (PMC13667364; doi:10.1039/d6md00604c)
Supplement: Supplementary file 1 [file MD-OLF-D6MD00604C-s001.pdf]

Supplementary Information (SI) for RSC Medicinal Chemistry.
This journal is © The Royal Society of Chemistry

Supplementary information

Symmetry-Guided Fusion Converts Weak Liver Pyruvate Kinase inhibitors into potent Allosteric Activators

Leticia Monjas [a†], Alexandra Rodriguez Rivero [a†], Anders Foller Füchtbauer [a†], Oscar Nilsson [a], Liliana Håversen [b], Aleksei Lulla [c], Paul Brear [c], Mikael Rutberg [b], Per-Olof Bergh [b], Jan Borén [b], Adil Mardinoglu [d], Marko Hyvönen [c] and Morten Grøtli [a*]

[a.] *Department of Chemistry and Molecular Biology, University of Gothenburg Medicinaregatan 7b, SE-413 90, Gothenburg, Sweden*

[b.] *Department of Molecular and Clinical Medicine, University of Gothenburg and Sahlgrenska University Hospital, Bruna Stråket 16, SE-413 45, Gothenburg, Sweden*

[c.] *Department of Biochemistry, University of Cambridge, Tennis Court Road 80, Cambridge, CB2 1GA, United Kingdom*

[d.] *Science for Life Laboratory, KTH - Royal Institute of Technology, Tomtebodavägen 23, SE-171 65, Stockholm, Sweden*

† These authors contributed equally to this work

Contents

General information 3

Synthesis and compound characterisation 4

General synthetic procedures 5

Compound synthesis and characterisation 6

Supplementary figures and tables 14

Experimental procedures 25

Cell culture 25

Determination of PKL activity after incubation of the compounds with cell lysates 25

Determination of PKL activity after treatment of the cells with the compounds 26

Measurement of oxygen consumption rate and glycolytic rate using Seahorse 26

mRNA Gene expression 26

Cell proliferation 26

PKL and p-PKL (Ser113) determination in cytoplasmic and nuclear fractions 26

Steatosis model and triglyceride determination 27

Gel-electrophoresis experiments 27

Determination of in-gel PKL activity 27

Native PAGE and immunoblotting for determination of PKL oligomerization 27

Determination of PKL by luminescence assay 28

Statistical analysis 28

Protein production 28

X-ray crystallography 29

NMR spectra 30

Abbreviations 59

References 59

General information

Unless otherwise specified, all reagents were obtained from commercial supplier Sigma-Aldrich and used without further purification. Solvents were dried on a solvent purification system (PS-MD-5/7 Inert Technology). Reactions were monitored by LC-MS (Perkin Elmer Series 200; Waters Symmetry C8 column 3.5 μm, 4.6 x 50 mm; H_2O:CH_3CN (0.1% formic acid)) or by thin-layer chromatography (TLC) on silica-gel-coated aluminium foils (silica gel 60 F254, Merck). The TLC plates were visualized by UV light (λ = 254 nm). Flash-column chromatography was performed on an Isolera One or Selekt flash chromatography systems (Biotage), using silica gel SNAP KP-Sil, Sfär cartridges or reversed phase Sfär C18 columns. Melting points were determined with a Büchi B-545 apparatus. NMR spectra were recorded on a Varian NMR 400 spectrometer at 25 °C. All chemical shifts are reported in parts per million (δ) relative to the residual solvent peak. The following abbreviations are used to denote signal patterns: (s) singlet, (d) doublet, (t) triplet, (q) quartet, (m) multiplet, and (br) broad, unless otherwise noted. Coupling constants (*J*) are reported in Hertz (Hz). High resolution mass spectra (HRMS) were recorded on an Agilent 1290 infinity LC system tandem to an Agilent 6520 Accurate Mass Q-TOF spectrometer.

Synthesis and compound characterisation

The ligands were synthesised from commercially available 2,6-dibromonaphthalene (**4a**), 1,5-naphthyridine (**4b**) and 2,6-dibromonaphthalene-1,5-diol (**4c**) (Scheme S1). Compounds **4a** and **4b** were converted into 2,6-diiodonaphthalene (**5a**) and 3,7-dibromo-1,5-naphthyridine (**5b**), respectively, to enable installation of thioethers via palladium-catalysed thiol coupling or nucleophilic aromatic substitution. Dibromonaphthalene-1,5-diol was first methylated affording **4c**, following halogen exchange to yield intermediate **5c**. Intermediates **5a**, **5b** and **5c** were reacted with 3-methoxythiophenol, 3,4-dimethoxythiophenol 3,5-dimethoxythiophenol (**S2**), or 3,4,5-trimethoxybenzenethiol to yield eight symmetrical thioethers (**6a-h**). Due to its limited availability, **S2** was prepared by a Newman-Kwart rearrangement of O-(3,5-dimethoxyphenyl) dimethylcarbamothioate (**S1**), which was synthesised via the coupling of 3,5-dimethoxyphenol with dimethylthiocarbamoyl chloride. Compounds **6a-h** were subsequently oxidised to the corresponding sulfones **7a-h** using excess *m*CPBA. The oxidation reactions proceeded with good to excellent yields for all substrates and generated the intermediate products without the need for preparative chromatography. Finally, the deprotection of the methoxy groups of **7a-h** was conducted via reactions with BBr_3 in dichloromethane (DCM). The reactions were generally inefficient, requiring a large excess of reagent and a long time to run to completion. The poor solubility of intermediates **7a-h** was likely a contributing factor. Compound **7f** was notably insoluble in most common solvents, including dichloromethane, ethyl acetate, acetonitrile, methanol, acetone, toluene, diethyl ether, and tetrahydrofuran. After the workup of the deprotection reactions and trituration of the products using suitable solvent systems, the final products **8a-h** were obtained in moderate to good yields.

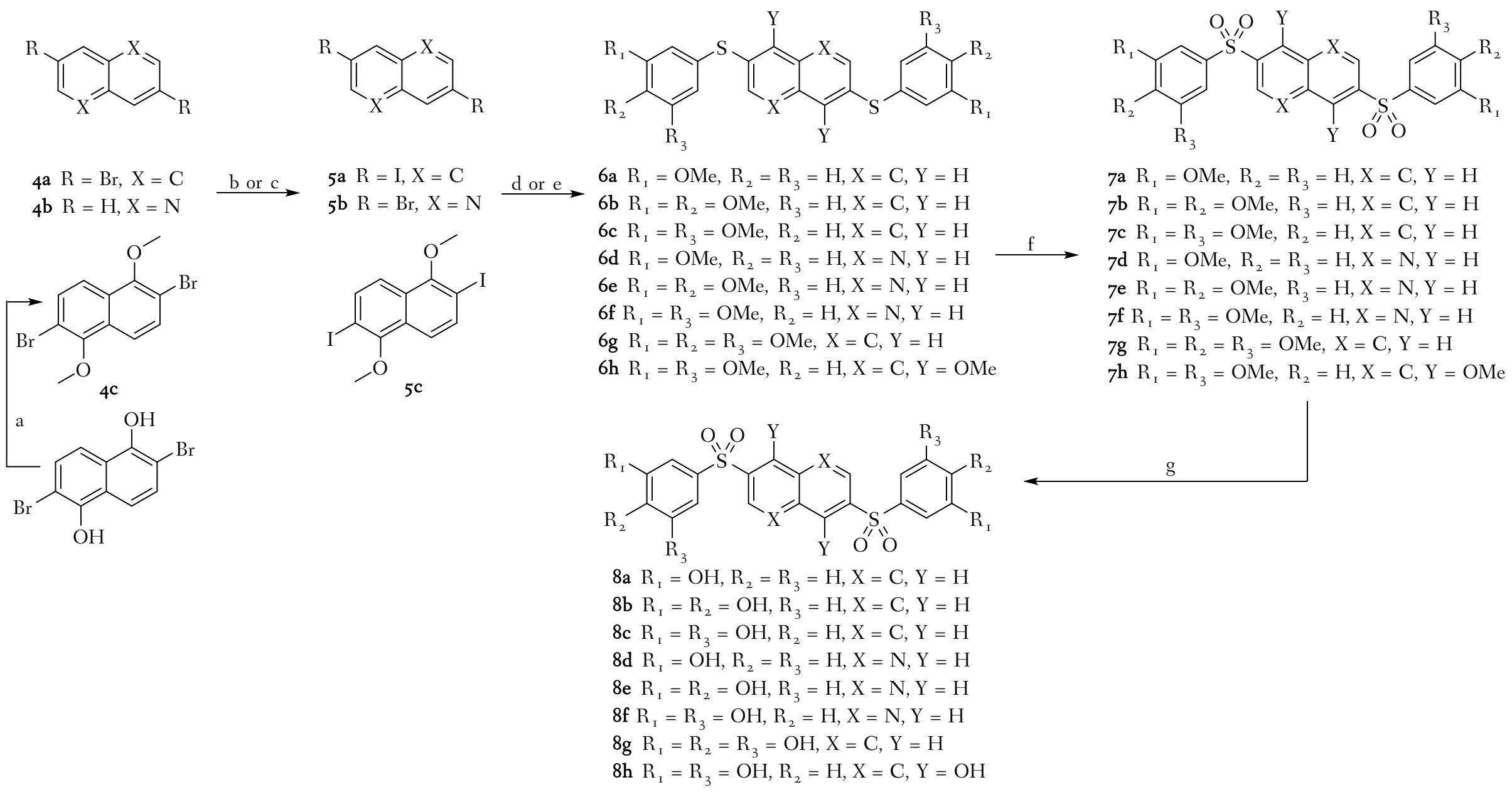

Scheme S1: Synthesis of Z-shaped activators: a) MeI (8 eq.), KOH (8 eq.), DMSO, r.t., 20 h; b) *t*-BuLi (3 eq.), I_2 (3 eq.), THF, -78 °C to r.t., 18 h; c) Br_2 (2.1 eq.), NaOAc (2.1 eq.), AcOH, 60 °C, 21 h; d) thiophenol (2.2 eq.), $Pd_2(dba)_3$ (2 mol%), dppf (4 mol%), DIPEA (2.6 eq.), toluene, 120 °C, 16-18 h; e) thiophenol (2.2 eq.), KOH (5 eq.), DMSO/H_2O (1:1), 100 °C, 18 h; f) mCPBA (5-6 eq.), chloroform, r.t., 20-24 h; and g) BBr_3 (4-20 eq.), DCM, 0 °C to r.t., 16-96 h.

General synthetic procedures

I – Palladium-catalysed synthesis of naphthalene thioethers

A solution (or suspension) of **5a** (1 eq.), the corresponding thiophenol (2.2 eq.), DPPF (4 mol%), $Pd_2(dba)_3$ (2 mol%) and DIPEA (2.6 eq.) in toluene (0.15 M) was stirred at 120 °C for 16-18 h (reaction monitored by LCMS). Then the solvent was removed under reduced pressure. H_2O (40 mL) and DCM (40 mL) were added to the residue, the layers were separated, and the aqueous layer was extracted with DCM (2 x 40 mL). The combined organic layers were dried over Na_2SO_4, filtered, and concentrated under reduced pressure. Purification by flash column chromatography afforded the products.

II – Synthesis of naphthyridine thioethers by nucleophilic aromatic substitution

A suspension of **5b** (1 eq.), thiophenol (2.2 eq.) and KOH (5 eq.) in DMSO/H_2O (1:1, 0.3 M) was stirred at 100 °C for 18 h and monitored by LC-MS and TLC. The reaction mixture was partitioned between DCM (10 mL) and distilled H_2O (10 mL). The organic fraction was isolated, the aqueous fraction was extracted with DCM (2 x 5 mL), the organic fractions were combined, washed with NaOH solution (0.5 M, 10 mL), distilled H_2O (2 x 10 mL), brine (10 mL), dried over anhydrous Na_2SO_4, filtered, and concentrated under reduced pressure to afford the products which were used in the next step without further purification.

III – Oxidation of sulfides into sulfones

To a solution of thioether **6** in chloroform (0.03-0.05 M), *m*CPBA (≥ 77% purity, 5-6 eq.) was added. The reaction was stirred at r.t. for 20-24 h and monitored by LC-MS and TLC until complete conversion. An aqueous solution of Na_2SO_3 (10% w/v, 50 mL) and chloroform (50 mL) were added and the layers were separated. The organic layer was washed with a saturated aqueous solution of $NaHCO_3$ (2 x 40 mL), the combined organic layers were dried over Na_2SO_4, filtered and concentrated under reduced pressure to afford the crudes which were purified by trituration or flash column chromatography.

IV – Demethylation of methoxy groups

To a solution of starting material **7** in DCM (0.04-0.05 M) was added BBr_3 (1M in DCM, 4-20 eq.) dropwise at 0 °C or r.t., and the reaction was stirred at r.t. for 16-96 h until complete deprotection as indicated by LC-MS or TLC. The reactions were quenched by dropwise addition of MeOH or distilled H_2O, the reaction mixture was concentrated under reduced pressure and the crude products were purified by trituration using suitable solvent systems, or by preparative TLC.

Compound synthesis and characterisation

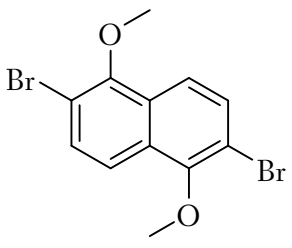

2,6-Dibromo-1,5-dimethoxynaphthalene (4c). A suspension of powdered KOH (3.26 g, 50.3 mmol, 8 eq.) in DMSO (10 mL) was stirred at r.t. for 5 min. 2,6-Dibromonaphthalene-1,5-diol (2 g, 6.29 mmol, 1 eq.) and iodomethane (3.2 mL, 50.3 mmol, 8 eq.) were sequentially added, and the reaction mixture was stirred at r.t. for 20 h and monitored by GC-MS. H_2O (100 mL) was added, the brown precipitate was collected by vacuum filtration, washed with water and dried. Purification by flash column chromatography (*n*-pentane/EtOAc 100:0 to 80:20) afforded **4c** as a yellow solid (1.72 g, 4.96 mmol, 79%); m.p.: 153-154 °C; ^{1}H-NMR (400 MHz, $CDCl_3$) δ 7.78 (d, *J* = 8.8 Hz, 2H), 7.63 (d, *J* = 8.8 Hz, 2H), 3.99 (s, 6H); ^{13}C-NMR (101 MHz, $CDCl_3$) δ 153.7, 131.3, 129.9, 119.7, 113.8, 61.8.

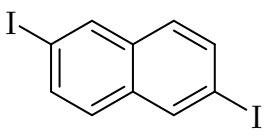

2,6-Diiodonaphthalene (5a). 2,6-Dibromonaphthalene (1.50 g, 5.25 mmol, 1 eq.) was added to a dried flask under an atmosphere of nitrogen, and anhydrous THF (30 mL) was added. The solution was cooled down to -78 °C, and t-BuLi (1.7 M in pentane, 9.3 mL, 15.7 mmol, 3 eq.) was added dropwise. The reaction mixture was stirred at -78 °C for 1.5 h. Then, a solution of iodine (4.00 g, 15.7 mmol, 3 eq.) in anhydrous THF (20 mL) was added dropwise at -78 °C, and the reaction mixture was stirred from -78 °C to r.t. for 18 h. Then, the reaction was quenched with an aqueous solution of Na_2SO_3 (10% w/v, 50 mL). The solvent was removed under reduced pressure and the residue was suspended in H_2O (50 mL) and extracted with EtOAc (3 x 50 mL), the combined organic layers were concentrated under reduced pressure and dried by co-evaporation with toluene (x2). Purification by flash column chromatography (n-pentane:EtOAc 10:0 to 8:2) afforded **5a** as a beige solid (652 mg, 1.716 mmol, 33%); m.p.: 204-206 °C; ^{1}H NMR (400 MHz, $CDCl_3$) δ 8.19 (d, J = 2.0 Hz, 2H), 7.73 (dd, J = 8.5, 2.0 Hz, 2H), 7.45 (d, J = 8.5 Hz, 2H); ^{13}C NMR (101 MHz, $CDCl_3$) δ 136.8, 135.4, 133.7, 128.4, 92.2.

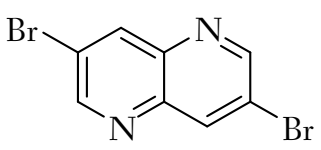

3,7-Dibromo-1,5-naphthyridine (5b). To a 250 mL round-bottom flask equipped with a stir bar was added 1,5-naphthyridine (8.06 g, 61.9 mmol, 1.0 eq.), NaOAc (10.67 g, 130.1 mmol, 2.1 eq.) and glacial AcOH (160 mL). The flask was sealed with a rubber septum and stirred at r.t. for 10 min. Bromine (6.67 mL, 130 mmol, 2.1 eq.) was added dropwise at r.t. over 20 min and the reaction was stirred at 60 °C for 21 h until completion as indicated by LC-MS. The flask was cooled down to r.t., during which a yellow precipitate formed. The solids were collected by filtration through suction filtration, washed with glacial AcOH (50 mL), saturated $Na_2S_2O_3$ (50 mL), NaOH solution (1M, 50 mL), and distilled H_2O (3 x 50 mL). Drying for 15 min under reduced pressure afforded the crude which was purified by repeated recrystallization from toluene to yield the product as white crystals (6.56 g, 22.8 mmol, 37%); R_f = 0.48 (THF/toluene 1:19); m.p.: 220-224 °C; ^{1}H NMR (400 MHz, $CDCl_3$) δ 8.99 (d, *J* = 2.1 Hz, 2H), 8.57 (d, *J* = 2.1 Hz, 2H); ^{13}C NMR (101 MHz, $CDCl_3$) δ 153.4, 142.4, 139.0, 120.8; LC-MS m/z (ESI) 98% integral, found 288.8 (M+1) $C_8H_4Br_2N_2$ requires 288.872.

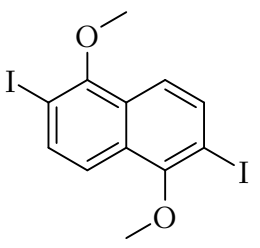

2,6-Diiodo-1,5-dimethoxynaphthalene (5c). **4c** (1 g, 2.89 mmol, 1 eq.) was added to a dried flask under an atmosphere of nitrogen, and anhydrous THF (35 mL) was added. The solution was cooled down to -78 °C, and *t*-BuLi (1.7 M in pentane, 8.5 mL, 14.5 mmol, 5 eq.) was added dropwise. The reaction mixture was stirred at -78 °C for 1 h. Then, a solution of iodine (2.58 g, 10.1 mmol,

3.5 eq.) in anhydrous THF (15 mL) was added dropwise at -78 °C, and the reaction mixture was stirred from -78 °C to r.t. for 2 h and monitored by GC-MS. The reaction was quenched with an aqueous solution of Na_2SO_3 (10% w/v, 80 mL). The solvent was removed under reduced pressure and the residue was suspended in H_2O (50 mL) and extracted with DCM (3 x 50 mL), the combined organic layers were dried over anhydrous Na_2SO_4, filtered, and concentrated under reduced pressure. The crude solids were triturated with Et_2O to afford **5c** as a pale-yellow solid (743 mg, 1.69 mmol, 58%); m.p.: 197-199 °C; ^{1}H-NMR (400 MHz, $CDCl_3$) δ 7.81 (d, *J* = 8.8 Hz, 2H), 7.63 (d, *J* = 8.8 Hz, 2H), 3.95 (s, 6H); ^{13}C-NMR (101 MHz, $CDCl_3$) δ 157.0, 136.3, 129.9, 120.3, 88.5, 61.9.

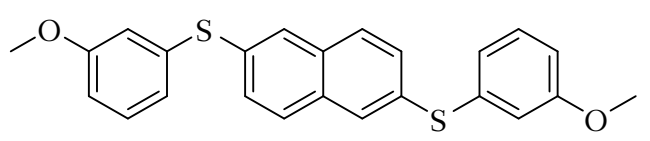

2,6-bis((3-methoxyphenyl)thio)naphthalene (6a). The reaction was performed according to general procedure ***I*** employing naphthalene **5a** (220 mg, 0.579 mmol, 1 eq.) and 3-methoxythiophenol (179 mg, 1.274 mmol, 2.2 eq.). Purification by flash column chromatography (*n*-pentane/EtOAc 10:0 to 6:4) afforded **6a** (193 mg, 0.477 mmol, 82%) as a beige/pale orange solid; R_f = 0.72 (*n*-pentane/EtOAc 19:1); m.p.: 133-135 °C; ^{1}H NMR (400 MHz, $CDCl_3$) δ 7.79 (d, *J* = 1.9 Hz, 2H), 7.66 (d, *J* = 8.5 Hz, 2H), 7.40 (dd, *J* = 8.5, 1.9 Hz, 2H), 7.23 (t, *J* = 8.3 Hz, 2H), 6.97-6.94 (m, 2H), 6.92-6.91 (m, 2H), 6.81 (ddd, *J* = 8.3, 2.5, 0.9 Hz, 2H), 3.76 (s, 6H); ^{13}C NMR (101 MHz, $CDCl_3$) δ 160.3, 136.9, 133.5, 132.8, 130.2, 129.7, 129.6, 128.5, 123.4, 116.4, 113.2, 55.5; HRMS (ESI+) m/z calcd. for $C_{24}H_{20}O_2S_2$: 404.0905 $[M]^+$; found 404.0916.

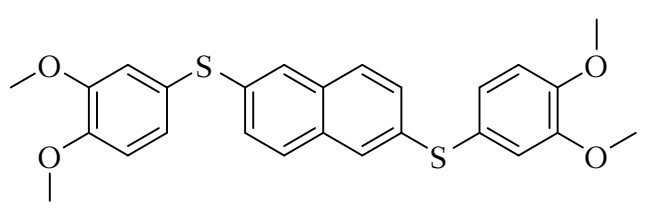

2,6-bis((3,4-dimethoxyphenyl)thio)naphthalene (6b). The reaction was performed according to general procedure ***I*** employing naphthalene **5a** (225 mg, 0.592 mmol, 1 eq.) and 3,4-dimethoxythiophenol (222 mg, 1.303 mmol, 2.2 eq.). Purification by flash column chromatography (*n*-pentane/EtOAc 10:0 to 7:3) afforded **6b** (40 mg, 0.086 mmol, 15%) as an orange-brown solid; R_f = 0.64 (*n*-pentane/EtOAc 19:1); m.p.: 180-182 °C; ^{1}H NMR (400 MHz, $CDCl_3$) δ 7.55 (d, *J* = 8.6 Hz, 2H), 7.53 (d, *J* = 1.8 Hz, 2H), 7.26 (dd, *J* = 8.6, 1.8 Hz, 2H), 7.09 (dd, *J* = 8.3, 2.1 Hz, 2H), 7.01 (d, *J* = 2.1 Hz, 2H), 6.87 (d, *J* = 8.3 Hz, 2H), 3.90 (s, 6H), 3.82 (s, 6H); ^{13}C NMR (101 MHz, $CDCl_3$) δ 149.6, 149.6, 135.7, 132.2, 127.9, 127.4, 126.8, 126.3, 124.6, 116.6, 111.9, 56.1, 56.1; HRMS (ESI+) m/z calcd. for $C_{26}H_{24}O_4S_2$: 464.1116 $[M]^+$; found 464.1104.

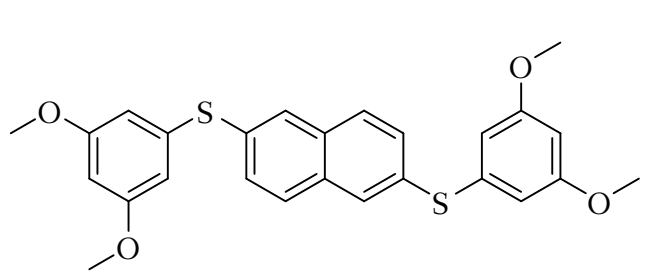

2,6-bis((3,5-dimethoxyphenyl)thio)naphthalene (6c). The reaction was performed according to general procedure ***I*** employing naphthalene **5a** (212 mg, 0.558 mmol, 1 eq.) and thiophenol **S2** (209 mg, 1.228 mmol, 2.2 eq.). Purification by flash column chromatography (*n*-pentane/EtOAc 10:0 to 8:2) afforded **6c** (194 mg, 0.418 mmol, 75%) as a beige/pale orange solid; R_f = 0.65 (*n*-pentane/EtOAc 19:1); m.p.: 135-137 °C; ^{1}H NMR (400 MHz, $CDCl_3$) δ 7.81 (d, *J* = 1.8 Hz, 2H), 7.67 (d, *J* = 8.5 Hz, 2H), 7.42 (dd, *J* = 8.5, 1.8 Hz, 2H), 6.51 (d, *J* = 2.2 Hz, 4H), 6.36 (t, *J* = 2.2 Hz, 2H), 3.74 (s, 12H); ^{13}C NMR (101 MHz, $CDCl_3$) δ 161.3, 137.7, 133.2, 132.8, 130.0, 129.7, 128.5, 108.7, 99.8, 55.6; HRMS (ESI+) m/z calcd. for $C_{26}H_{24}O_4S_2$: 464.1116 $[M]^+$; found 464.1108.

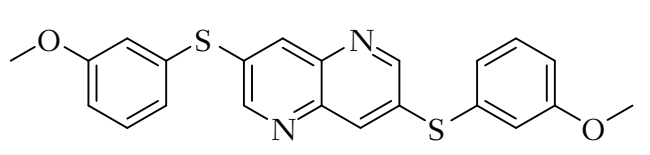

3,7-bis((3-Methoxyphenyl)thio)-1,5-naphthyridine (6d). The reaction was performed according to general procedure ***II*** employing naphthyridine **5b** (100 mg, 0.347 mmol, 1 eq.) and 3-methoxythiophenol (107 mg, 0.764 mmol, 2.2 eq.) to afford **6d** (136 mg, 0.335

mmol, 96%) as a yellow solid; R_f = 0.39 (*n*-pentane/EtOAc 7:1); m.p.: 146-148 °C; ^{1}H NMR (400 MHz, $CDCl_3$) δ 8.74 (d, *J* = 1.7 Hz, 2H), 8.14 (d, *J* = 9.5 Hz, 2H), 7.36 - 7.30 (m, 2H), 7.10 - 7.06 (m, 2H), 7.05 - 7.00 (m, 2H), 6.94 (dtd, *J* = 8.3, 2.4, 0.9 Hz, 2H), 3.80 (s, 6H); ^{13}C NMR (101 MHz, $CDCl_3$) δ 160.6, 151.9, 141.8, 135.6, 135.3, 132.9, 130.9, 125.4, 118.5, 114.9, 55.5; HRMS (ESI+) m/z calcd. for $C_{22}H_{18}N_2O_2S_2$: 407.0888 [M+H]$^+$; found 407.0891.

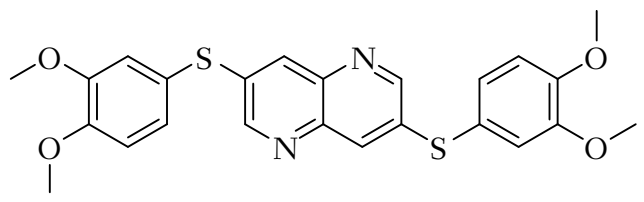

3,7-bis((3,4-Dimethoxyphenyl)thio)-1,5-naphthyridine (6e). The reaction was performed according to general procedure ***II*** employing naphthyridine **5b** (100 mg, 0.347 mmol, 1 eq.) and 3,4-dimethoxythiophenol (130 mg, 0.764 mmol, 2.2 eq.) to afford **6e** (110 mg, 0.246 mmol, 68%) as a yellow solid; R_f = 0.10 (*n*-pentane/EtOAc 7:1); m.p.: 195-196 °C; ^{1}H NMR (400 MHz, $CDCl_3$) δ 8.67 (d, *J* = 2.3 Hz, 2H), 7.82 (d, *J* = 2.3 Hz, 2H), 7.16 (dd, *J* = 8.3, 2.1 Hz, 2H), 7.03 (d, *J* = 2.1 Hz, 2H), 6.91 (d, *J* = 8.3 Hz, 2H), 3.91 (s, 6H), 3.84 (s, 6H); ^{13}C NMR (101 MHz, $CDCl_3$) δ 150.59, 150.53, 150.1, 141.5, 137.3, 133.0, 128.2, 121.1, 117.4, 112.3, 56.18, 56.15; HRMS (ESI+) m/z calcd. for $C_{24}H_{22}N_2O_4S_2$: 467.1099 [M+H]$^+$; found 467.1116.

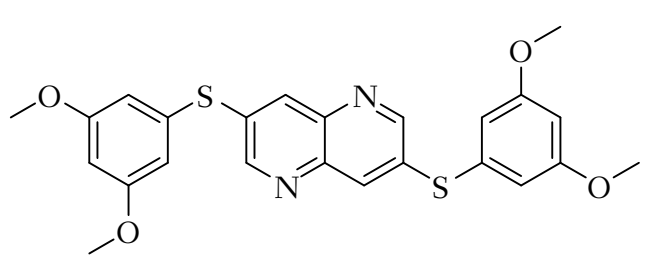

3,7-bis((3,5-Dimethoxyphenyl)thio)-1,5-naphthyridine (6f). The reaction was performed according to general procedure ***II*** employing naphthyridine **5b** (100 mg, 0.347 mmol, 1 eq.) and thiophenol **S2** (130 mg, 0.764 mmol, 2.2 eq.) to afford **6f** (153 mg, 0.328 mmol, 94%) as a yellow solid; R_f = 0.33 (*n*-pentane/EtOAc 7:1); m.p.: 149-150 °C; ^{1}H NMR (400 MHz, $CDCl_3$) δ 8.76 (d, *J* = 2.2 Hz, 2H), 8.10 (d, *J* = 2.2 Hz, 2H), 6.62 (d, *J* = 2.3 Hz, 2H), 6.46 (t, *J* = 2.3 Hz, 2H), 3.76 (s, 12H); ^{13}C NMR (101 MHz, $CDCl_3$) δ 161.7, 152.0, 141.8, 135.5, 135.4, 133.5, 110.8, 101.3, 55.6; HRMS (ESI+) m/z calcd. for $C_{24}H_{22}N_2O_4S_2$: 467.1099 [M+H]$^+$; found 467.1113.

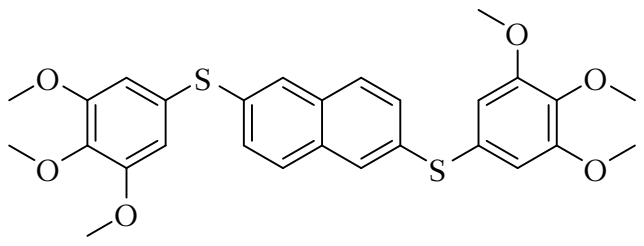

2,6-bis((3,4,5-Trimethoxyphenyl)thio)naphthalene (6g). The reaction was performed according to general procedure ***I*** employing naphthalene **5a** (150 mg, 0.395 mmol, 1 eq.) and 3,4,5-trimethoxybenzenethiol (174 mg, 0.869 mmol, 2.2 eq.). Purification by flash column chromatography (*n*-pentane/EtOAc 9:1 to 3:1) afforded **6g** (61 mg, 0.116 mmol, 30%) as a pale yellow solid; m.p.: 129-131 °C; ^{1}H-NMR (400 MHz, $CDCl_3$) δ 7.66 (d, *J* = 2.1 Hz, 2H), 7.63 (d, *J* = 8.6 Hz, 2H), 7.35 (dd, *J* = 8.6, 2.1 Hz, 2H), 6.69 (s, 4H), 3.87 (s, 6H), 3.79 (s, 12H); ^{13}C-NMR (101 MHz, $CDCl_3$) δ 153.9, 138.2, 134.7, 132.5, 129.0, 128.3, 128.2, 127.7, 109.8, 61.1, 56.4.

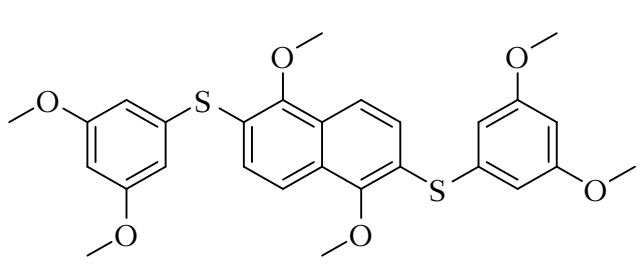

(1,5-Dimethoxynaphthalene-2,6-diyl)bis((3,5-dimethoxyphenyl)sulfane) (6h). The reaction was performed according to general procedure ***I*** employing naphthalene **5c** (240 mg, 0.545 mmol, 1 eq.) and thiophenol **S2** (204 mg, 1.2 mmol, 2.2 eq.). Purification by flash column chromatography (*n*-pentane/EtOAc 1:0 to 3:1) afforded **6h** (53 mg, 0.101 mmol, 19%) as a pale yellow solid; m.p.: 144-146 °C; ^{1}H-NMR (400 MHz, $CDCl_3$) δ 7.80 (d, *J* = 8.8 Hz, 2H), 7.30 (d, *J* = 8.8 Hz, 2H), 6.54 (d, *J* = 2.3 Hz, 4H), 6.38 (t, *J* = 2.3 Hz, 2H), 4.0 (s, 6H), 3.75 (s, 12H); ^{13}C-NMR (101 MHz, $CDCl_3$) δ 161.3, 155.0, 136.8, 129.9, 129.2, 125.0, 119.0, 109.1, 99.8, 61.9, 55.6.

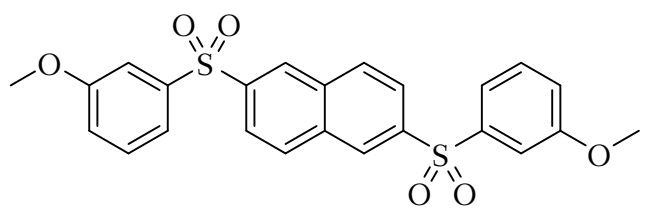

2,6-bis((3-methoxyphenyl)sulfonyl)naphthalene (7a). The reaction was performed according to general procedure ***III*** employing thioether **6a** (140 mg, 0.346 mmol, 1 eq.) and *m*CPBA (465 mg, 2.08 mmol, 6 eq.) in chloroform (0.03 M) for 20 h to afford the crude which was purified by trituration in Et_2O to yield **7a** (98 mg, 0.209 mmol, 60%) as a white solid; R_f = 0.25 (DCM/MeOH 19:1); m.p.: 230-232 °C; ^{1}H NMR (400 MHz, $CDCl_3$) δ 8.58 (d, *J* = 1.7 Hz, 2H), 8.07 (d, *J* = 8.6 Hz, 2H), 7.97 (dd, *J* = 8.6, 1.7 Hz, 2H), 7.55-7.53 (m, 2H), 7.49-7.47 (m, 2H), 7.41 (t, *J* = 8.0 Hz, 2H), 7.08 (ddd, *J* = 8.0, 2.6, 1.0 Hz, 2H), 3.83 (s, 6H); ^{13}C NMR (101 MHz, $CDCl_3$) δ 160.3, 142.1, 141.8, 134.1, 131.2, 130.7, 128.7, 124.6, 120.2, 120.0, 112.6, 55.9; HRMS (ESI+) m/z calcd. for $C_{24}H_{20}O_6S_2$: 469.0779 $[M+H]^+$; found 469.0780.

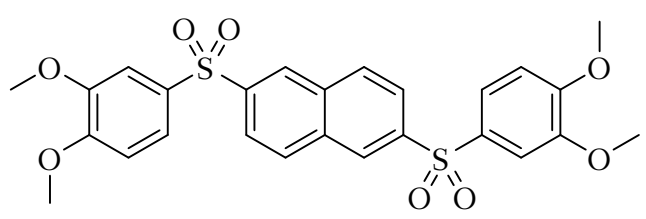

2,6-bis((3,4-dimethoxyphenyl)sulfonyl)naphthalene (7b). The reaction was performed according to general procedure ***III*** employing thioether **6b** (38 mg, 0.082 mmol, 1 eq.) and *m*CPBA (110 mg, 0.491 mmol, 6 eq.) in chloroform (0.03 M mL) for 20 h to afford the crude which was purified by flash column chromatography (MeOH/DCM 100:0 to 98:2) to yield **7b** (30 mg, 0.057 mmol, 69%) as a pale yellow solid; R_f = 0.22 (DCM/MeOH 19:1); m.p.: 245-247 °C; ^{1}H NMR (400 MHz, $CDCl_3$) δ 8.54 (d, *J* = 1.7 Hz, 2H), 8.05 (d, *J* = 8.7 Hz, 2H), 7.93 (dd, *J* = 8.7, 1.7 Hz, 2H), 7.60 (dd, *J* = 8.5, 2.1 Hz, 2H), 7.40 (d, *J* = 2.1 Hz, 2H), 6.93 (d, *J* = 8.5 Hz, 2H), 3.90 (s, 6H), 3.89 (s, 6H); ^{13}C NMR (101 MHz, $CDCl_3$) δ 153.5, 149.6, 142.3, 133.9, 132.3, 131.1, 128.1, 124.4, 122.4, 111.1, 110.1, 56.4, 56.4; HRMS (ESI+) m/z calcd. for $C_{26}H_{24}O_8S_2$: 529.0991 $[M+H]^+$; found 529.0974.

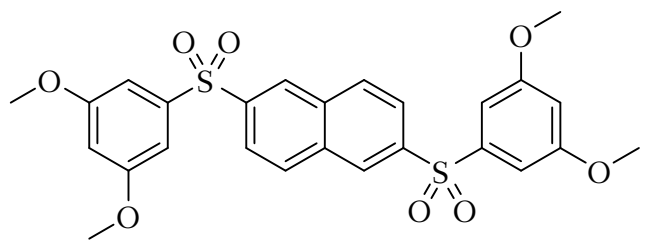

2,6-bis((3,5-dimethoxyphenyl)sulfonyl)naphthalene (7c). The reaction was performed according to general procedure ***III*** employing thioether **6c** (103 mg, 0.222 mmol, 1 eq.) and *m*CPBA (298 mg, 1.33 mmol, 6 eq.) in chloroform (0.03 M) for 20 h to afford the crude which was purified by flash column chromatography (MeOH/DCM 100:0 to 99:1) to yield **7c** (87 mg, 0.165 mmol, 74%) as a white solid; R_f = 0.21 (DCM/MeOH 19:1); m.p.: 270-272 °C; ^{1}H NMR (400 MHz, $CDCl_3$) δ 8.57 (d, *J* = 1.8 Hz, 2H), 8.07 (d, *J* = 8.6 Hz, 2H), 7.97 (dd, *J* = 8.6, 1.8 Hz, 2H), 7.08 (d, *J* = 2.3 Hz, 4H), 6.59 (t, *J* = 2.3 Hz, 2H), 3.80 (s, 12H); ^{13}C NMR (101 MHz, $CDCl_3$) δ 161.5, 142.6, 141.7, 134.1, 131.2, 128.7, 124.6, 105.9, 105.7, 56.0; HRMS (ESI+) m/z calcd. for $C_{26}H_{24}O_8S_2$: 529.0991 $[M+H]^+$; found 529.1000.

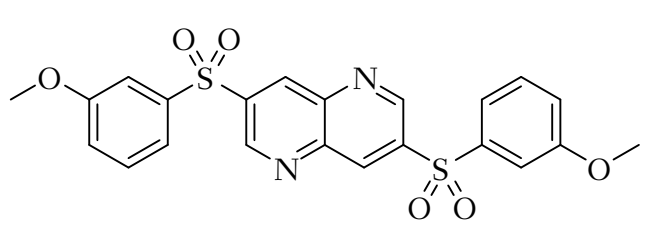

3,7-bis((3-methoxyphenyl)sulfonyl)-1,5-naphthyridine (7d). The reaction was performed according to general procedure ***III*** employing thioether **6d** (120 mg, 0.295 mmol, 1 eq.) and *m*CPBA (331 mg, 1.48 mmol, 5 eq.) in chloroform (0.05 M) for 24 h to afford **7d** (127 mg, 0.269 mmol, 91%) as a white solid. The product was used in the next step without further purification; R_f = 0.13 (DCM/MeOH 19:1); m.p.: 340-342 °C; ^{1}H NMR (400 MHz, $[D_6]$DMSO) δ 9.62 (d, *J* = 2.6 Hz, 2H), 9.13 (d, *J* = 2.6 Hz, 2H), 7.72 (ddd, *J* = 8.0, 1.7, 0.9 Hz, 2H), 7.65 (dd, *J* = 2.5, 1.7 Hz, 2H), 7.57 (t, *J* = 8.0 Hz, 2H), 7.29 (ddd, *J* = 8.0, 2.5, 0.9 Hz, 2H), 3.84 (s, 6H); ^{13}C NMR (101 MHz, $[D_6]$DMSO) δ 159.9, 149.5, 143.7, 140.7, 139.5, 137.4, 131.3, 120.9, 120.2, 112.5, 55.9; HRMS (ESI+) m/z calcd. for $C_{22}H_{20}N_2O_6S_2$: 471.0685 $[M+H]^+$; found 471.0703.

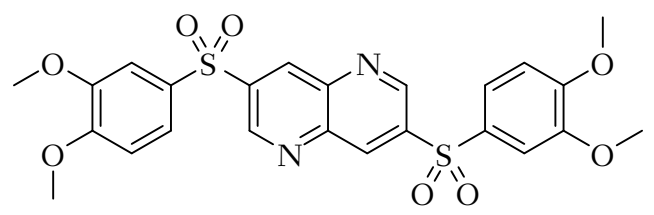

3,7-bis((3,4-dimethoxyphenyl)sulfonyl)-1,5-naphthyridine (7e). The reaction was performed according to general procedure ***III*** employing thioether **6e** (65 mg, 0.139 mmol, 1 eq.) and *m*CPBA (187 mg, 0.836 mmol, 6 eq.) in chloroform (0.05 M) for 24 h to afford **7e** (70 mg, 0.132 mmol, 95%) as a white solid. The product was used in the next step without further purification; R_f = 0.12 (DCM/MeOH 19:1); m.p.: 339-342 °C; ^{1}H NMR (400 MHz, $CDCl_3$) δ 9.43 (d, *J* = 2.5 Hz, 2H), 8.94 (d, *J* = 2.5 Hz, 2H), 7.68 (dd, *J* = 8.5, 2.2 Hz, 2H), 7.42 (d, *J* = 2.2 Hz, 2H), 6.97 (d, *J* = 8.6 Hz, 2H), 3.92 (s, 12H); HRMS (ESI+) m/z calcd. for $C_{24}H_{22}N_2O_8S_2$: 531.0896 $[M+H]^+$; found 531.0895.

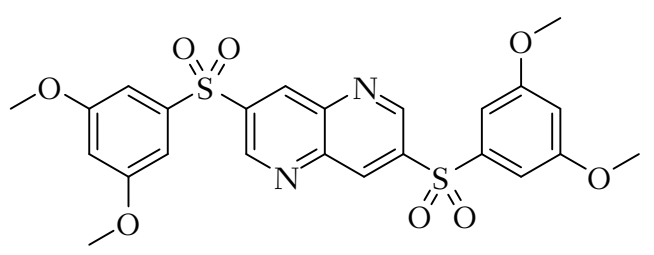

3,7-bis((3,5-dimethoxyphenyl)sulfonyl)-1,5-naphthyridine (7f). The reaction was performed according to general procedure ***III*** employing thioether **6f** (100 mg, 0.214 mmol, 1 eq.) and *m*CPBA (240 mg, 1.07 mmol, 5 eq.) in chloroform (0.05 M) for 24 h to afford **7f** (90 mg, 0.169 mmol, 79%) as a yellow solid. The product was highly insoluble in DCM, EtOAc, MeCN, MeOH, acetone, toluene, Et_2O, THF and H_2O. It was used in the next step without further purification; R_f = 0.13 (DCM/MeOH 19:1); m.p.: 364-367 °C; ^{1}H NMR (400 MHz, $CDCl_3$) δ 9.45 (d, *J* = 2.5 Hz, 2H), 8.98 (d, *J* = 2.6 Hz, 2H), 7.12 (d, *J* = 2.3 Hz, 4H), 6.64 (t, *J* = 2.2 Hz, 2H), 3.82 (s, 12H); HRMS (ESI+) m/z calcd. for $C_{24}H_{22}N_2O_8S_2$: 531.0896 $[M+H]^+$; found 531.0894.

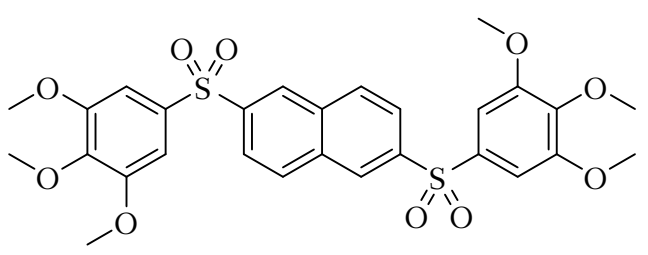

2,6-bis((3,4,5-Trimethoxyphenyl)sulfonyl)naphthalene (7g). The reaction was performed according to general procedure ***III*** employing thioether **6g** (38 mg, 0.072 mmol, 1 eq.) and *m*CPBA (97 mg, 0.435 mmol, 6 eq.) in chloroform (0.05 M) for 20 h to afford **7g** (39 mg, 0.066 mmol, 92%) as a white solid. The product was used in the next step without further purification; m.p.: 289-290 °C; ^{1}H-NMR (400 MHz, DMSO-d_6) δ 8.85 (d, *J* = 1.9 Hz, 2H), 8.39 (d, *J* = 8.7 Hz, 2H), 8.16 (dd, *J* = 8.7, 1.9 Hz, 2H), 7.27 (s, 4H), 3.85 (s, 12H), 3.68 (s, 6H); ^{13}C-NMR (101 MHz, DMSO-d_6) δ 153.3, 141.7, 141.3, 135.1, 133.6, 131.6, 128.4, 124.3, 105.1, 60.2, 56.4.

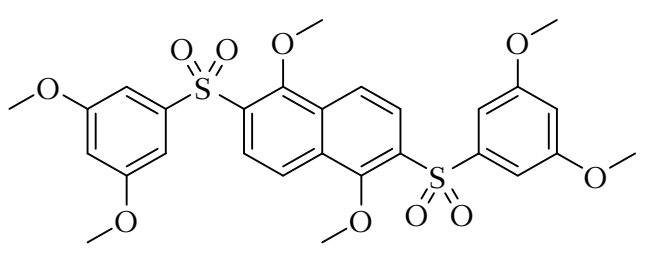

2,6-bis((3,5-Dimethoxyphenyl)sulfonyl)-1,5-dimethoxynaphth-alene (7h). The reaction was performed according to general procedure ***III*** employing thioether **6h** (45 mg, 0.086 mmol, 1 eq.) and *m*CPBA (115 mg, 0.515 mmol, 6 eq.) in chloroform (0.05 M) for 16 h to afford **7h** (33 mg, 0.056 mmol, 65%) as a white solid after purification by preparative TLC ($CHCl_3$/MeOH 9:1); m.p.: 238-240 °C; ^{1}H-NMR (400 MHz, $CDCl_3$) δ 8.17 (d, *J* = 8.8 Hz, 2H), 7.99 (d, *J* = 8.8 Hz, 2H), 7.11 (d, *J* = 2.3 Hz, 4H), 6.60 (t, *J* = 2.3 Hz, 2H), 4.14 (s, 6H), 3.80 (s, 12H); ^{13}C-NMR (101 MHz, $CDCl_3$) δ 160.9, 156.9, 143.4, 133.6, 133.0, 125.5, 120.2, 105.9, 65.1, 55.9. Two aromatic CH signals overlap, see HSQC (C at 105.9 corresponds to the H signals at 7.11 and 6.60).

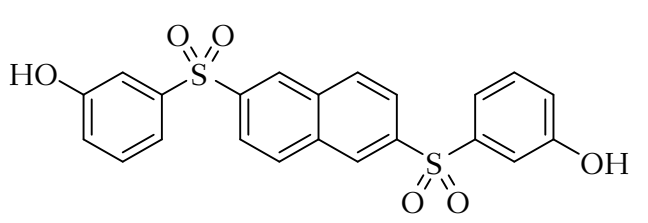

3,3'-(naphthalene-2,6-disulfonyl)diphenol (8a). The reaction was performed according to general procedure ***IV*** employing sulfone **7a** (50 mg, 0.107 mmol, 1 eq.) and BBr_3 (0.43 mL, 0.43 mmol, 4 eq.) in DCM (0.04 M) for 16 h to afford **8a** (45 mg, 0.102 mmol, 96%) as a white solid after trituration with Et_2O; R_f = 0.25 (DCM/MeOH 9:1); m.p.: 323-325 °C; ^{1}H NMR (400 MHz, [D_6]DMSO) δ 10.27 (s, 2H), 8.80 (d, *J* = 1.8 Hz, 2H), 8.44 (d, *J* = 8.7 Hz, 2H), 8.02 (dd, *J* = 8.7, 1.8 Hz, 2H), 7.42-7.41 (m, 4H), 7.32 (m, 2H), 7.06-7.03 (m, 2H); ^{13}C NMR (101 MHz,

$[D_6]$DMSO) δ 158.1, 141.5, 141.1, 133.6, 131.7, 131.1, 128.5, 124.2, 121.0, 118.0, 113.6; HRMS (ESI+) m/z calcd. for $C_{22}H_{17}O_6S_2$: 441.0388 $[M + H]^+$; found 441.0403.

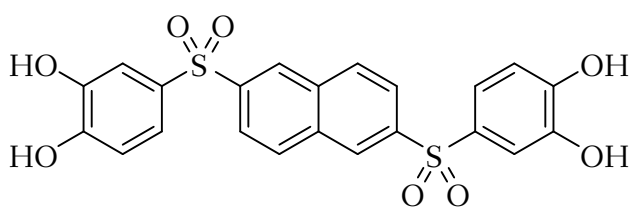

4,4'-(naphthalene-2,6-disulfonyl)bis(benzene-1,2-diol) (8b). The reaction was performed according to general procedure ***IV*** employing sulfone **7b** (28 mg, 0.053 mmol, 1 eq.) and BBr_3 (0.42 mL, 0.42 mmol, 8 eq.) in DCM (0.04 M) for 24 h to afford **8b** (18 mg, 0.034 mmol, 72%) as a beige solid after trituration with a mixture of DCM/Et_2O (1:4); R_f = 0.23 (DCM/MeOH 9:1); m.p.: >350 °C (decomp.); ^1H NMR (400 MHz, $[D_6]$DMSO) δ 10.18 (br s, 2H), 9.76 (br s, 2H), 8.68 (d, *J* = 1.8 Hz, 2H), 8.38 (d, *J* = 8.7 Hz, 2H), 7.94 (dd, *J* = 8.7, 1.8 Hz, 2H), 7.32 (dd, *J* = 8.4, 2.3 Hz, 2H), 7.28 (d, *J* = 2.3 Hz, 2H), 6.90 (d, *J* = 8.4 Hz, 2H); ^{13}C NMR (101 MHz, $[D_6]$DMSO) δ 150.9, 145.9, 142.0, 133.4, 131.4, 130.0, 127.5, 124.0, 120.4, 116.0, 114.3; HRMS (ESI+) m/z calcd. for $C_{22}H_{17}O_8S_2$: 473.0359 $[M + H]^+$; found 473.0344.

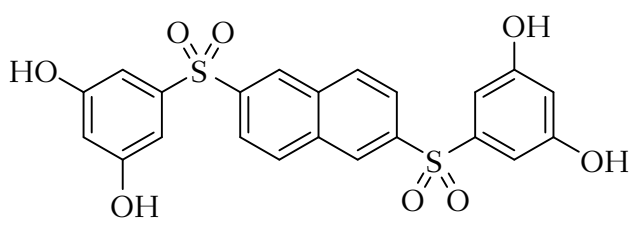

5,5'-(naphthalene-2,6-disulfonyl)bis(benzene-1,3-diol) (8c). The reaction was performed according to general procedure ***IV*** employing sulfone **7c** (42 mg, 0.079 mmol, 1 eq.) and BBr_3 (0.64 mL, 0.64 mmol, 8 eq.) in DCM (0.04 M) for 96 h to afford **8c** (32 mg, 0.068 mmol, 85%) as a white solid after trituration with a mixture of DCM/Et_2O (1:4) and with Et_2O; R_f = 0.23 (DCM/MeOH 9:1); m.p.: >350 °C (decomp.); ^1H NMR (400 MHz, $[D_6]$DMSO) δ 10.07 (br s, 4H), 8.76 (d, *J* = 1.8 Hz, 2H), 8.45 (d, *J* = 8.7 Hz, 2H), 7.98 (dd, *J* = 8.7, 1.8 Hz, 2H), 6.77 (d, *J* = 2.1 Hz, 4H), 6.43 (t, *J* = 2.1 Hz, 2H); ^{13}C NMR (101 MHz, $[D_6]$DMSO) δ 159.3, 142.0, 141.1, 133.6, 131.7, 128.3, 124.1, 107.4, 105.2; HRMS (ESI+) m/z calcd. for $C_{22}H_{17}O_8S_2$: 473.0359 $[M + H]^+$; found 473.0353.

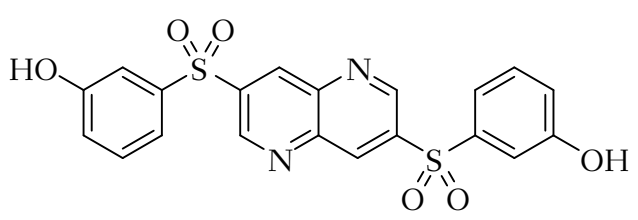

3,3'-(1,5-naphthyridine-3,7-disulfonyl)diphenol (8d). The reaction was performed according to general procedure ***IV*** employing sulfone **7d** (50 mg, 0.106 mmol, 1 eq.) and BBr_3 (2.12 mL, 2.12 mmol, 20 eq.) in DCM (0.05 M) for 48 h to afford **8d** (42 mg, 0.095 mmol, 89%) as a cream-coloured solid after trituration with MeOH; R_f = 0.15 (DCM/MeOH 9:1); m.p.: 340-342 °C; ^1H NMR (400 MHz, $[D_6]$DMSO) δ 10.34 (s, 2H), 9.55 (d, *J* = 2.6 Hz, 2H), 9.04 (d, *J* = 2.6 Hz, 2H), 7.57 (ddd, *J* = 7.9, 1.9, 1.0 Hz, 2H), 7.49 - 7.41 (m, 4H), 7.09 (ddd, *J* = 8.2, 2.5, 1.0 Hz, 2H); ^{13}C NMR (101 MHz, $[D_6]$DMSO) δ 158.3, 149.4, 143.7, 140.5, 139.6, 137.1, 131.3, 121.7, 118.5, 114.0; HRMS (ESI+) m/z calcd. for $C_{20}H_{14}N_2O_6S_2$: 443.0371 $[M+H]^+$; found 443.0366.

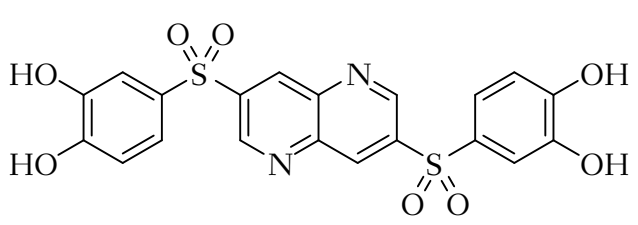

4,4'-(1,5-naphthyridine-3,7-disulfonyl)bis(benzene-1,2-diol) (8e). The reaction was performed according to general procedure ***IV*** employing sulfone **7e** (20 mg, 0.038 mmol, 1 eq.) and BBr_3 (0.75 mL, 0.75 mmol, 20 eq.) in DCM (0.05 M) for 96 h to afford **8e** (17 mg, 0.036 mmol, 95%) as a yellow solid after trituration with MeOH; R_f = 0.12 (DCM/MeOH 9:1); m.p.: 369-371 °C; ^1H NMR (400 MHz, $[D_6]$DMSO) δ 10.36 (s, 2H), 9.84 (s, 2H), 9.47 (d, *J* = 2.6 Hz, 2H), 8.90 (d, *J* = 2.6 Hz, 2H), 7.46 (dd, *J* = 8.4, 2.4 Hz, 2H), 7.38 (d, *J* = 2.4 Hz, 2H), 6.93 (d, *J* = 8.4 Hz, 2H); ^{13}C NMR (101 MHz, $[D_6]$DMSO) δ 151.7, 149.2, 146.1, 143.4, 140.5, 136.0, 128.9, 121.0, 116.2, 114.6; HRMS (ESI+) m/z calcd. for $C_{20}H_{14}N_2O_8S_2$: 475.0270 $[M+H]^+$; found 475.0271.

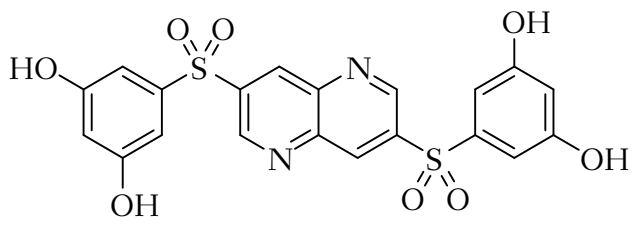

5,5′-(1,5-naphthyridine-3,7-disulfonyl)bis(benzene-1,3-diol) (8f). The reaction was performed according to general procedure ***IV*** employing sulfone **7f** (60 mg, 0.113 mmol, 1 eq.) and BBr_3 (2.26 mL, 2.26 mmol, 20 eq.) in DCM (0.05 M) for 48 h. Purification by preparative TLC (DCM/MeOH 9:1) afforded **8f** (8 mg, 0.017 mmol, 15%) as a yellow solid; R_f = 0.10 (DCM/MeOH 9:1); m.p.: 365-369 °C; ^{1}H NMR (400 MHz, [D_4]MeOH) δ 9.42 (d, *J* = 2.5 Hz, 2H), 8.97 (d, *J* = 2.5 Hz, 2H), 6.93 (d, *J* = 2.2 Hz, 4H), 6.49 (t, *J* = 2.2 Hz, 2H); ^{13}C NMR (101 MHz, [D_4]MeOH) δ 161.2, 150.7, 150.6, 145.1, 142.5, 142.0, 138.2, 138.2, 109.2, 107.0; HRMS (ESI+) m/z calcd. for $C_{20}H_{14}N_2O_8S_2$: 475.0270 $[M+H]^+$; found 475.0276.

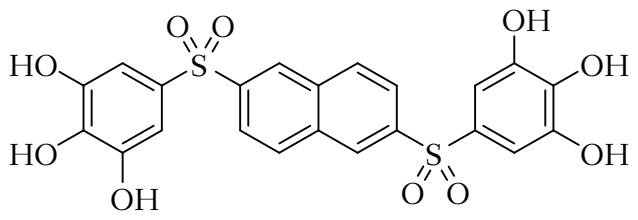

5,5′-(Naphthalene-2,6-disulfonyl)bis(benzene-1,2,3-triol) (8g). The reaction was performed according to general procedure ***IV*** employing sulfone **7g** (29 mg, 0.049 mmol, 1 eq.) and BBr_3 (0.59 mL, 0.59 mmol, 12 eq.) in DCM (0.05 M) for 24 h. Trituration of the crude with Et_2O (5x) and toluene (2x) afforded **8g** (8 mg, 0.016 mmol, 32%) as a beige solid; m.p.: >350 °C (decomp.); ^{1}H-NMR (400 MHz, CD_3OD) δ 8.52 (d, *J* = 1.7 Hz, 2H), 8.15 (d, *J* = 8.7 Hz, 2H), 7.90 (dd, *J* = 8.7, 1.7 Hz, 2H), 6.97 (s, 4H); ^{13}C-NMR (101 MHz, CD_3OD) δ 147.4, 143.9, 140.1, 135.1, 132.1, 131.0, 128.8, 125.2, 108.3; HRMS (ESI+) calcd. for $C_{22}H_{17}O_{10}S_2$: 505.0258 $[M+H]^+$; found 505.0255.

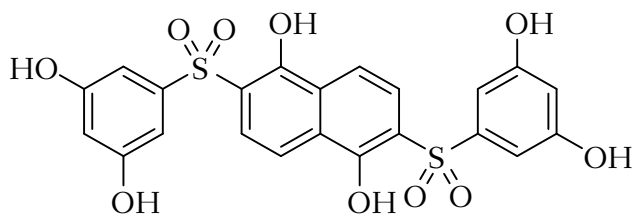

5,5′-(1,5-Dihydroxynaphthalene-2,6-disulfonyl)bis(benzene -1,3-diol) (8h). The reaction was performed according to general procedure ***IV*** employing sulfone **7h** (28 mg, 0.048 mmol, 1 eq.) and BBr_3 (0.57 mL, 0.57 mmol, 12 eq.) in DCM (0.05 M) for 144 h. Purification by preparative TLC (DCM/MeOH 95:5) afforded **8h** (13 mg, 0.026 mmol, 54%) as a pale brown solid; m.p.: >350 °C (decomp.); ^{1}H-NMR (400 MHz, CD_3OD) δ 7.85 (d, *J* = 9.0 Hz, 2H), 7.66 (d, *J* = 9.0 Hz, 2H), 6.86 (d, *J* = 2.2 Hz, 4H), 6.47 (t, *J* = 2.1 Hz, 2H); ^{13}C-NMR (101 MHz, CD_3OD) δ 160.8, 154.8, 144.1, 130.3, 125.2, 122.2, 116.3, 108.8, 106.2; HRMS (ESI-) calcd. for $C_{22}H_{15}O_8S_2$: 503.0112 $[M-H]^-$, found 503.0124.

*Synthesis of thiocarbamate **S1***

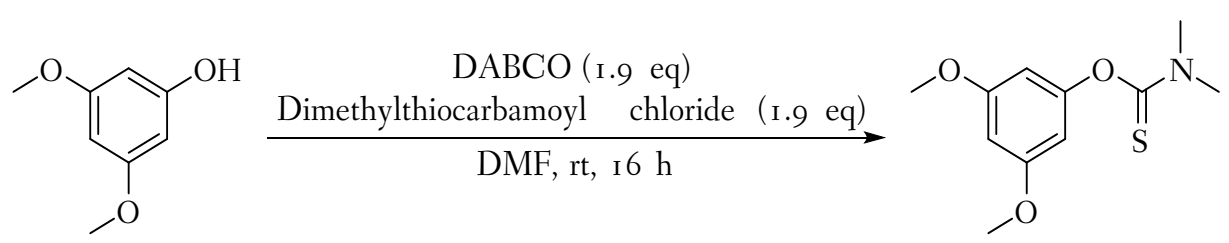

A solution of dimethylthiocarbamoyl chloride (10.66 g, 86.27 mmol, 1.9 eq.) in anhydrous DMF (20 mL) was slowly added to a 250 mL round-bottom flask charged with 3,5-dimethoxyphenol (7.00 g, 45.4 mmol, 1.0 eq.) and DABCO (9.68 g, 86.3 mmol, 1.9 eq.) in anhydrous DMF (45 mL). The solution was stirred at r.t. for 16 h until conversion as indicated by LC-MS. The reaction mixture was diluted with Et_2O (300 mL), washed with distilled H_2O (3 x 200 mL), dried over anhydrous Na_2SO_4, filtered and concentrated under reduced pressure to afford the crude as a yellow oil (10.45 g), which was mounted on silica (40 g) and purified by flash column chromatography (EtOAc/*n*-pentane 1:5) to afford the product as an off-white solid (9.84 g, 40.8 mmol, 90%); ^{1}H NMR (400 MHz, $CDCl_3$) δ 6.39-6.33 (m, 1H), 6.25 (dd, *J* = 2.2, 0.7 Hz, 2H), 3.77 (s, 6H), 3.45 (s, 3H), 3.32 (s, 3H); ^{1}H NMR agree with previously published spectra for this compound.[1]

Newman-Kwart synthesis of thiophenol ***S2***

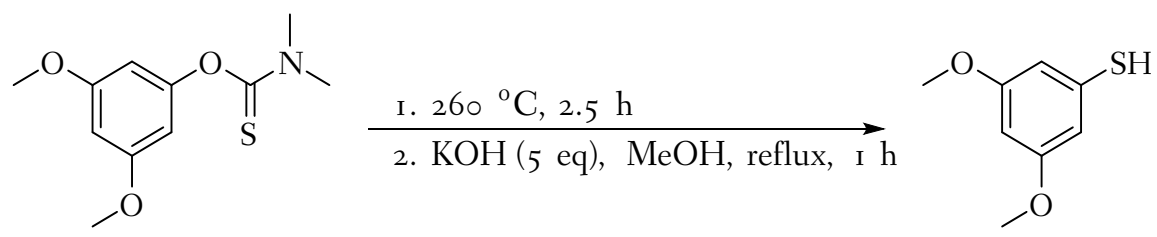

A 100 mL round-bottom flask was charged with **S1** (9.84 g, 40.8 mmol, 1 eq.), the flask was sealed, evacuated and backfilled with N_2 three times and the material was heated to 260 °C under N_2 atmosphere for 2.5 h. The reaction was monitored by ^{1}H NMR until complete conversion could be observed. The resulting brown-reddish oil was allowed to cool to r.t., MeOH (60 mL) and KOH (13.22 g, 203.9 mmol, 5 eq.) was added and the solution was heated at reflux for 1 h until complete hydrolysis of the S-thiocarbamate as indicated by TLC (EtOAc/*n*-pentane 1:9). The reaction mixture was cooled to r.t., concentrated under reduced pressure and the residue partitioned between EtOAc (200 mL) and HCl solution (2M, 120 mL). The organic fraction was isolated, washed with brine (100 mL), dried over anhydrous Na_2SO_4, filtered, concentrated under reduced pressure to afford the crude which was purified by normal phase column chromatography (EtOAc/*n*-pentane 1:4) to yield the product as a pale-yellow oil (5.21 g, 30.6 mmol, 75%); ^{1}H NMR (400 MHz, $CDCl_3$) δ 6.43 (d, *J* = 2.2 Hz, 2H), 6.26 (t, *J* = 2.2 Hz, 1H), 3.76 (s, 6H), 3.46 (s, 1H); ^{1}H NMR agree with previously published spectra for this compound.[1]

Supplementary figures and tables

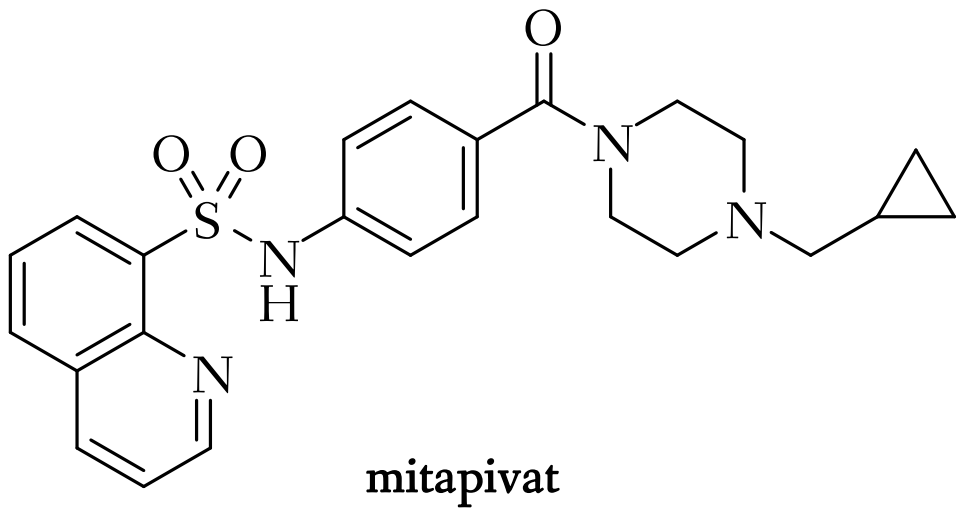

mitapivat

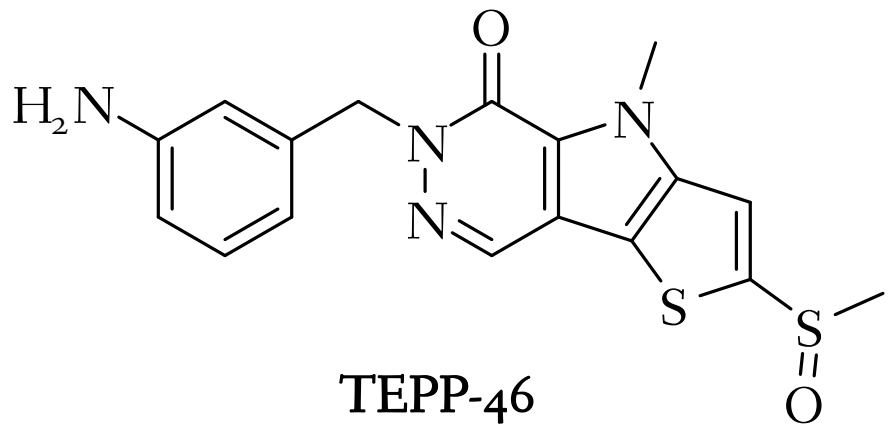

TEPP-46

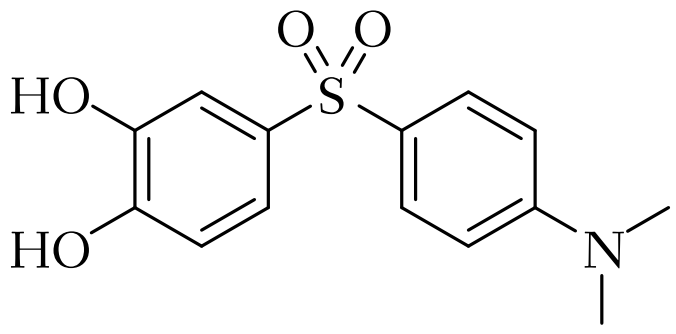

S3

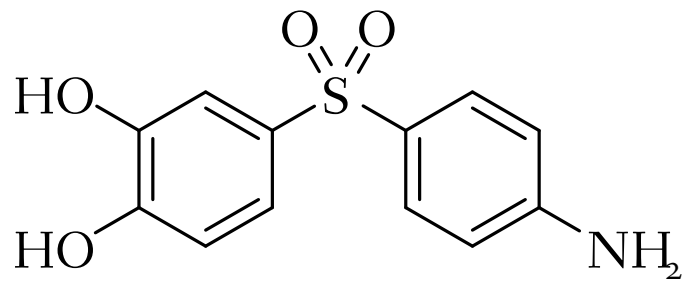

S4

Figure S1. Chemical structures of mitapivat, TEPP-46, **S3** and **S4**.

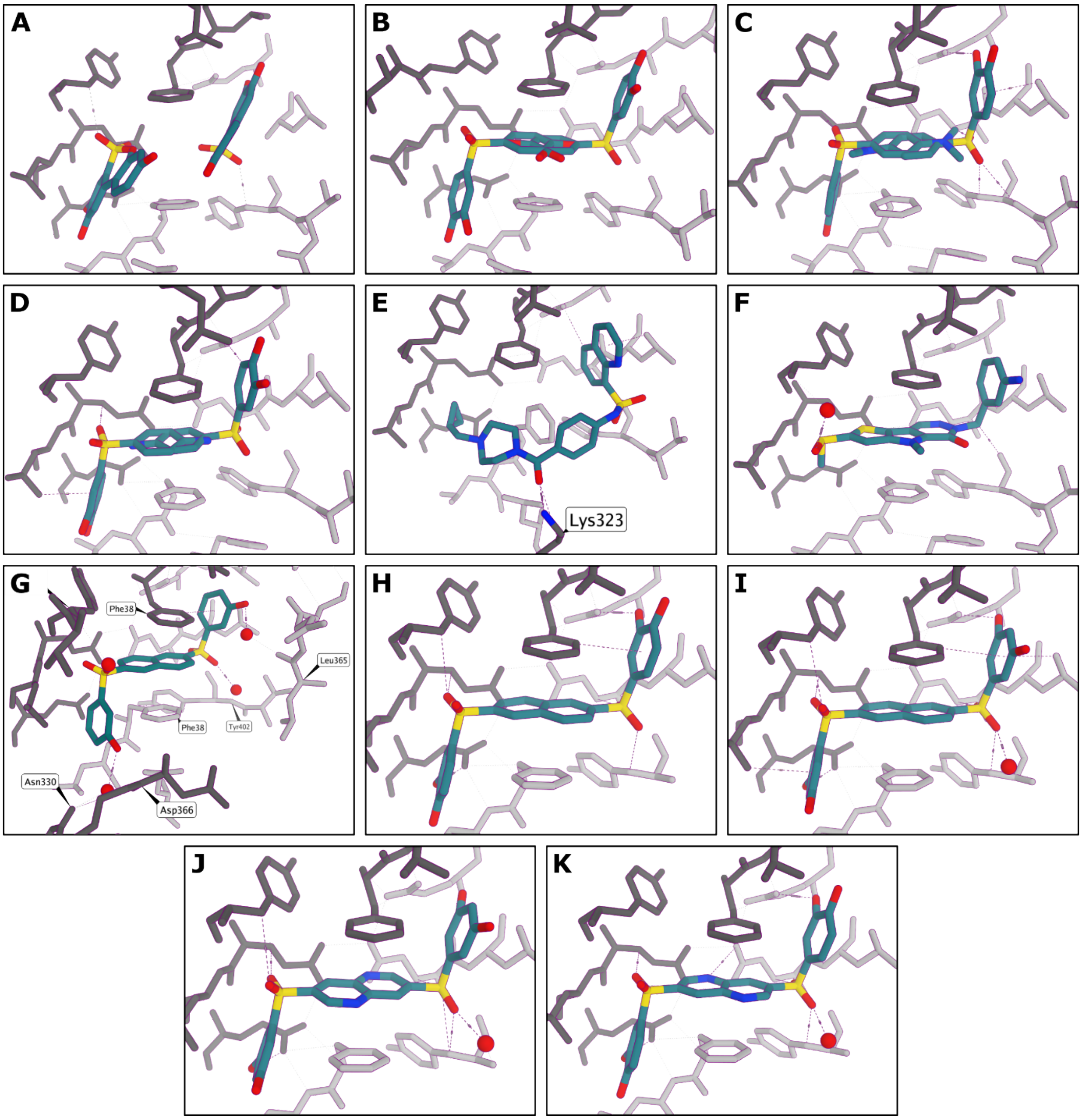

Figure S2. X-ray crystal structures of ligands in complex with PKL. Amino acid residues are coloured either light or dark grey to indicate the interface of PKL monomers; A. Compound **2** (PDB ID: 7FRW); B. Compound **3** (PDB ID: 7FRV); C. Compound **S3** (PDB ID: 14CM); D. Compound **S4** (PDB ID: 14CN); E. mitapivat (PDB ID: 14CK); F. TEPP-46 (PDB ID: 14CL); G. Compound **8a** (PDB ID: 14CO); H. Compound **8b** (PDB ID: 14CP); I. Compound **8c** (PDB ID: 14CQ); J. Compound **8f** (PDB ID: 14CT); Compound **8e** (PDB ID: 14CS)

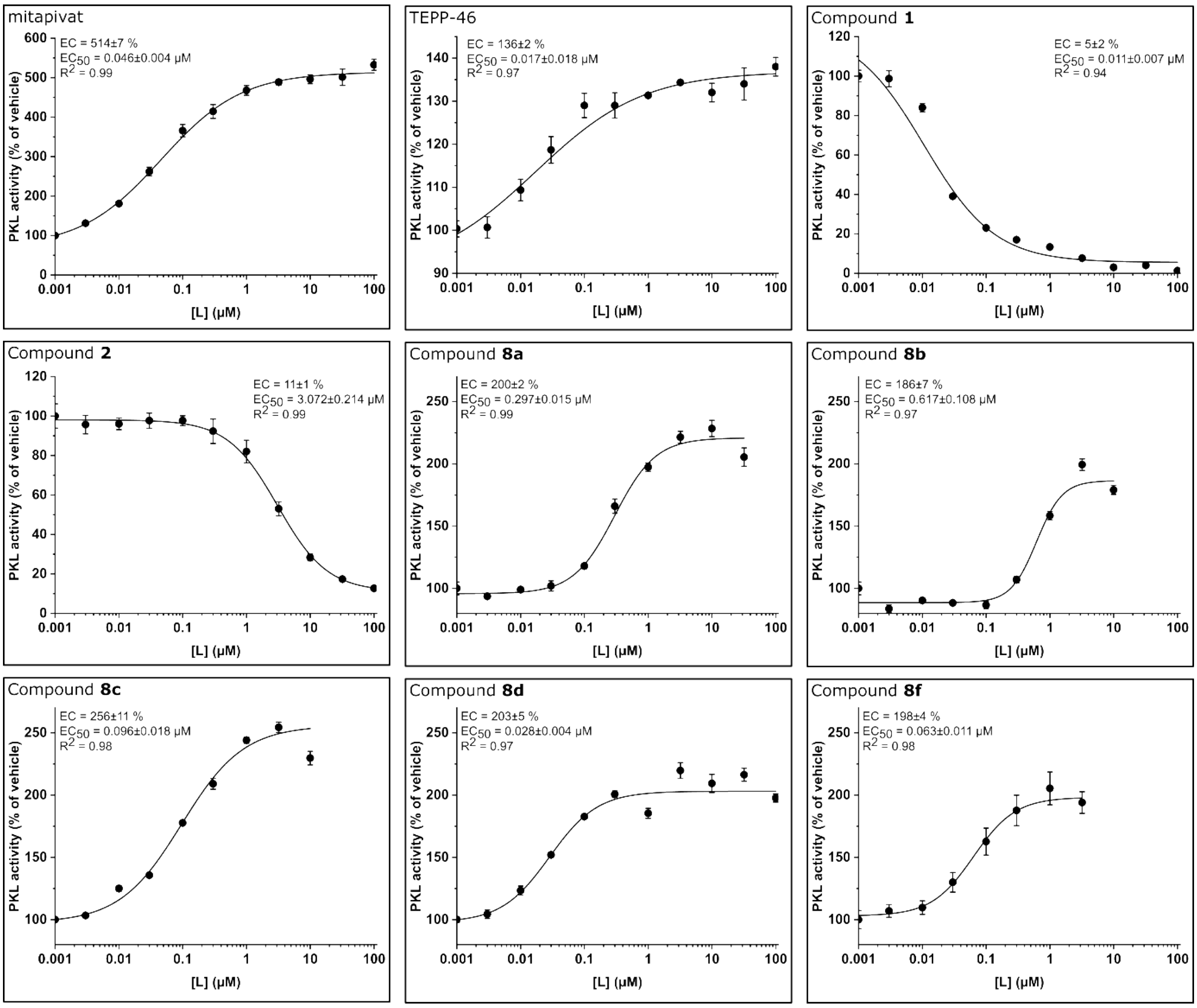

Figure S3. Dose-response curves of compounds incubated with recombinant PKL. Serially diluted solutions of the compounds were incubated with PKL (10 nM), PEP and (0.125 mM) ADP (0.1 mM) in TRIS-HCl buffer (50 mM Tris, pH 7.4, 10 mM $MgCl_2$, 100 mM KCl, 0.05% Tween) for 30 min before addition of Kinase-GLO detection reagent and luminescence quantification. Compounds were tested at concentrations between 0-100 μM, with the exception of compounds **8a** (0-32 μM), **8b** (0-10 μM) and **8f** (0-3.2 μM). The PKL activity is expressed as % of the vehicle (DMSO) control. Data is presented as Mean ± SD, n = 3, error bars show the SD.

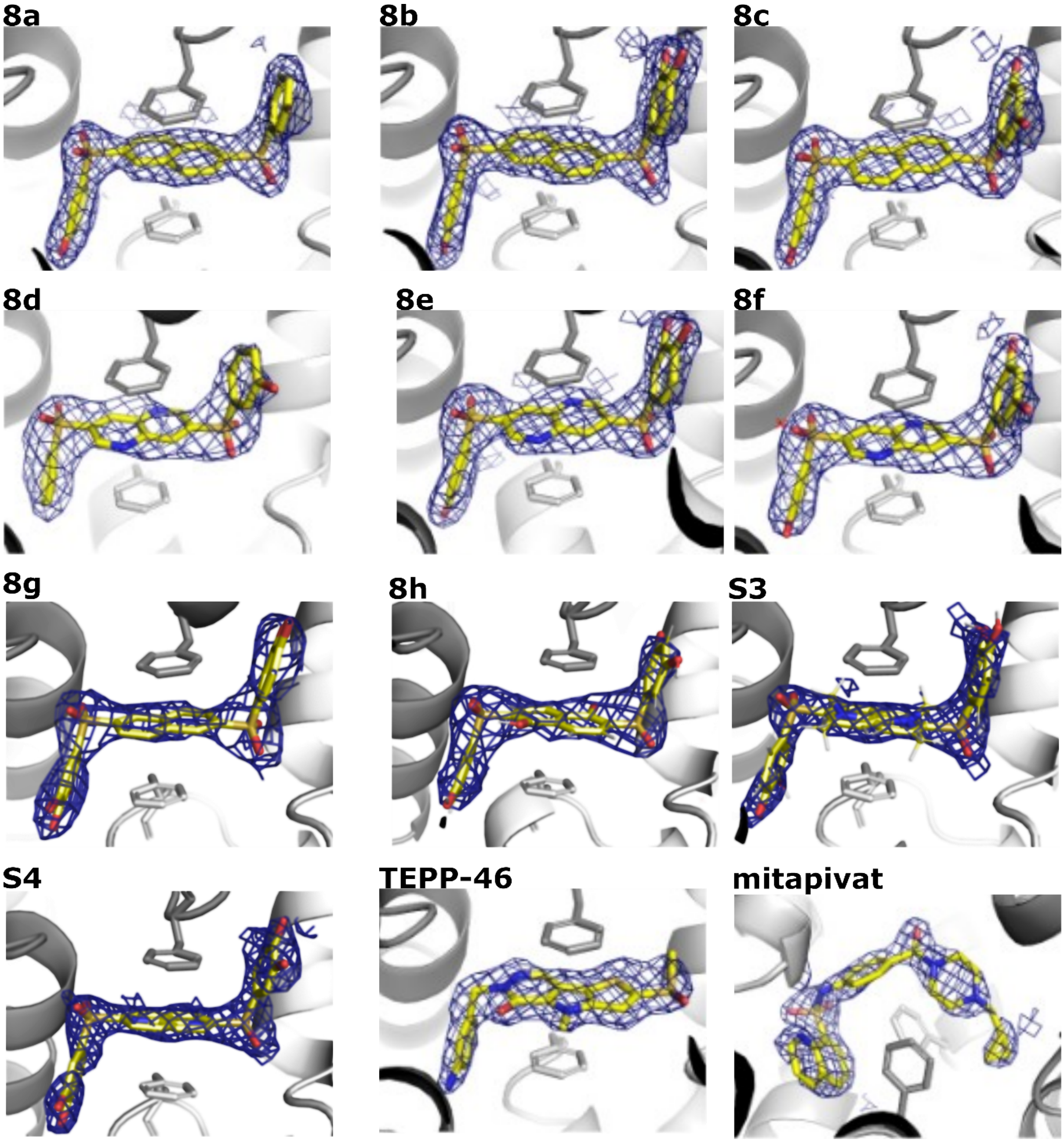

Figure S4. Electron density maps of PKL activators. 2mFo-DFc electron density maps for indicated ligands at the end of the refinement, sandwiched between two side chains of Phe38 (show as grey sticks) from neighbouring protomers in PKL. The densities in each case countered at 1 σ. PDB ID numbers: **8a** 14CO, **8b** 14CP, **8c** 14CQ, **8d** 14CR, **8e** 14CS, **8f** 14CT, **8g** 14CU, **8h** 14CV, **S3** 14CM, **S4** 14CN, TEPP-46 14CL, mitapivat 14CK.

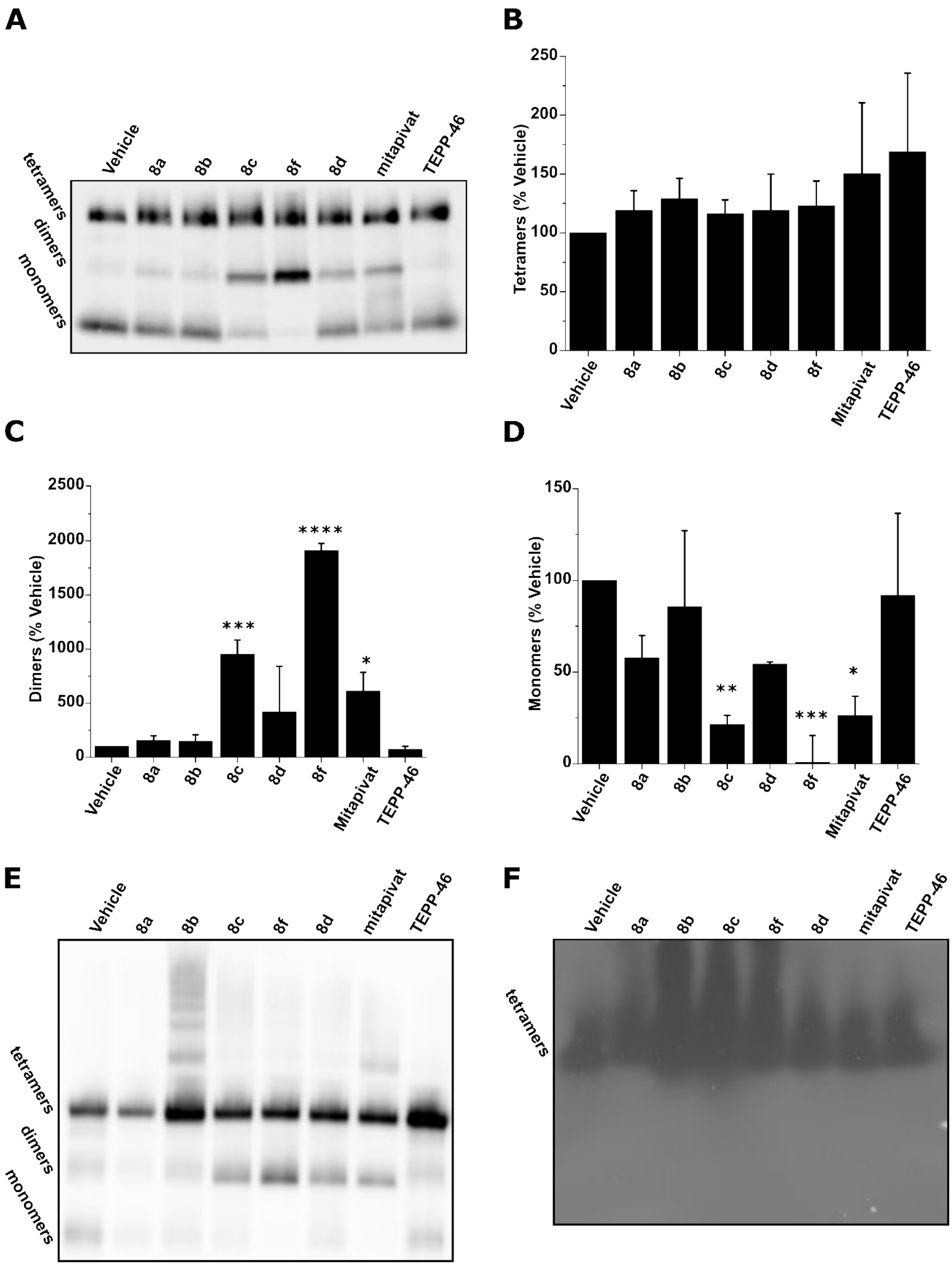

Figure S5. Native PAGE analysis of HepG2 PKM2 knockout cell lysates preincubated for 48 h at room temperature with 10 μM of compounds **8a-d** and **8f**, mitapivat, TEPP-46 or vehicle (DMSO) control; A. PKL oligomers as detected by immunoblotting with PKL-specific antibodies after separation of the proteins from compound-/vehicle-treated cells by Native PAGE electrophoresis; B., C., and D. Relative amounts of tetrameric, dimeric, and monomeric PKL, respectively, measured by quantification of three separate immunoblots (three biological replicates) performed as in C; E. PKL oligomers as detected by immunoblotting after preincubation of PKL recombinant (940 ng) for 30 min with 10 μM of compounds or vehicle; F. In-gel PKL activity after preincubation of PKL recombinant with the compounds as in G.; the dark areas indicate the PKL-catalysed formation of pyruvate; Mean ± SD, n = 3, * $p < 0.05$, ** $p < 0.01$ *** $p < 0.001$, **** $p < 0.0001$, One Way ANOVA followed by Dunnett's multiple comparison test.

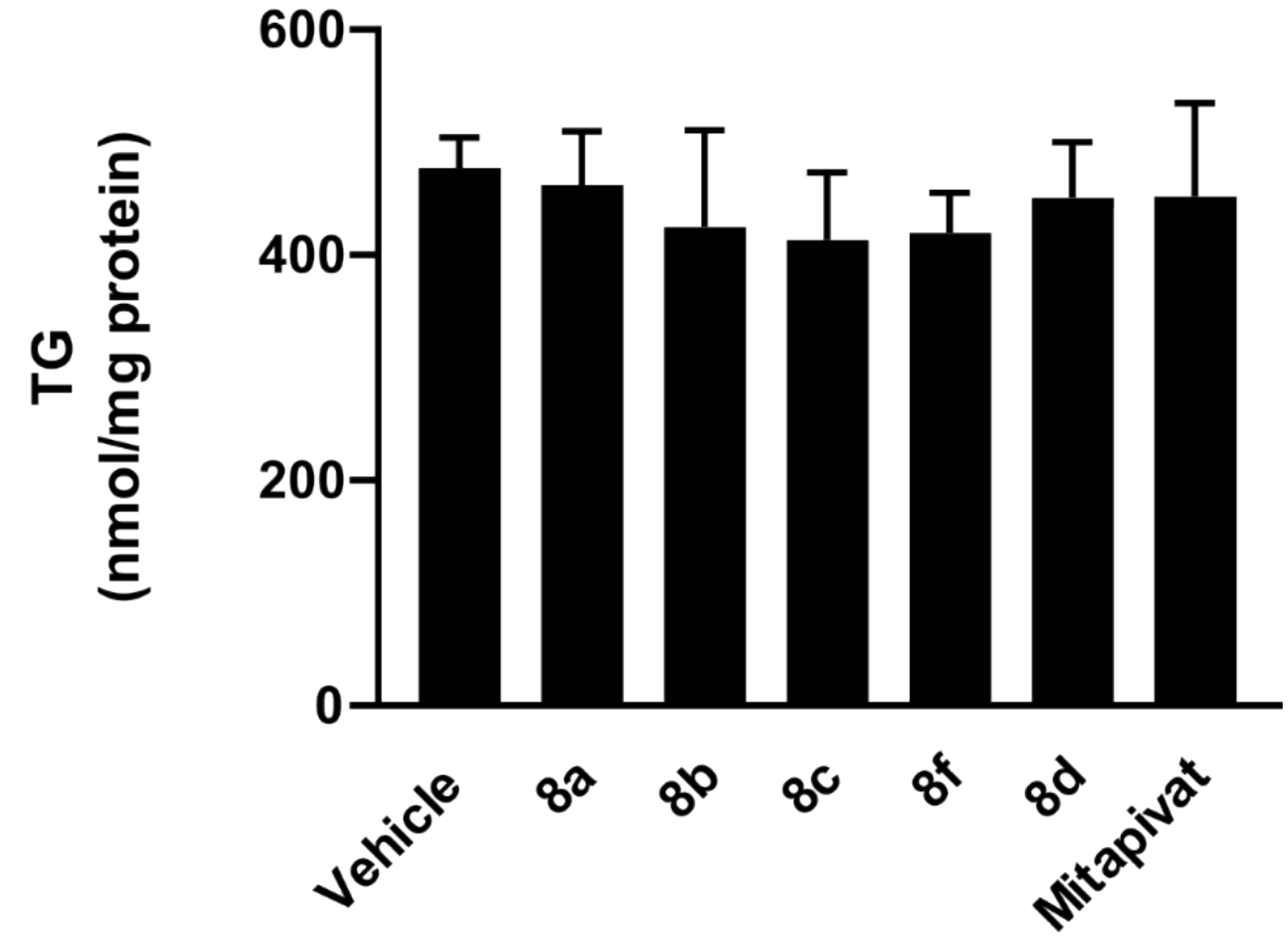

Figure S6. Lipidomics analysis of triglyceride (TG) content of HepG2 PKM2 knockout cells after treatment with 10 μM compounds or vehicle for 7 days in steatosis medium (DMEM containing 25 mM glucose, 10% FBS, 10 nM insulin and 10 μM T0901317). Data are from two independent experiments. Mean ± SD, n=3-9.

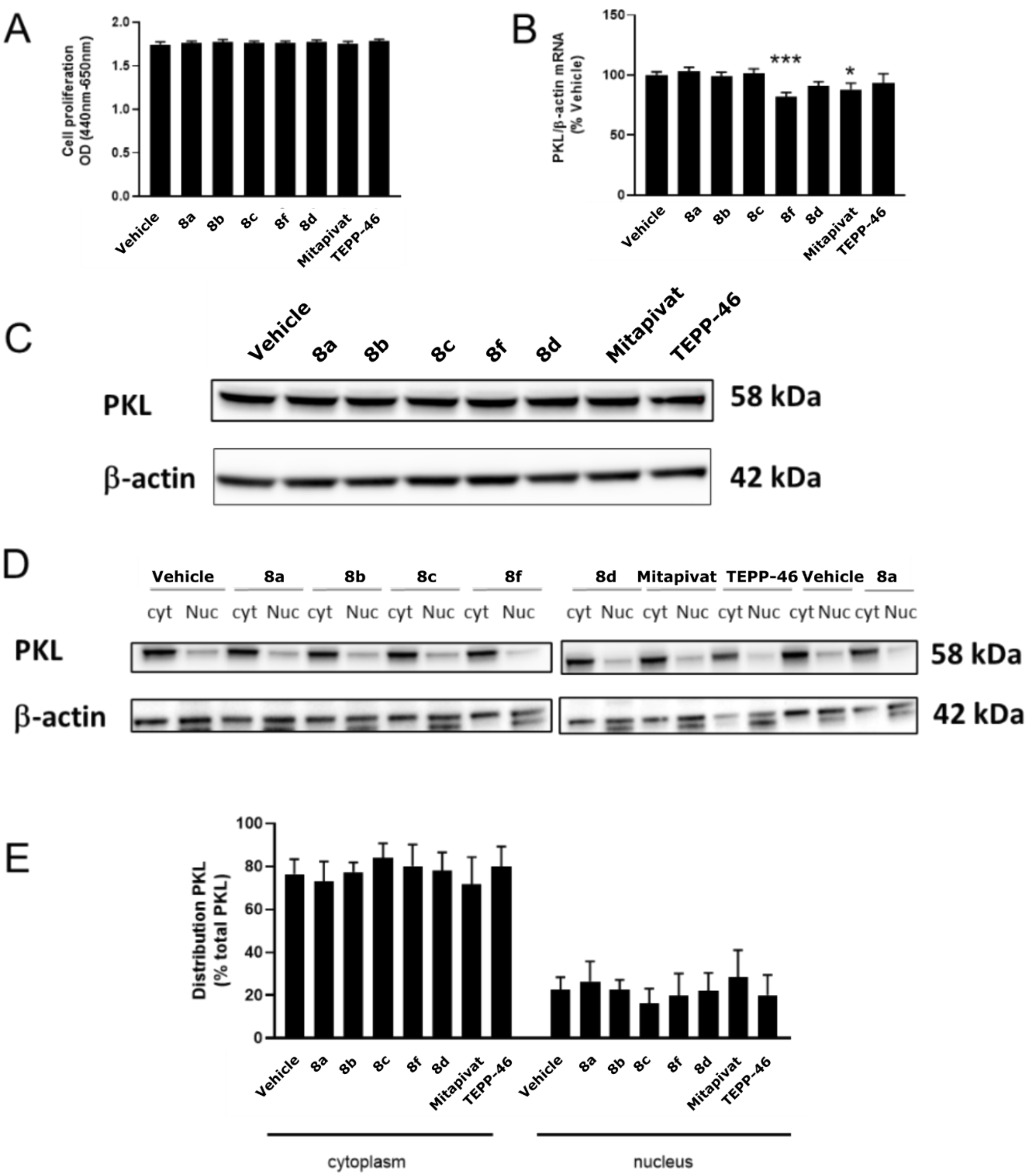

Figure S7. A. Cell proliferation after 72 h treatment of HepG2 PKM2 knockout cells with 10 μM compounds or vehicle. B. PKL mRNA expression after 2 h of treatment of HepG2 PKM2 knockout cells with 10 μM compounds or vehicle. C. PKL protein expression as assessed by immunoblotting after treatment for 48 h of HepG2 PKM2 knockout cells with 10 μM compounds. D. PKL distribution in the cytoplasm and nucleus as assessed by immunoblotting after treatment for 48 h of HepG2 PKM2 knockout cells with 10 μM compounds or vehicle. cyt= cytoplasm, Nuc= nucleus. E. Quantification of PKL distribution in the cytoplasm and nucleus after treatment for 48 h of HepG2 PKM2 knockout cells with 10 μM compounds (three independent experiments). Data are shown as mean ± SD, n=7-8 in A, n = 3 in A and B and n=4-12 in E, * $p < 0.05$, *** $p < 0.001$.

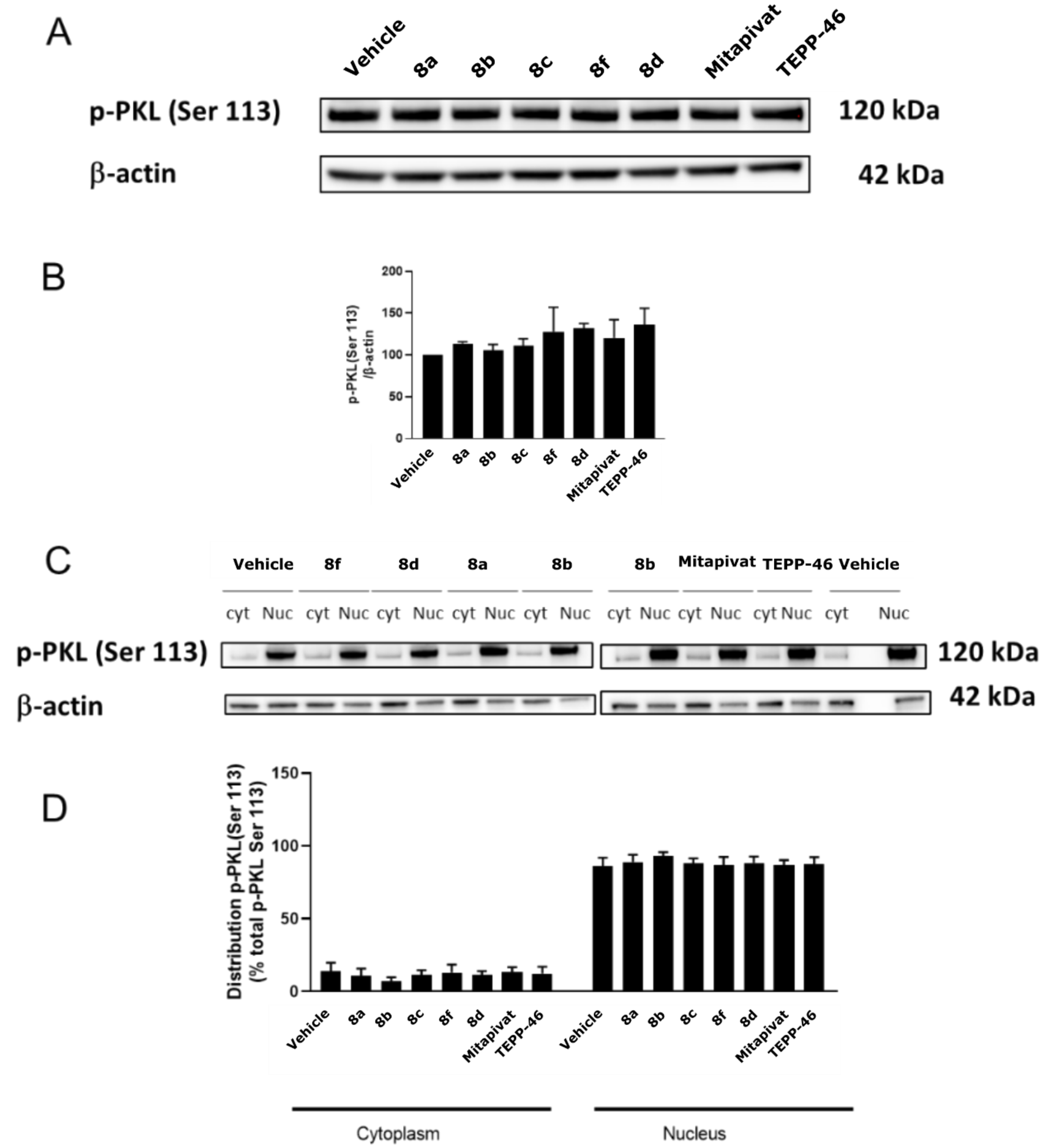

Figure S8. A. Phosphorylation of PKL at Ser113 as detected by immunoblotting after separation by SDS-PAGE of proteins from HepG2 PKM2 knockout cells treated for 48 h with 10 μM compounds or vehicle. B. Quantification of p-PKL (Ser 113) from three independent experiments performed as in A. C. Distribution of PKL phosphorylated at Ser 113 in the cytoplasm and nucleus as assessed by immunoblotting after treatment for 48 h of HepG2 PKM2 knockout cells with 10 μM compounds or vehicle. cyt=cytoplasm, Nuc=nucleus. D. Quantification of p-PKL (Ser 113) distribution in the cytoplasm and nucleus after treatment of HepG2 PKM2 knockout cells with 10 μM compounds for 48 h (three independent experiments). Data are shown as mean ± SD, n=3 in B; n=2-6 in D.

Table S1. Crystallographic data collection and refinement statistics. Data for the highest resolution bin are shown inside parentheses.

**Ligand**	**S3**	**S4**	**8a**
**PDB code**	**14CM**	**14CN**	**14CO**
**Data Collection and reduction**			
Wavelength [Å]	0.9763	0.9763	0.9795
Resolution range [Å]	104.23 - 1.93 (2.117 - 1.926)	188.71 - 1.99 (2.189 - 1.986)	103.65 - 1.89 (2.073 - 1.887)
Space group	C 1 2 1	C 1 2 1	C 1 2 1
Cell (a b c) [Å]	208.51 113.08 188.65	208.50 113.30 188.78	207.43 112.95 188.28
Cell (α β γ)	90.00 91.40 90.00	90.00 91.56 90.00	90.00 91.92 90.00
Total reflections	1602846 (78179)	1406857 (68137)	1727153 (90337)
Unique reflections	230651 (11534)	201342 (10068)	245505 (12276)
Multiplicity	6.9 (6.8)	7.0 (6.8)	7.0 (7.4)
Completeness (%)	88.5 (54.5)	87.9 (52.5)	94.0 (70.4)
Mean I/sigma(I)	13.0 (1.4)	14.5 (1.7)	10.1 (1.6)
R-merge	0.077 (1.23)	0.069 (1.14)	0.107 (1.25)
R-pim	0.031 (0.50)	0.028 (0.47)	0.043 (0.49)
CC-half	0.999 (0.65)	0.999 (0.66)	0.997 (0.59)
**Refinement**			
R-factor / Rfree	0.211 / 0.228	0.207 / 0.233	0.190 / 0.219
Number of total atoms	28069	28314	29110
atoms for ligands	368	384	372
atoms for waters	1548	1788	2425
Number of polymer residues	3394	3397	3410
Average B-factor [Å2]	44.4	53	40.1
B-factor for ligands [Å2]	40.5	49.6	35
B-factor for solvent [Å2]	48.6	55.4	50.6
RMS(bonds) [Å]	0.008	0.008	0.009
RMS(bond angles)[°]	0.91	0.91	0.92

**Ligand**	**8b**	**8c**	**8d**
**PDB code**	**14CP**	**14CQ**	**14CR**
**Data Collection and reduction**			
Wavelength [Å]	0.9795	0.9795	1.000
Resolution range [Å]	103.95 - 1.81 (2.013 - 1.808)	103.37 - 2.20 (2.387 - 2.201)	91.53 - 2.55 (2.758 - 2.550)
Space group	C 1 2 1	C 1 2 1	C 1 2 1
Cell (a b c) [Å]	207.99 112.54 188.84	206.92 112.69 187.89	203.09 112.22 184.65
Cell (α β γ)	90.00 91.65 90.00	90.00 92.39 90.00	90.00 94.06 90.00
Total reflections	1892933 (86813)	1042661 (54460)	563877 (32120)
Unique reflections	273378 (13670)	147817 (7391)	83629 (4182)
Multiplicity	6.9 (6.4)	7.1 (7.4)	6.7 (7.7)
Completeness (%)	95.5 (71.5)	94.3 (60.7)	89.8 (55.9)
Mean I/sigma(I)	11.0 (1.8)	7.9 (1.6)	5.1 (1.5)
R-merge	0.095 (1.02)	0.158 (1.34)	0.191 (1.21)
R-pim	0.039 (0.44)	0.064 (0.53)	0.080 (0.47)
CC-half	0.998 (0.66)	0.996 (0.53)	0.997 (0.80)
**Refinement**			
R-factor / Rfree	0.195 / 0.222	0.211 / 0.239	0.365 / 0.394
Number of total atoms	29274	28051	26841
atoms for ligands	356	352	344
atoms for waters	2642	1433	425
Number of polymer residues	3409	3405	3405
Average B-factor [Å2]	36.3 / -	42.5 / -	63.2
B-factor for ligands [Å2]	30.6	37.9	60.4
B-factor for solvent [Å2]	46.2	44.4	29.9
RMS(bonds) [Å]	0.009	0.008	0.007
RMS(bond angles)[°]	0.92	0.88	0.88

**Ligand**	**8e**	**8f**	**8g**
**PDB code**	**14CS**	**14CT**	**14CU**
**Data Collection and reduction**			
Wavelength [Å]	1.000	1.000	1.000
Resolution range [Å]	98.95 - 2.13 (2.375 - 2.129)	98.94 - 2.48 (2.721 - 2.484)	102.50 - 2.39 (2.640 - 2.387)
Space group	C 1 2 1	C 1 2 1	C 1 2 1
Cell (a b c) [Å]	206.78 112.73 187.78	205.76 112.90 185.80	205.32 112.66 186.40
Cell (α β γ)	90.00 92.70 90.00	90.00 93.41 90.00	90.00 93.18 90.00
Total reflections	970965 (49679)	1432587 (77996)	757220 (40075)
Unique reflections	137055 (6853)	103324 (5166)	105743 (5288)
Multiplicity	7.1 (7.2)	13.9 (15.1)	7.2 (7.6)
Completeness (%)	90.2 (67.4)	92.3 (56.0)	92.6 (79.4)
Mean I/sigma(I)	8.1 (1.7)	8.5 (1.6)	7.1 (1.7)
R-merge	0.233 (1.39)	0.191 (1.87)	0.156 (1.14)
R-pim	0.094 (0.55)	0.053 (0.50)	0.063 (0.44)
CC-half	0.992 (0.49)	0.999 (0.73)	0.997 (0.77)
**Refinement**			
R-factor / Rfree	0.212 / 0.252	0.256 / 0.282	0.255 / 0.289
Number of total atoms	27926	27146	27636
atoms for ligands	360	352	462
atoms for waters	1445	597	929
Number of polymer residues	3412	3401	3402
Average B-factor [Å2]	32.8	61.5	58.1
B-factor for ligands [Å2]	30.7	61.6	57
B-factor for solvent [Å2]	31.1	43	41.8
RMS(bonds) [Å]	0.009	0.007	0.007
RMS(bond angles)[°]	0.93	0.86	0.88

Ligand	8h	Mitapivat	TEPP-46
PDB code	14CV	14CK	14CL
**Data Collection and reduction**			
Wavelength [Å]	1.000	0.9763	1.000
Resolution range [Å]	98.34 - 3.07 (3.385 - 3.074)	99.30 - 1.96 (2.023 - 1.956)	103.59 - 2.13 (2.320 - 2.129)
Space group	C 1 2 1	C 1 2 1	C 1 2 1
Cell (a b c) [Å]	202.65 112.56 184.10	208.88 112.88 188.70	207.27 112.59 187.70
Cell (α β γ)	90.00 94.19 90.00	90.00 91.28 90.00	90.00 91.73 90.00
Total reflections	339339 (17633)	2773641 (148311)	2278606 (103898)
Unique reflections	48012 (2402)	271504 (13578)	166357 (8318)
Multiplicity	7.1 (7.3)	10.2 (10.9)	13.7 (12.5)
Completeness (%)	91.5 (79.1)	92.8 (91.8)	92.3 (54.4)
Mean I/sigma(I)	6.0 (1.6)	12.6 (1.5)	9.3 (1.5)
R-merge	0.160 (1.15)	0.107 (1.75)	0.222 (2.04)
R-pim	0.064 (0.46)	0.035 (0.55)	0.061 (0.58)
CC-half	0.997 (0.81)	0.999 (0.64)	0.997 (0.43)
**Refinement**			
R-factor / Rfree	0.290 / 0.307	0.199 / 0.224	0.218 / 0.249
Number of total atoms	26895	29066	27924
atoms for ligands	361	352	424
atoms for waters	290	2519	1234
Number of polymer residues	3402	3400	3412
Average B-factor [Å2]	82.4	42.1	45.4
B-factor for ligands [Å2]	81.1	45.8	41.6
B-factor for solvent [Å2]	19.6	49.2	46.3
RMS(bonds) [Å]	0.007	0.008	0.008
RMS(bond angles)[°]	0.88	0.9	0.91

Experimental procedures

Cell culture

HepG2 knockout for the PKM2 isoform (HepG2 PKM2 knockout cells) were obtained from Synthego (CA, USA). Cells were maintained in cell growing medium (RPMI 1640 medium supplemented with 2.5 mM glutamine, 10% FBS and 100 U/ml penicillin and 100 μg/ml streptomycin).

Determination of PKL activity after incubation of the compounds with cell lysates

For determination of PKL activity after incubation of cell lysates with the compounds, the HepG2 PKM2 knockout cells were plated in 10 cm dishes (3.6×10^6 cells/dish). Twenty-four hours later, the cells were washed 3 times with ice cold PBS and lysed with PK assay buffer (Pyruvate Kinase Assay Kit, Abcam). The cell lysates were centrifuged (4 ℃, 14000 x *g* at for 10 min). The protein concentration of the pooled cell lysate was determined using BCA kit (Pierce) and adjusted to 200 μg/ml with PK assay buffer. Cell lysates (10 μg total protein) were incubated for 5 min at r.t. with 10 μM compounds. The control cell lysates received DMSO as

vehicle. The PKL activity was determined using a PK assay kit (Abcam) according to manufacturer´s instructions.

Determination of PKL activity after treatment of the cells with the compounds

For determination of PKL activity after cell treatment with the compounds, the HepG2 PKM2 knockout cells were plated in 6 well plates ($2x10^5$ cells/well). Twenty-four hours later, the cells were treated for 48 h with 10 μM synthetic compounds or DMSO as vehicle in cell growing medium. The cells were washed, lysed with PK lysis buffer, cell lysates were centrifuged (4℃, 14000xg at for 10 min), protein concentration adjusted to 200 μg/ml and the PK activity of cell lysates (10 μg total protein) was determined as described above.

Measurement of oxygen consumption rate and glycolytic rate using Seahorse

The oxygen consumption rate (OCR) was measured with Mito stress test and the glycolytic proton efflux rate (glycoPER) was measured with glycolytic rate assay test using Seahorse XFe96 metabolic flux analyzer (Agilent). Cells were plated at a concentration of $7.5x10^4$ cells/well in seahorse cell plates and allow to adhere for 20 h at 37 °C in a 5% CO_2 incubator. The calibrator plate was equilibrated overnight in a non-CO_2 incubator. The cells were treated with 10 μM compounds or vehicle (DMSO) in growing medium and incubated for 4 h at 37 °C in 5% CO_2 incubator. Before the tests cells were washed twice with cell assay medium (unbuffered DMEM medium (Agilent) supplemented with 10 mM glucose and 2 mM glutamine, pH 7.4) and 10 μM compounds or vehicle (DMSO) in this medium were added again to the cells and incubated for 30 min in a non-CO_2-incubator. Ones the probe was calibrated the probe plate was replaced by the cell plate. Cellular OCR was measured at basal level and after sequential injection of mitochondrial respiratory chain inhibitors: 2 μM oligomycin, 2 μM FCCP, 1 μM antimycin plus 1 μM rotenone (Sigma). For glycolytic rate assay, the sequential injections were with 0.5 μM antimycin plus 0.5 μM rotenone and 50 mM 2-deoxyglucose (Sigma).

mRNA Gene expression

Total RNA was extracted using RNeasy Kit (QIAGEN) and cDNA was synthesized using the high capacity cDNA Reverse Transcription Kit (Applied Biosystems). mRNA expression of genes of interest was analyzed with Viia7 TaqMan real-time PCR (Applied Biosystems).

Cell proliferation

HepG2 PKM2 knockout cells were plated in a 96 well plate ($2x10^4$ cells/well). 24 hours later, the cells were treated for 72 h with the compounds at concentration of 10 μM in cell growing medium. The cell proliferation was determined using Quick cell proliferation assay kit II (Abcam) according to the manufacturer´s instructions.

PKL and p-PKL (Ser113) determination in cytoplasmic and nuclear fractions

Cells were plated in 6 well plates at cell density of 800 000 cells/well. Twenty-four hours later, the cells were treated with 10 μM compounds for 48 h. The vehicle control cells received DMSO. At the end of the incubation period, the cells were washed with PBS and the nucleus and cytoplasm were separated using the nuclear and cytoplasmic extraction kit from Pierce (cat78833) according to the manufacturer´s protocol. The protein concentration of the nuclear and cytoplasmic extracts was determined using BCA kit (Pierce), and the extracts were adjusted to the same protein concentration (687 μg/ml). Equal amounts of proteins (6.86 μg) were loaded to the 4-16% Bis-Tris gels (ThermoFisher Scientific), separated by SDS-PAGE and transferred

to nitrocellulose membranes. The membranes were incubated over night with primary antibodies (diluted 1:1000 in 5% milk in TBS-T), followed by secondary antibodies anti-rabbit or anti-mouse-HRP (diluted 1:5000 in 5% milk in TBS-T). The following primary antibodies were used: rabbit anti-PKLR (R&D Systems AF8519), rabbit anti-p-PKL (Ser113) (Millipore ABS2246) and mouse anti-actin (Abcam, ab 8826). The signals were developed using Supersignal-HRP substrate kit (Pierce). The images were acquired with a digital camera (Chemidoc, Biorad) and the chemiluminescent signal was quantified using Image J program.

Steatosis model and triglyceride determination

HepG2 PKM2 knockout cells were plated on 6 well plates at density $1x10^6$ cells/well in cell growing medium. 24 h later the compounds (10 μM) were added to the cells in steatosis medium (DMEM medium containing 25 mM glucose, no glutamine, no pyruvate supplemented with 10% FBS, 10 nM insulin and 10 μM T0901317). Cells were thereafter treated every second day with fresh steatosis medium containing the compounds or vehicle (DMSO) control. Seven days after addition of steatosis medium the cellular lipids were extracted, and the triglycerides were analyzed as previously described.[2] The results were normalized to the total protein concentration of the cell extract.

Gel-electrophoresis experiments

Determination of in-gel PKL activity

PKL recombinant obtained from Cambridge was incubated for 30 min on ice with 10 μM compounds. The samples were mixed with native sample buffer. The protein was separated by Native Clear PAGE electrophoresis (no Comassie G250 in the sample and cathode buffer) using Native PAGE 3-12% Bis-Tris gels (Invitrogen). The gels were run on ice with pre-cooled buffers.[3]

In gel PKL activity was determined according to (*Rivoal, J., Analytical Biochemistry 300, 94, 2002).*[4] Immediately after electrophoresis the gel was incubated at r.t. for 15 min in equilibration buffer (50 mM Mes-50 mM Bis-Tris propane pH 6.8, 1 mM DTT, 5% PEG 8000, 50 mM KCl and 10 mM $MgCl_2$). This solution was removed, and the gel was incubated for 5-10 min in development buffer (PK equilibration buffer plus 2 mM PEP, 1 mM ADP, 0.15 mM NADH and 2 U/ml rabbit muscle lactate dehydrogenase). The gel was visualized on a Chemidoc apparatus (Biorad) using a UV transiluminator. Activity bands (sites of NADH oxidation in gel) appear as dark bands after inverted image.

Native PAGE and immunoblotting for determination of PKL oligomerization

HepG2 PKM2 knockout cells were plated in 6 well plates at cell density of 800000 cells/well in growing medium. Twenty-four hours later, the compounds (10 μM) were added to the cells in triplicates in growing medium for 48 h. The control cells were treated with DMSO as vehicle. Cells were washed 3xPBS and lysed for 40 min in the 4C with lysis buffer containing 150 mM NaCl, 1% Triton X, 10 mM Hepes, 1 mM EDTA, phosphatase and protease inhibitors (Roche). The cell lysates were centrifuged at 4C (28,000xg for 10 min), and clear cell lysates adjusted to the same total protein concentration were mixed with native sample buffer and resolved under non-denaturating conditions by Native PAGE 3-12% Bis-Tris gels (10 μg protein/lane). The proteins were transferred on PVDF membranes. The membranes were blocked for 1 h with 5% milk in TBS-T and incubated overnight with PKL antibodies (AF8519, R&D systems) diluted 1:1000 in 5% milk in

TBS-T. The membranes were washed 3xTBS-T, incubated for 30 min at r.t. with secondary antibodies donkey anti-rabbit-HRP diluted in 5% milk in TBS-T and washed 3xTBS-T. The membranes were developed with Supersignal (Pierce). The images were visualized with a digital camera (Chemidoc, BioRAD) and the chemiluminescent signal was quantified using Image J program.

The PKL recombinant (930 ng) was incubated with the compounds (10 μM) for 30 min on ice. The protein was separated by Native PAGE electrophoresis followed by immunoblotting as described above.

Determination of PKL by luminescence assay

Biochemical assay measurements were outsourced to BPS Bioscience Inc. (6042 Cornerstone Court West, Ste. E, San Diego, CA 92121, USA), and performed according to the following procedure:

PKL reactions were conducted in triplicates at r.t. for 30 min in a 25 μl mixture containing 50 mM Tris, pH 7.4, 10 mM $MgCl_2$, 100 mM KCl, 0.05% Tween, 10 nM PKL, 0.1 mM ADP, 0.125 mM PEP, pyruvate kinase and the test compounds. The final DMSO in the reaction was 1%. After enzymatic reactions, 25 μl of Kinase-Glo Max reagent was added to each well and luminescence was measured using a BioTek Synergy 2 microplate reader (Promega). The Kinase-Glo Max luminescence assay kit measures PK activity by quantitating the amount of ATP produced following a PK reaction.

Enzyme activity assays were performed in triplicates at each concentration. The luminescence data were analyzed using the computer software Origin. In the absence of the compound, the intensity (C_e) in each data set was defined as 100% activity. In the absence of enzyme, the intensity (C_o) in each data set was defined as 0% activity. The percent activity in the presence of each compound was calculated according to the following equation: % activity = $(C-C_o)/(C_e-C_o)$, where C = the luminescence in the presence of the compound.

The values of % activity versus a series of compound concentrations were plotted using non-linear regression analysis of Sigmoidal dose-response curve generated with the equation $Y=B+(T-B)/1+10^{((LogEC50-X)\times Hill\ Slope)}$, where Y=percent activity, B=minimum percent activity, T=maximum percent activity, X= logarithm of compound and Hill Slope=slope factor or Hill coefficient. The EC50 or IC50 value was determined by the concentration causing a half-maximal percent activity.

Statistical analysis

Experimental data are presented as mean ± SD. Statistical analyses were performed using GraphPad Prism 9. Comparisons were analyzed by using One-way analysis of variance (ANOVA) followed by Dunnett's post-hoc comparison tests. A p value of <0.05 was considered statistically significant.

Protein production

A variant of human PKL (Uniprot: P30613-2) with S12D phosphomimic mutation and replacement of the mobile B-domain (aa 130–230) by a Gly-Ser-Gly linker was produced in *E. coli* with a T7 promoter-based pExp-NHis expression vector (Addgene plasmid #112558), with an N-terminal , TEV-cleavable His_8-tag. The protein was produced in T7 Express cells in 2xYT media and expression carried out at 18 °C after induction with 0.4 mM IPTG. The cells were harvested by centrifugation and resuspended in 50 ml of 20 mM Tris-HCl pH 8.5, 500 mM NaCl, 20 mM Na phosphate, 20 mM imidazole, 0.5 mM TCEP, 1 mM AEBSF. Clarified lysate, after

lysis using Emulsiflex C5 and centrifugation at 20,000 *g* for 30 minutes, was loaded onto a Ni-NTA affinity column equilibrated with the lysis buffer. After washing, PKL was eluted with the same buffer containing 250 mM imidazole. Eluted protein was buffer exchange to 20 mM Tris-HCl pH 8.5, 25 mM NaCl, 20 mM imidazole, 0.5 mM TCEP and the N-terminal His-tag was removed with in-house produced TEV protease (1:100 ratio TEV:PKL) overnight at 4 °C. Uncleaved protein and TEV protease were captured with a reverse Ni-NTA column and the tag-free PKL was loaded onto 5ml HiTrap Q HP anion exchange column (Cytiva) equilibrated with 20 mM Tris-HCl pH 8.5 and eluted with a liner gradient of NaCl. Peak fractions were pooled, concentrated using 15 kDa MWCO centrifugal concentrators and polished by size exclusion chromatography using a Superdex 200 16/600 column (Cytiva) equilibrated with 20 mM Tris-HCl pH 8.5, 200 mM NaCl, 50 mM KCl, 0.5 mM TCEP, 10% glycerol. Purified PKL, exchanged to 20 mM HEPES-NaOH pH 7.2, 200 mM NaCl, 50 mM KCl, 5% glycerol, 0.5 mM TCEP using a PD-10 column, was concentrated to 7.5 mg/mL, supplemented with 1 mM fructose 1,6-bisphosphate (FBP), aliquoted and flash-frozen in liquid nitrogen.

X-ray crystallography

Purified, FBP-bound PKL was crystalized in 500 nl drops by mixing equal volumes of protein at 7.5 mg/ml with mother liquor containing 100 mM HEPES-MOPS pH7.5, 10% PEG 8000, 20% EG, 10 mM phenylalanine, 20 mM sodium oxalate. Small plate-like crystals appeared typically after 12h. Compounds were soaked to crystals for 12-24 hours by adding the compound directly to the crystallisation drop to final concentrations of 1 mM or more. After overnight soak, the crystals were cryo-cooled in liquid nitrogen before shipping to Diamond Light Source for data collection. Typically, 3-4 parallel soaks were performed to ensure at least one resulted in a high-quality dataset.

Diffraction data was collected using fully automated process at Diamond Light Source, typically 1800 images with 0.1° oscillation per image. The data was processed automatically with autoPROC and Staraniso packages (Global Phasing), resulting typically in datasets around 2.0 Å in resolution with good merging statistics. The structures were solved by molecular replacement as part of pipedream package (Global Phasing) and after manual correction in Coot, refined with Buster. Ligand constraints were generated using Grade2 (Global Phasing) and Mogul (CCDC).

All structures were deposited to Protein Data Bank with accession numbers 7G2B, 7G2A, 7G29, 7G25, 7G24, 7G26, 7G27, 7G28,7G29, 7G2C, 7G2D and 7G22. Data collection and refinement statistics are shown in Table S1.

NMR spectra

4d

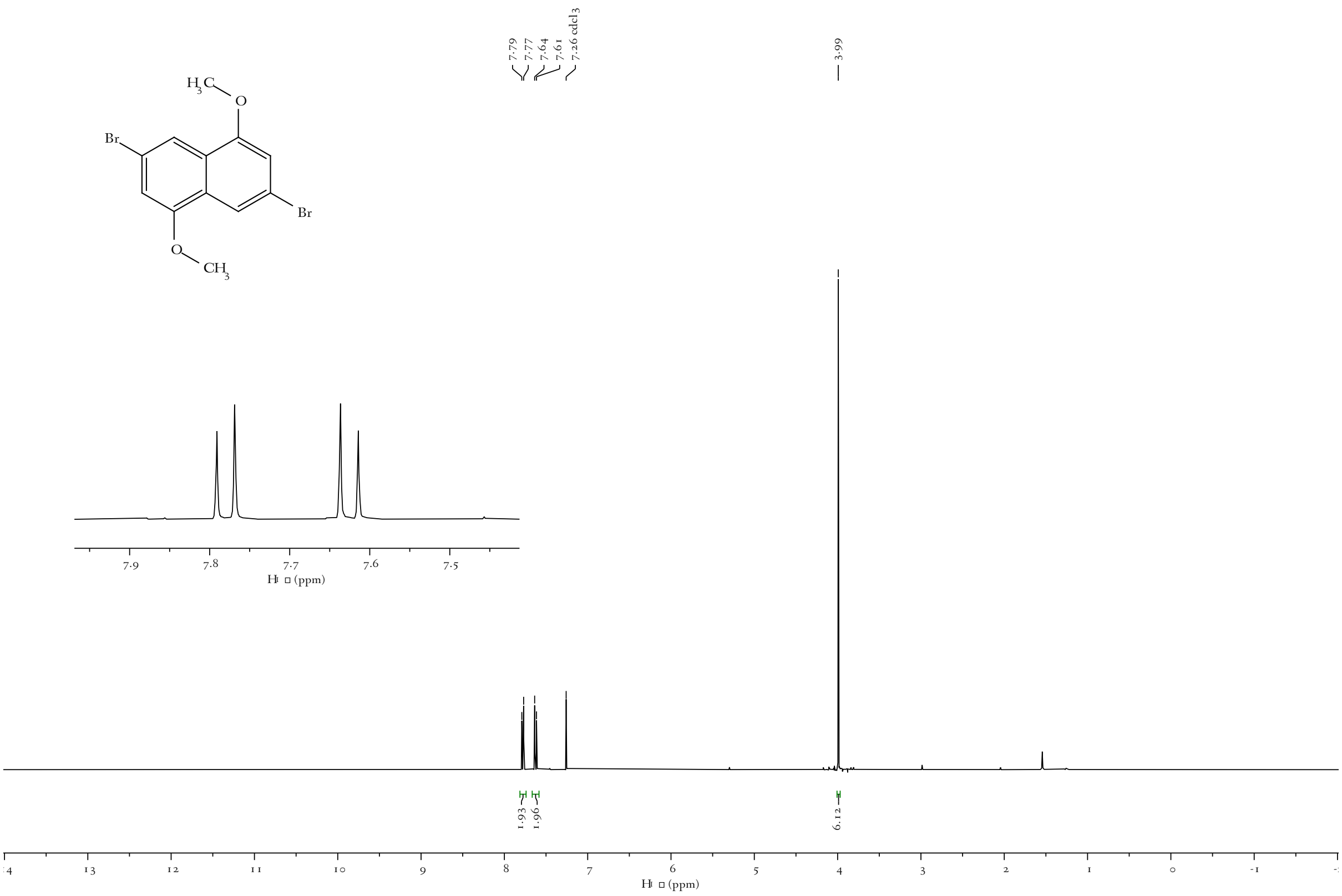

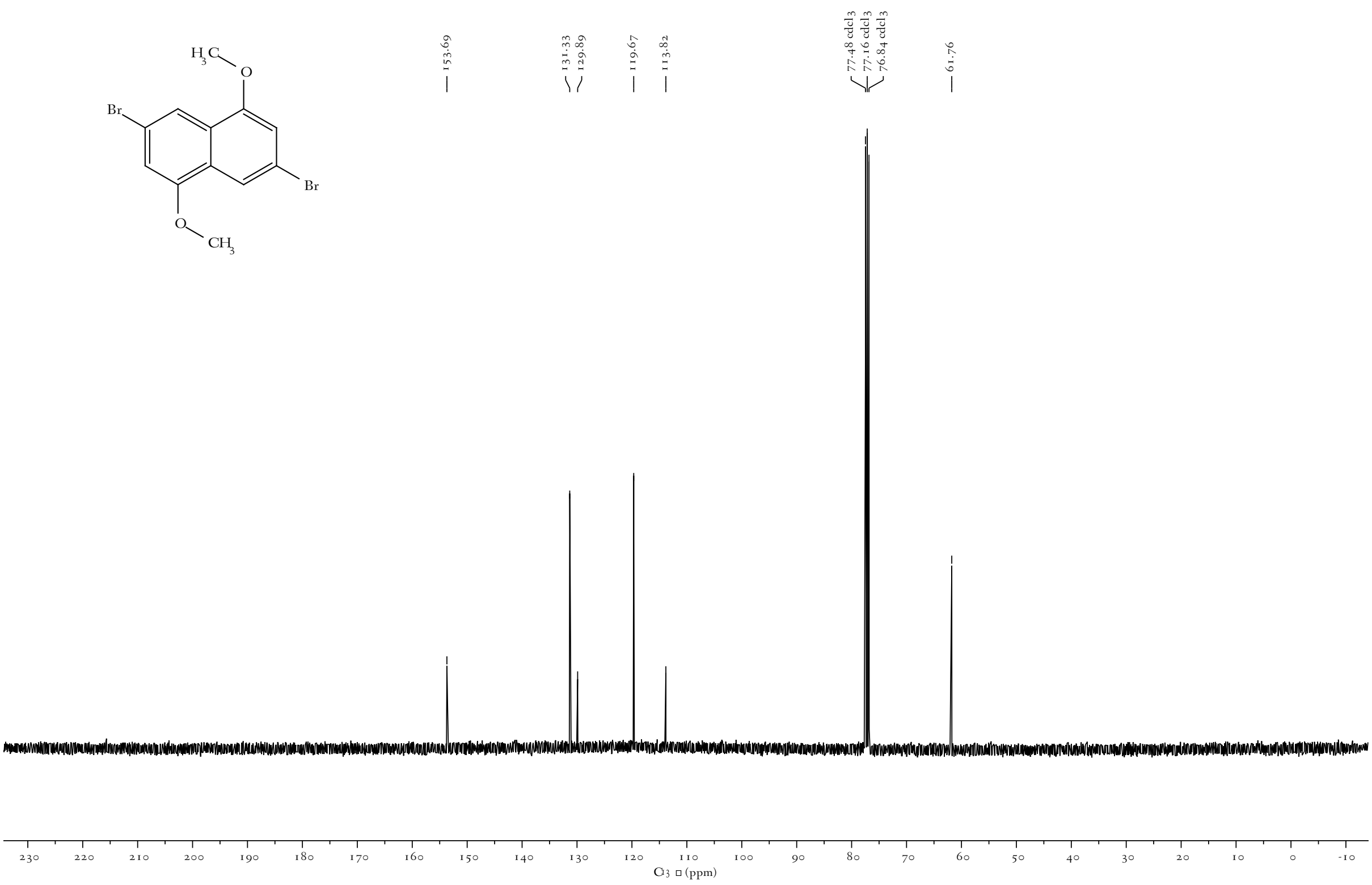

5a

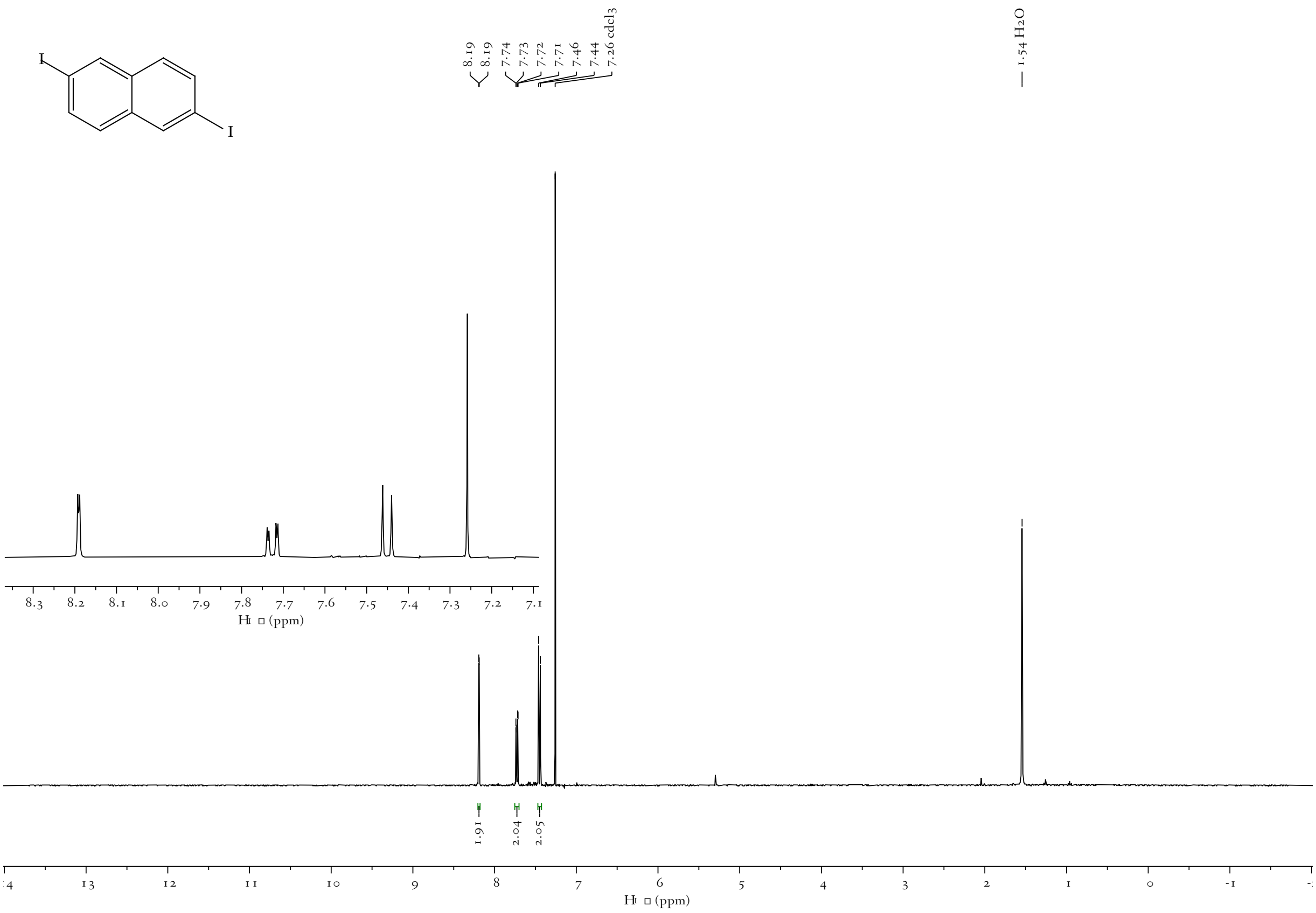
8.19
8.19
7.74
7.73
7.72
7.71
7.46
7.44
7.26 cdcl3
1.54 H2O
1.91
2.04
2.05
H1 (ppm)

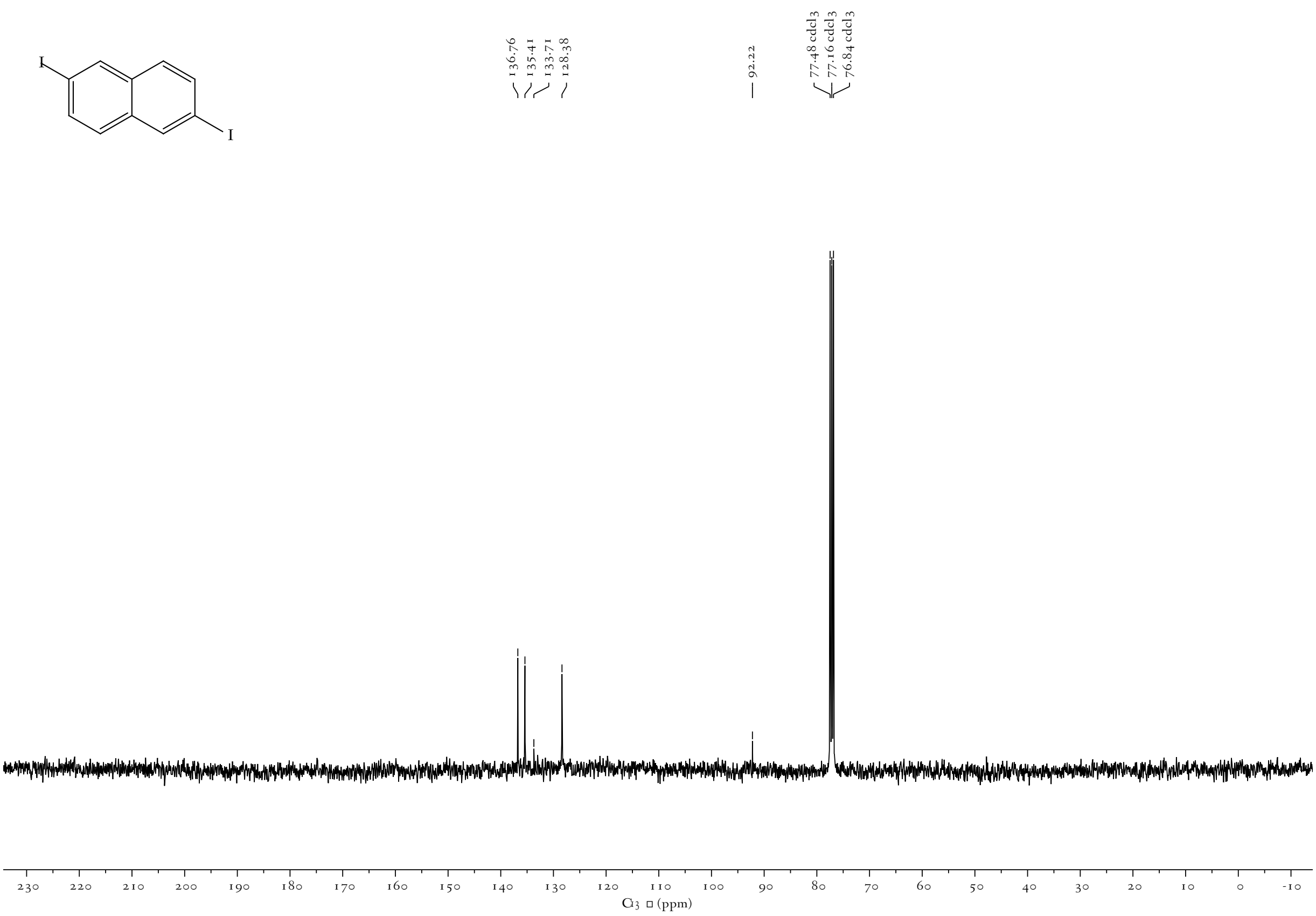
136.76
135.41
133.71
128.38
92.22
77.48 cdcl3
77.16 cdcl3
76.84 cdcl3
C13 (ppm)

5b

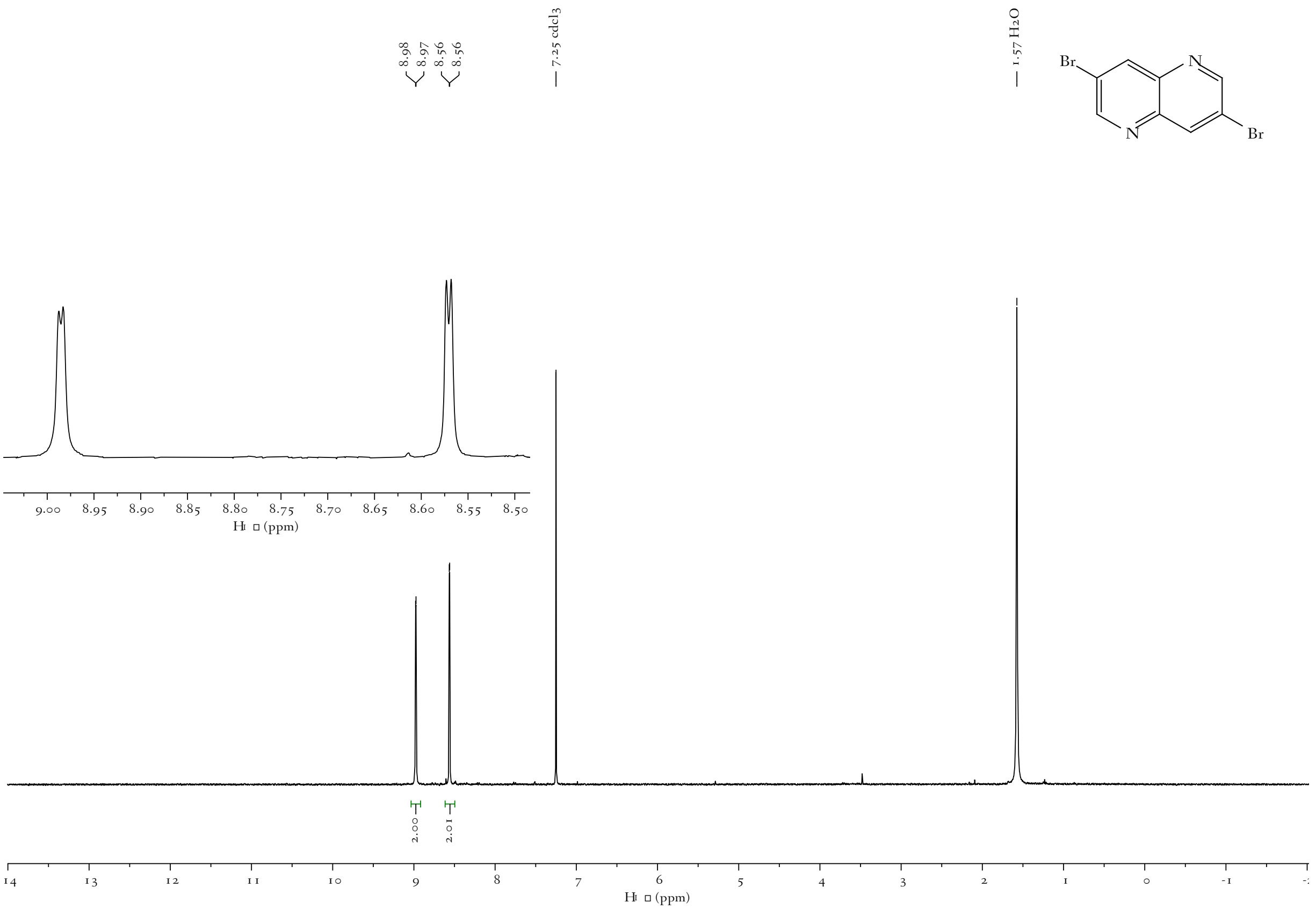
8.98
8.97
8.56
8.56
7.25 cdcl3
1.57 H2O
Br
N
N
Br
9.00 8.95 8.90 8.85 8.80 8.75 8.70 8.65 8.60 8.55 8.50
2.00
2.01
14 13 12 11 10 9 8 7 6 5 4 3 2 1 0 -1
(ppm)

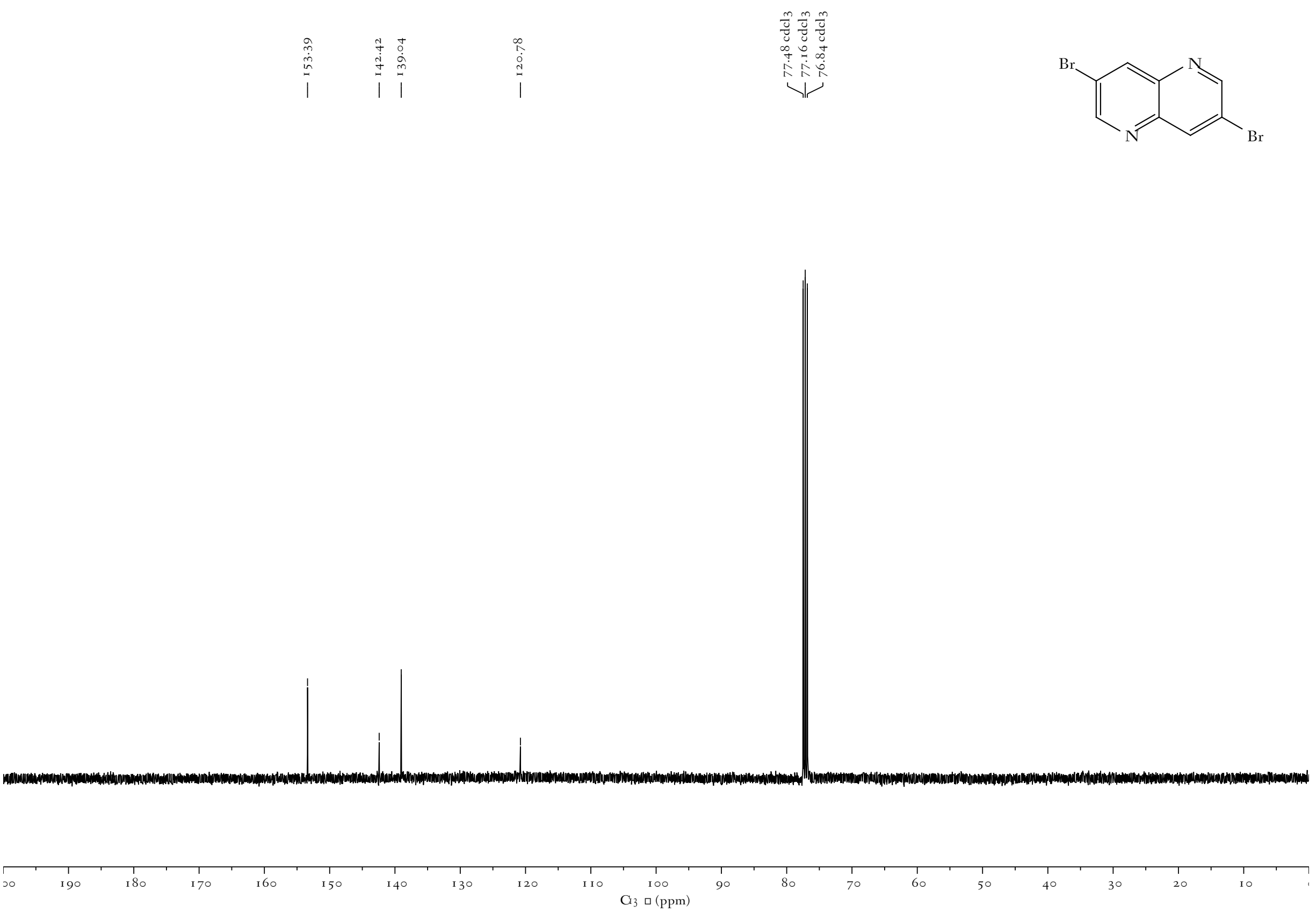
153.39
142.42
139.04
120.78
77.48 cdcl3
77.16 cdcl3
76.84 cdcl3
Br
N
N
Br
190 180 170 160 150 140 130 120 110 100 90 80 70 60 50 40 30 20 10
(ppm)

5c

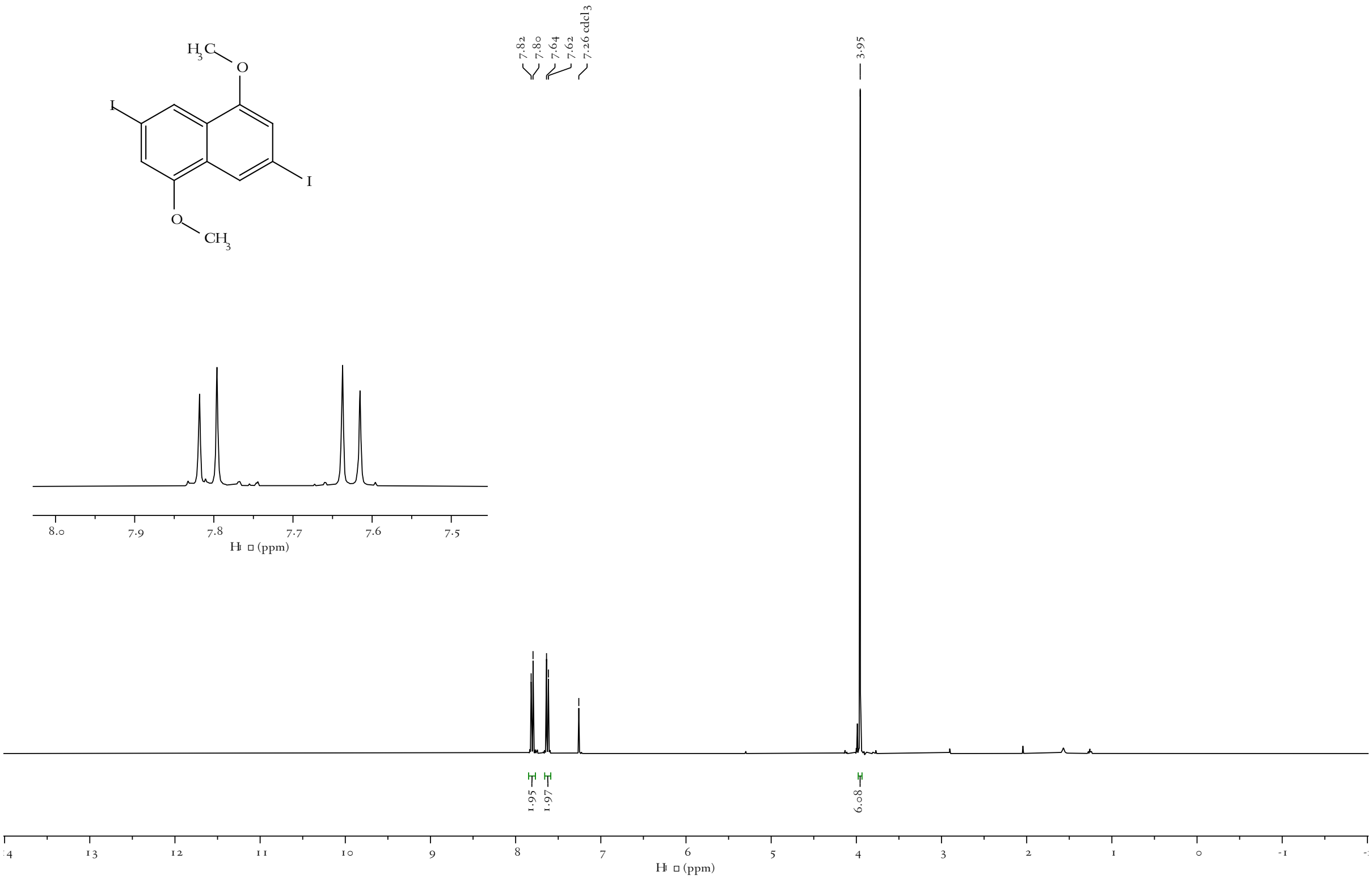
H3C
O
I
I
O
CH3
7.82
7.80
7.64
7.62
7.26 cdcl3
3.95
1.95
1.97
6.08
8.0
7.9
7.8
7.7
7.6
7.5
13
12
11
10
9
8
7
6
5
4
3
2
1
0
-1
(ppm)

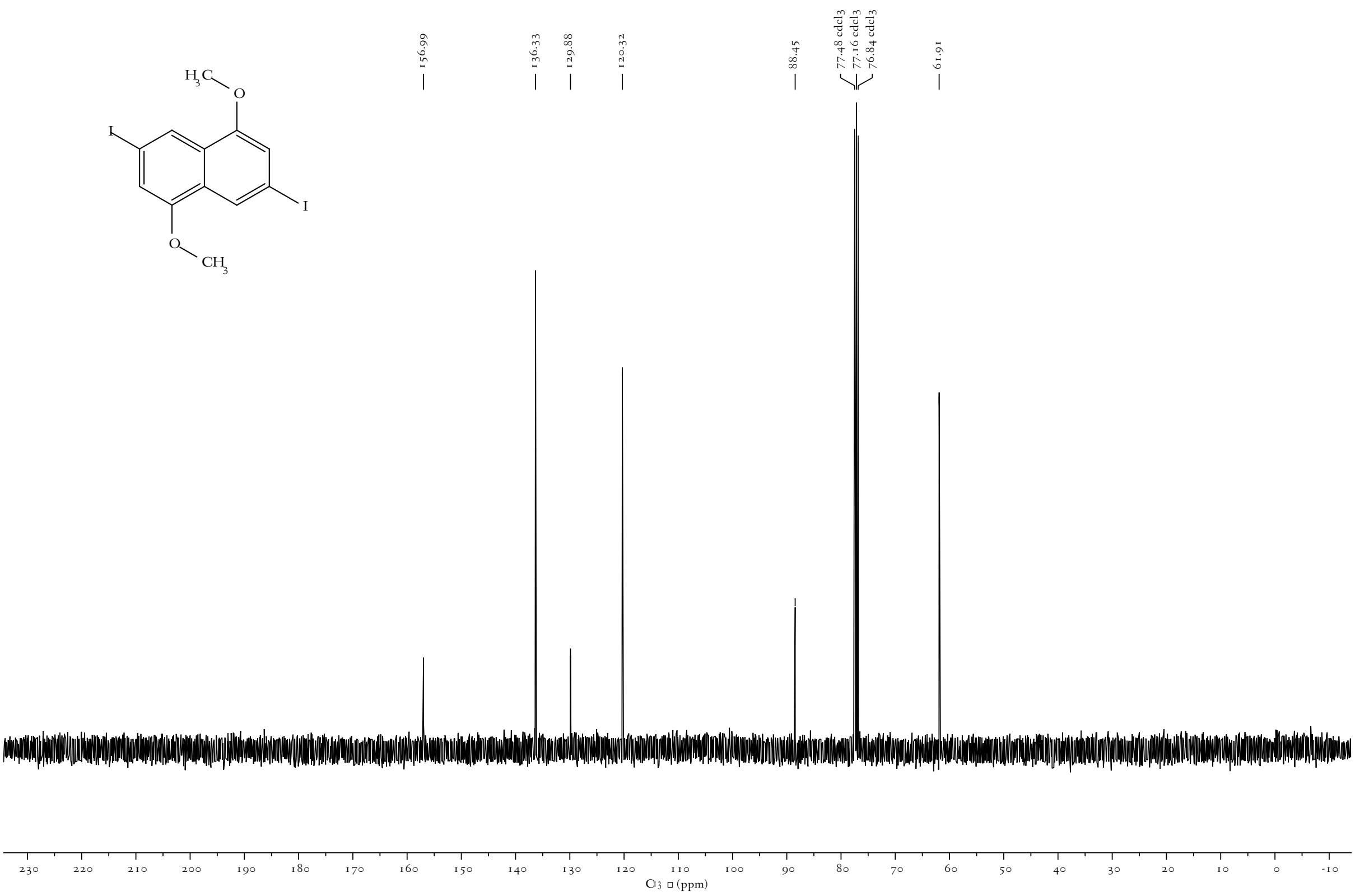
H3C
O
I
I
O
CH3
156.99
136.33
129.88
120.32
88.45
77.48 cdcl3
77.16 cdcl3
76.84 cdcl3
61.91
230
220
210
200
190
180
170
160
150
140
130
120
110
100
90
80
70
60
50
40
30
20
10
0
-10
(ppm)

6a

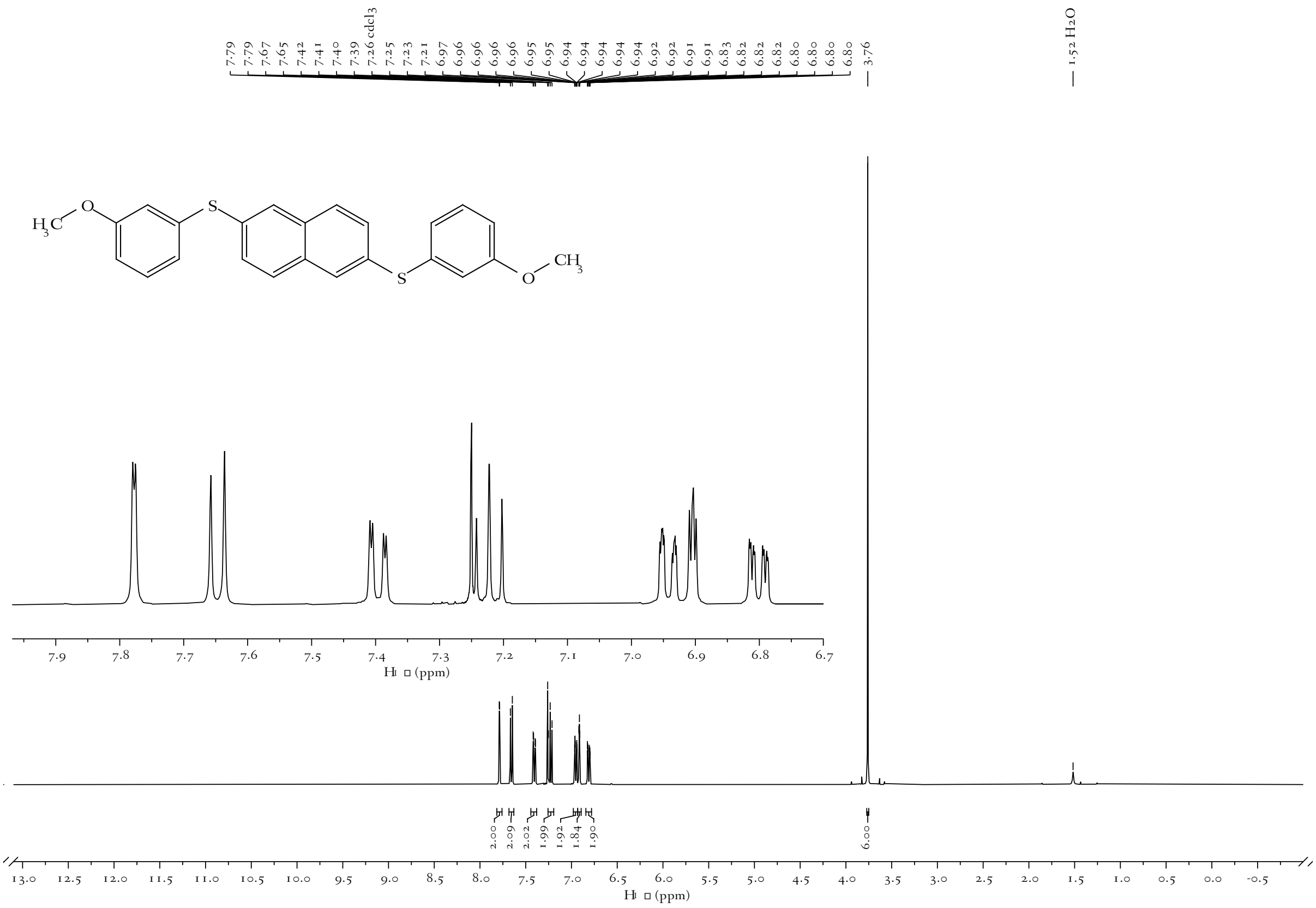

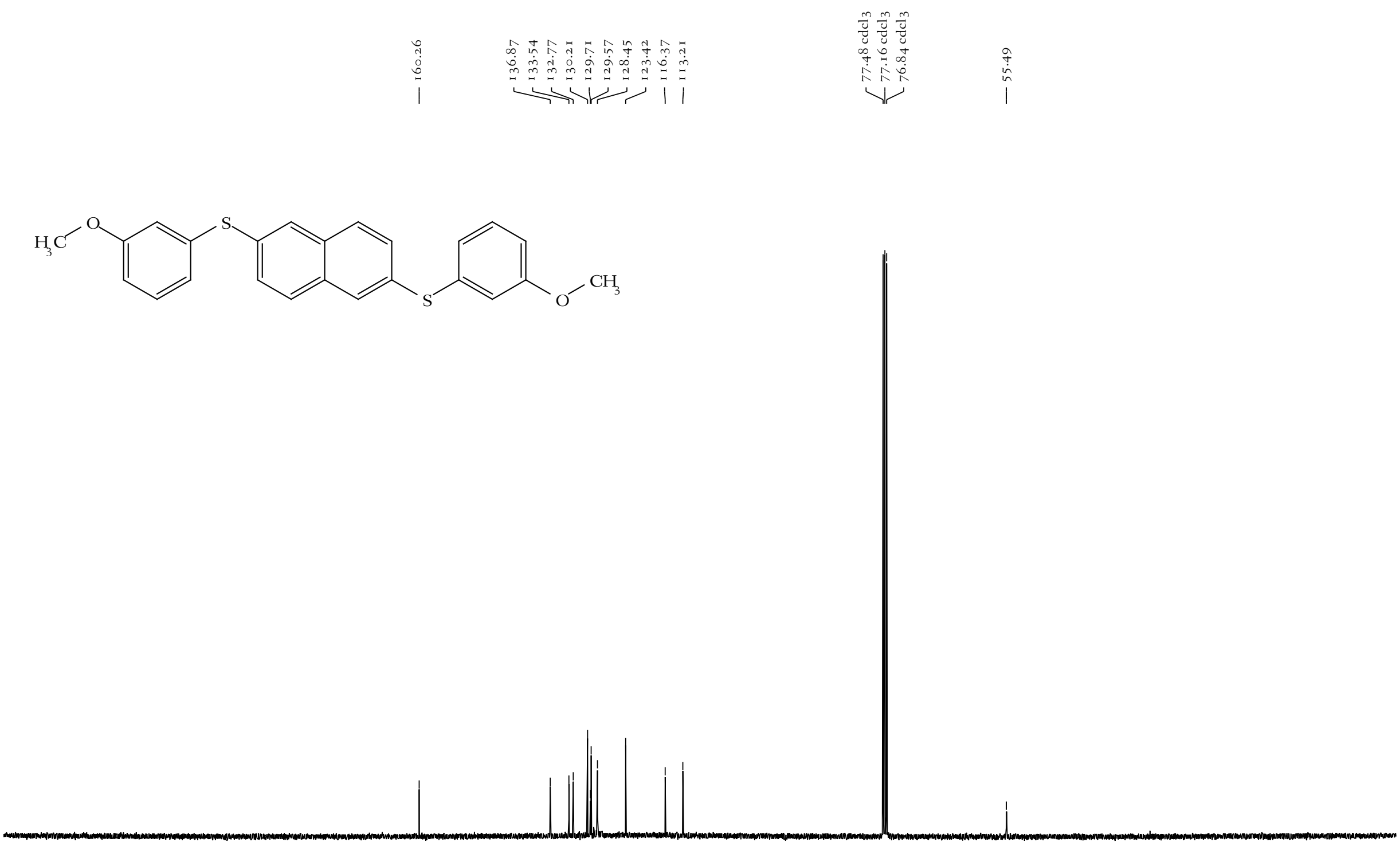

6b

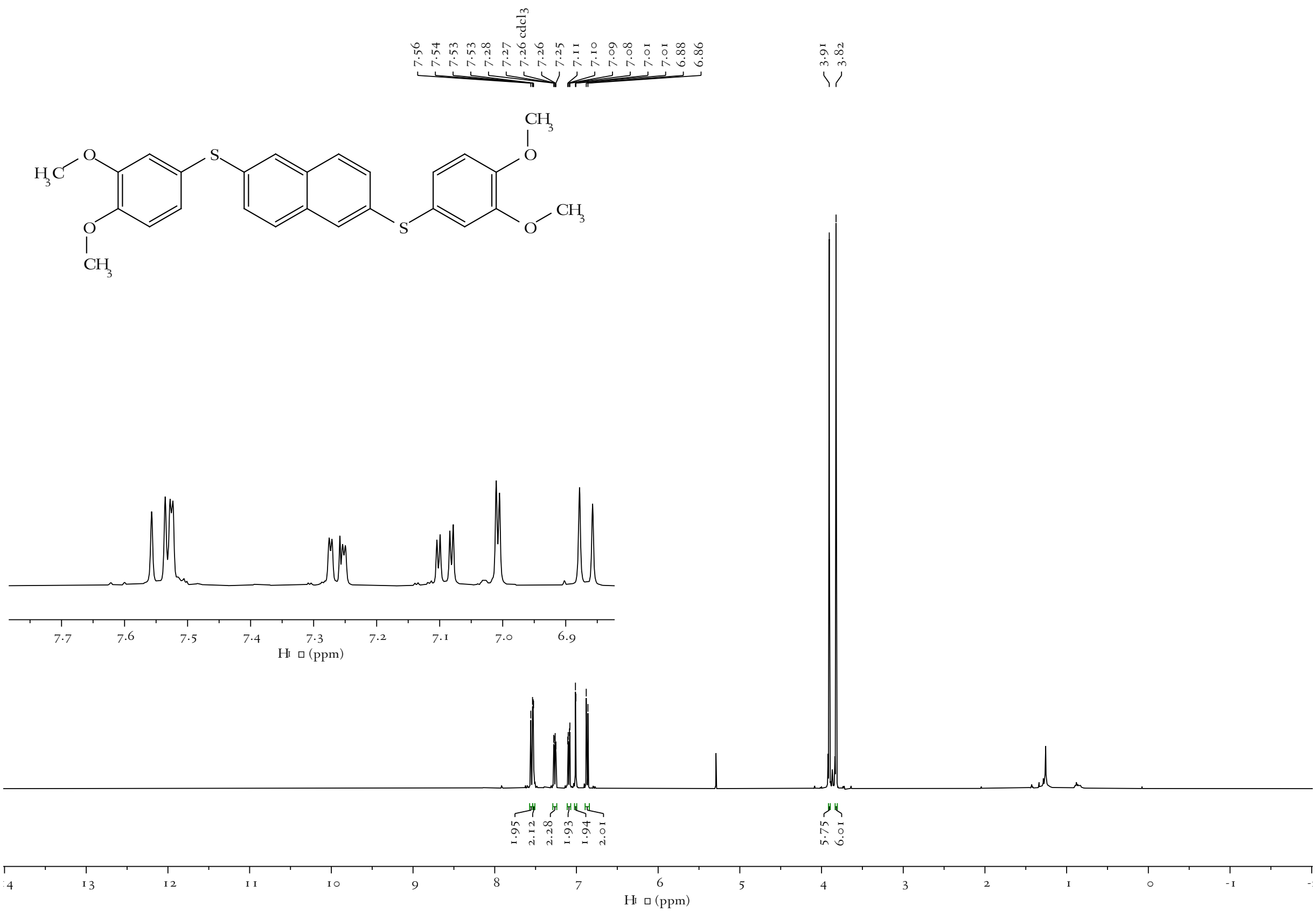

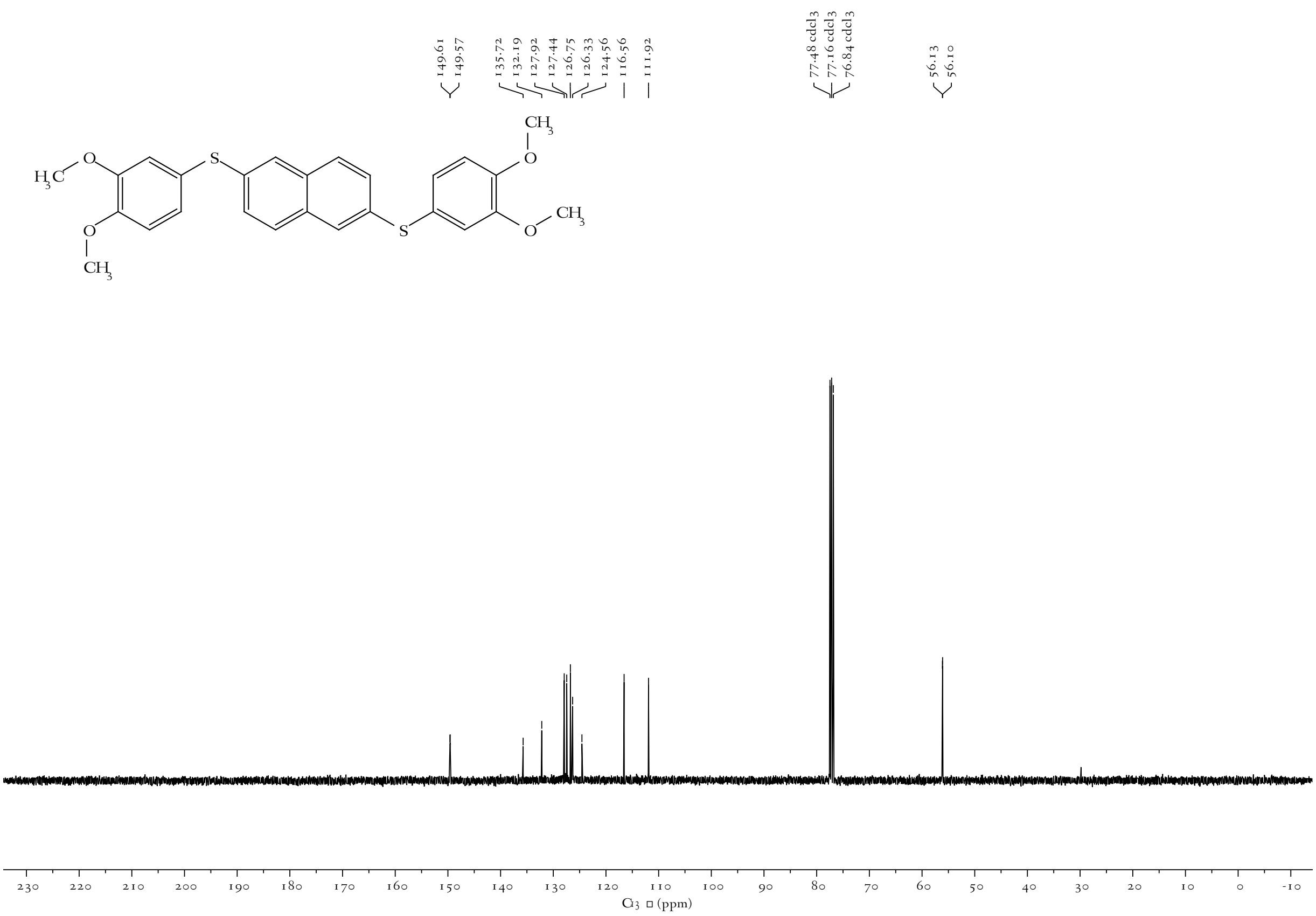

6c

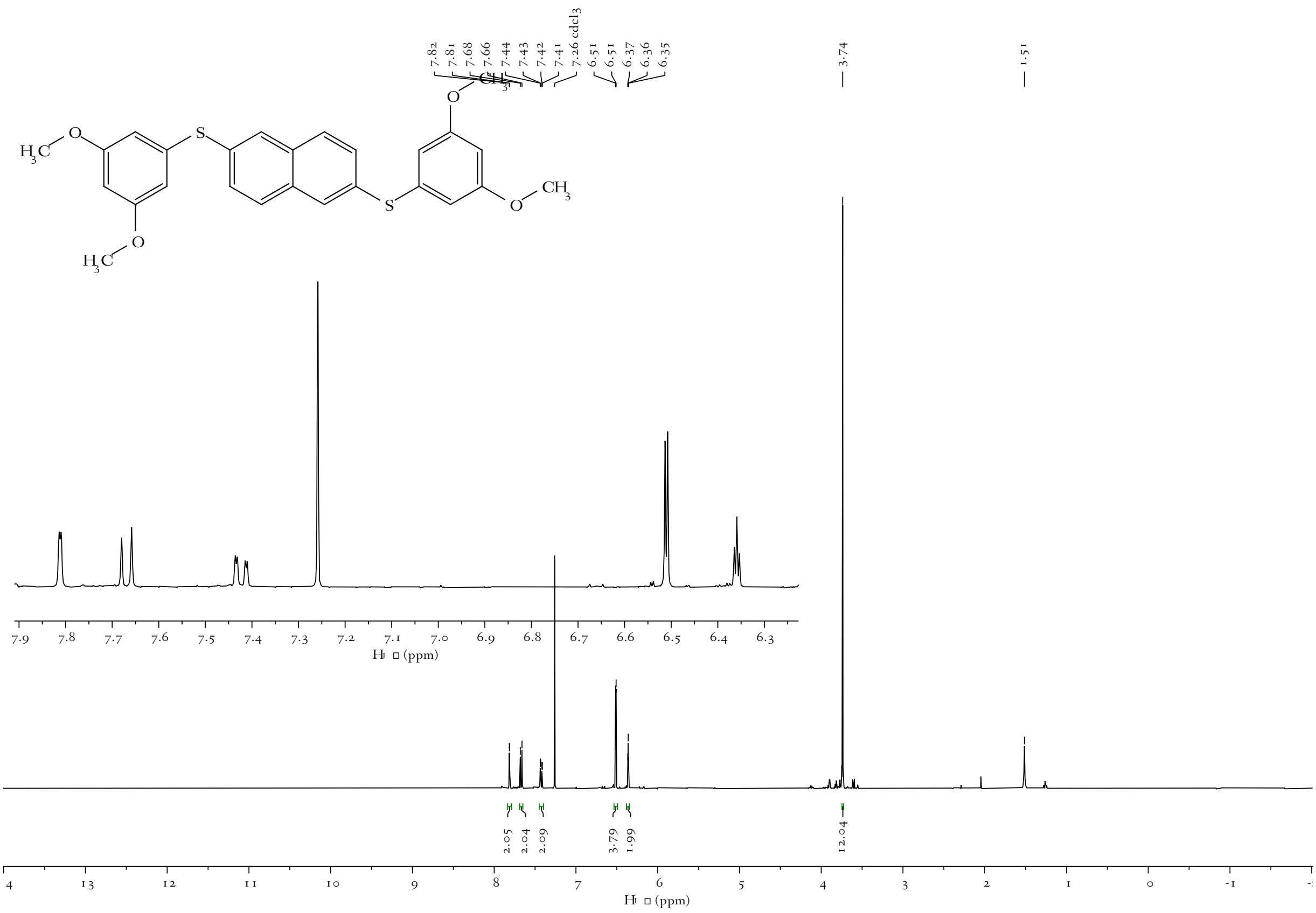
7.82
7.81
7.68
7.66
7.44
7.43
7.42
7.41
7.26 cdcl3
6.51
6.51
6.37
6.36
6.35
3.74
1.51
2.05
2.04
2.09
3.79
1.99
12.04
H₃C
O
S
O
CH₃
(ppm)

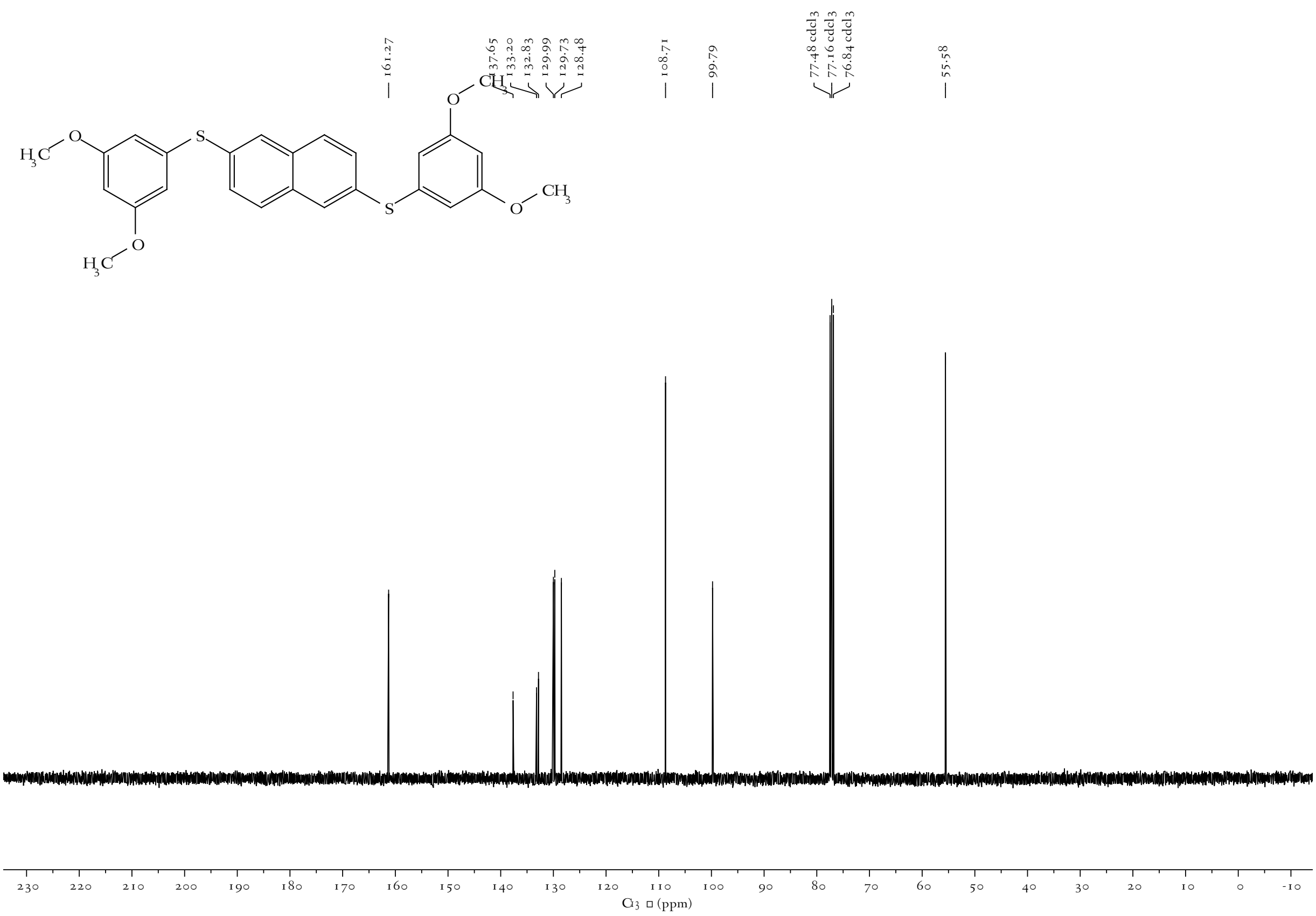
161.27
137.65
133.20
132.83
129.99
129.73
128.48
108.71
99.79
77.48 cdcl3
77.16 cdcl3
76.84 cdcl3
55.58
H₃C
O
S
O
CH₃
(ppm)

6d

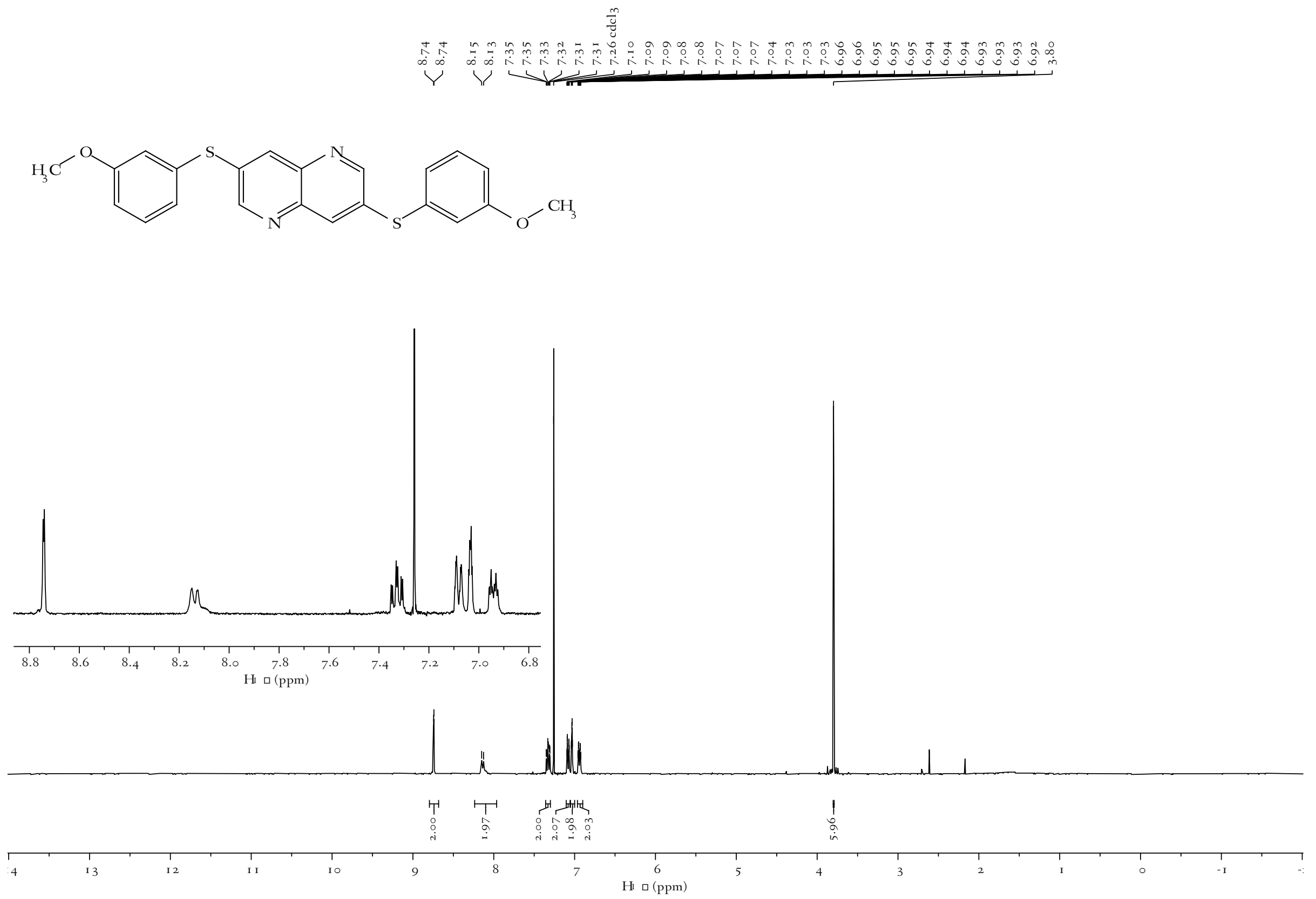

H$_3$C
O
S
N
N
S
O
CH$_3$
8.8
8.6
8.4
8.2
8.0
7.8
7.6
7.4
7.2
7.0
6.8
2.00
1.97
2.00
2.07
1.98
2.03
5.96

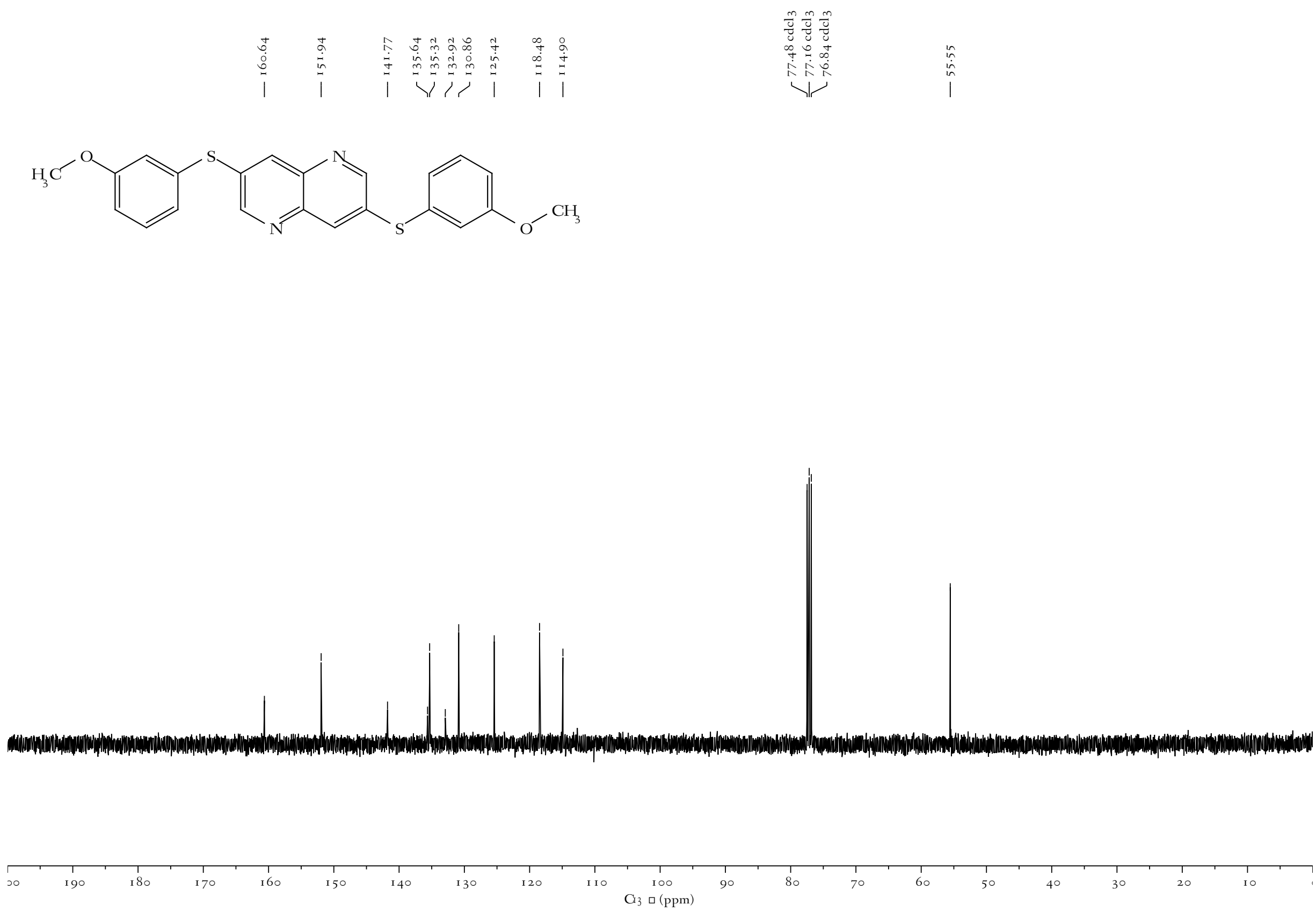

160.64
151.94
141.77
135.64
135.32
132.92
130.86
125.42
118.48
114.90
77.48 cdcl3
77.16 cdcl3
76.84 cdcl3
55.55
H$_3$C
O
S
N
N
S
O
CH$_3$

6e

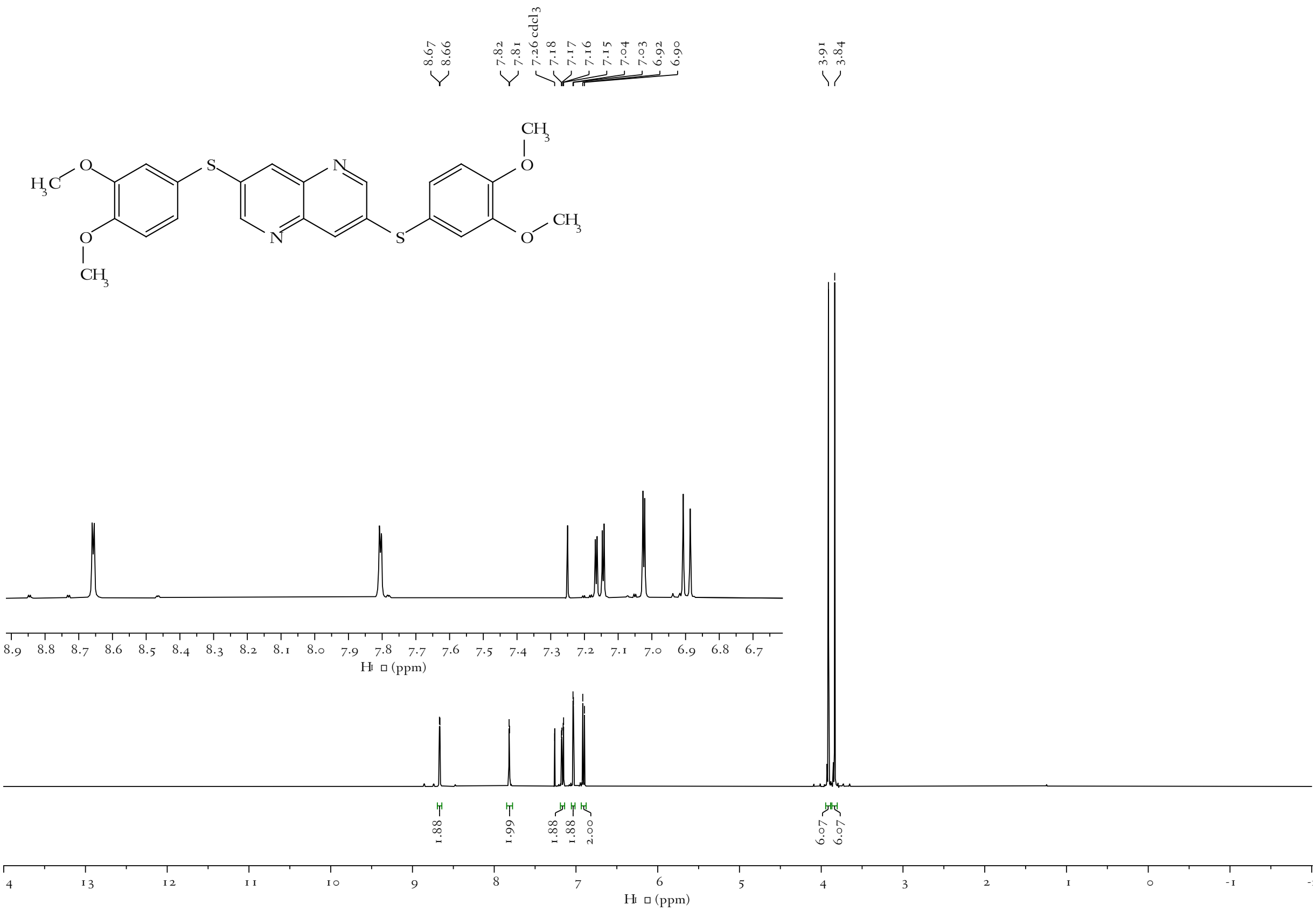
8.67
8.66
7.82
7.81
7.26 cdcl3
7.18
7.17
7.16
7.15
7.04
7.03
6.92
6.90
3.91
3.84
S
N
N
S
O
O
O
O
H_3C
CH_3
CH_3
CH_3
1.88
1.99
1.88
1.88
2.00
6.07
6.07
(ppm)
(ppm)

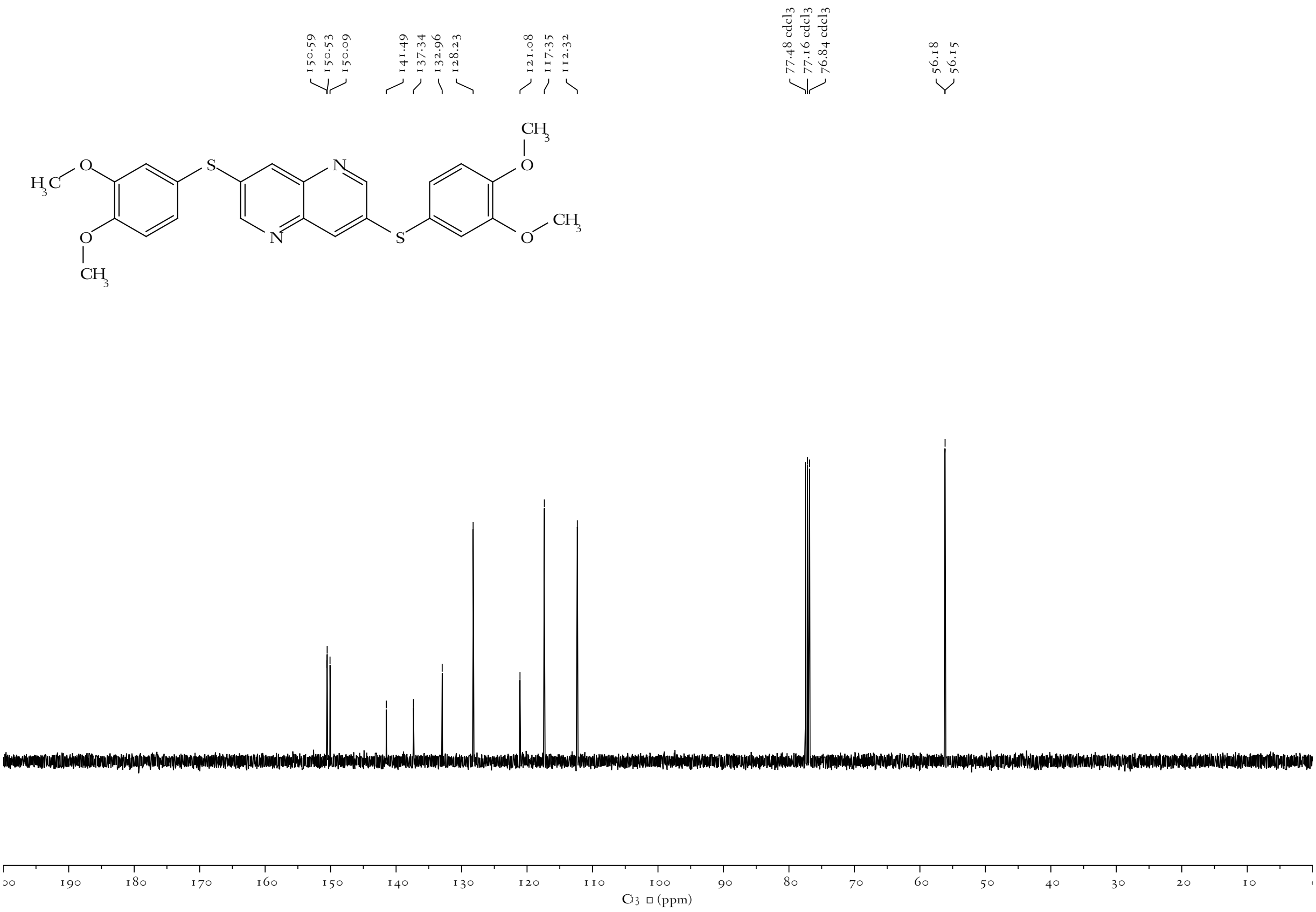
150.59
150.53
150.09
141.49
137.34
132.96
128.23
121.08
117.35
112.32
77.48 cdcl3
77.16 cdcl3
76.84 cdcl3
56.18
56.15
S
N
N
S
O
O
O
O
H_3C
CH_3
CH_3
CH_3
(ppm)

6f

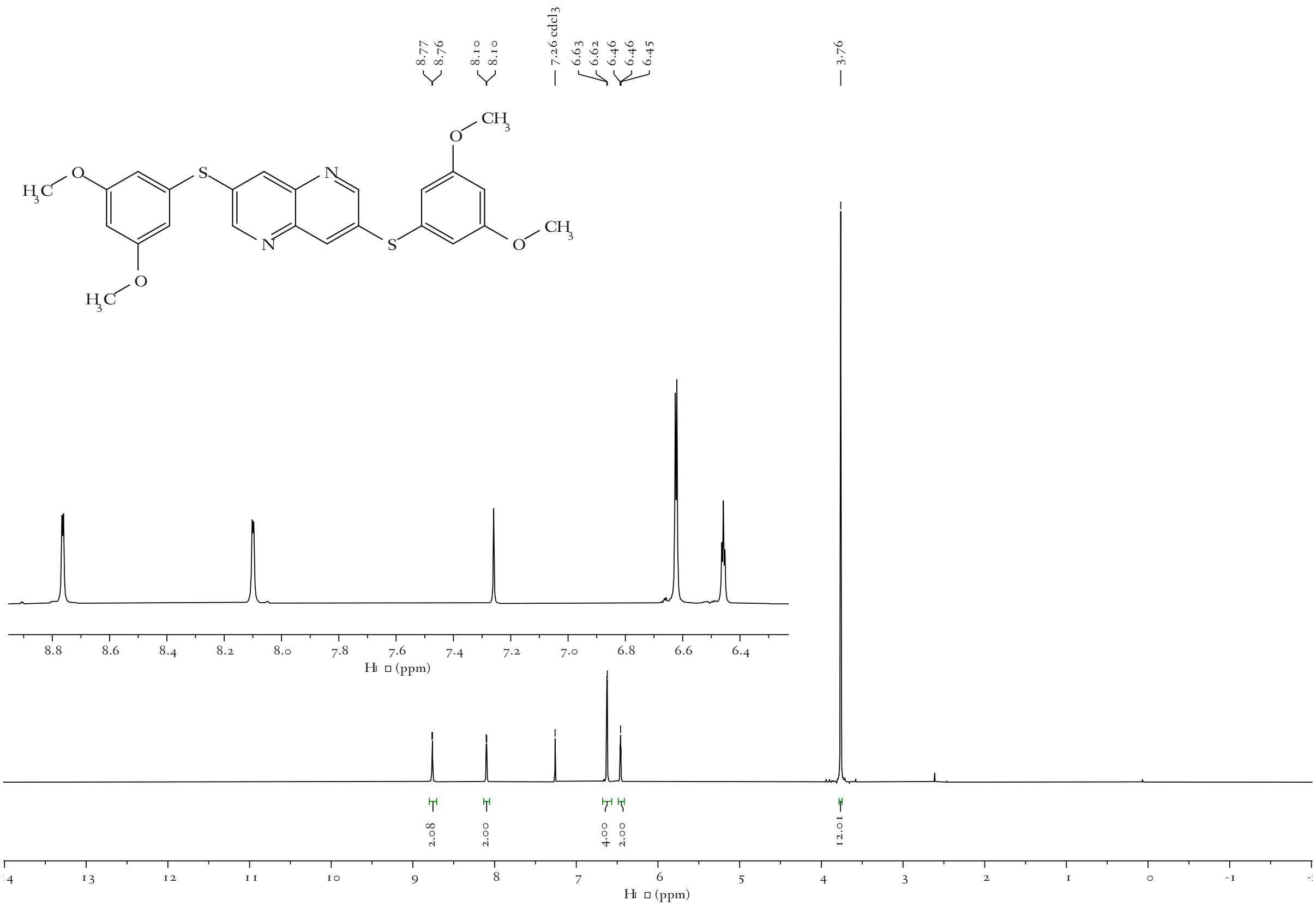

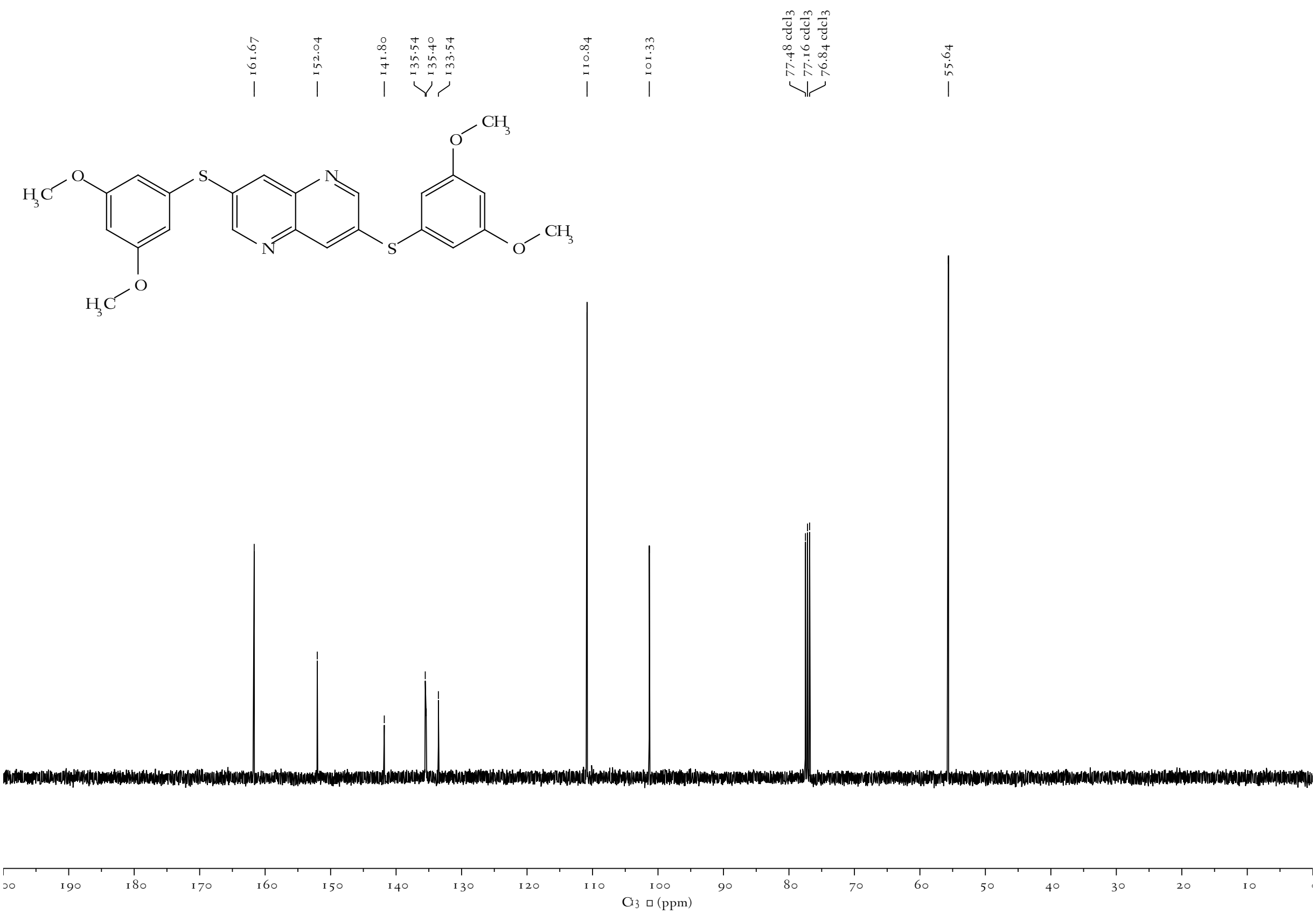

6g

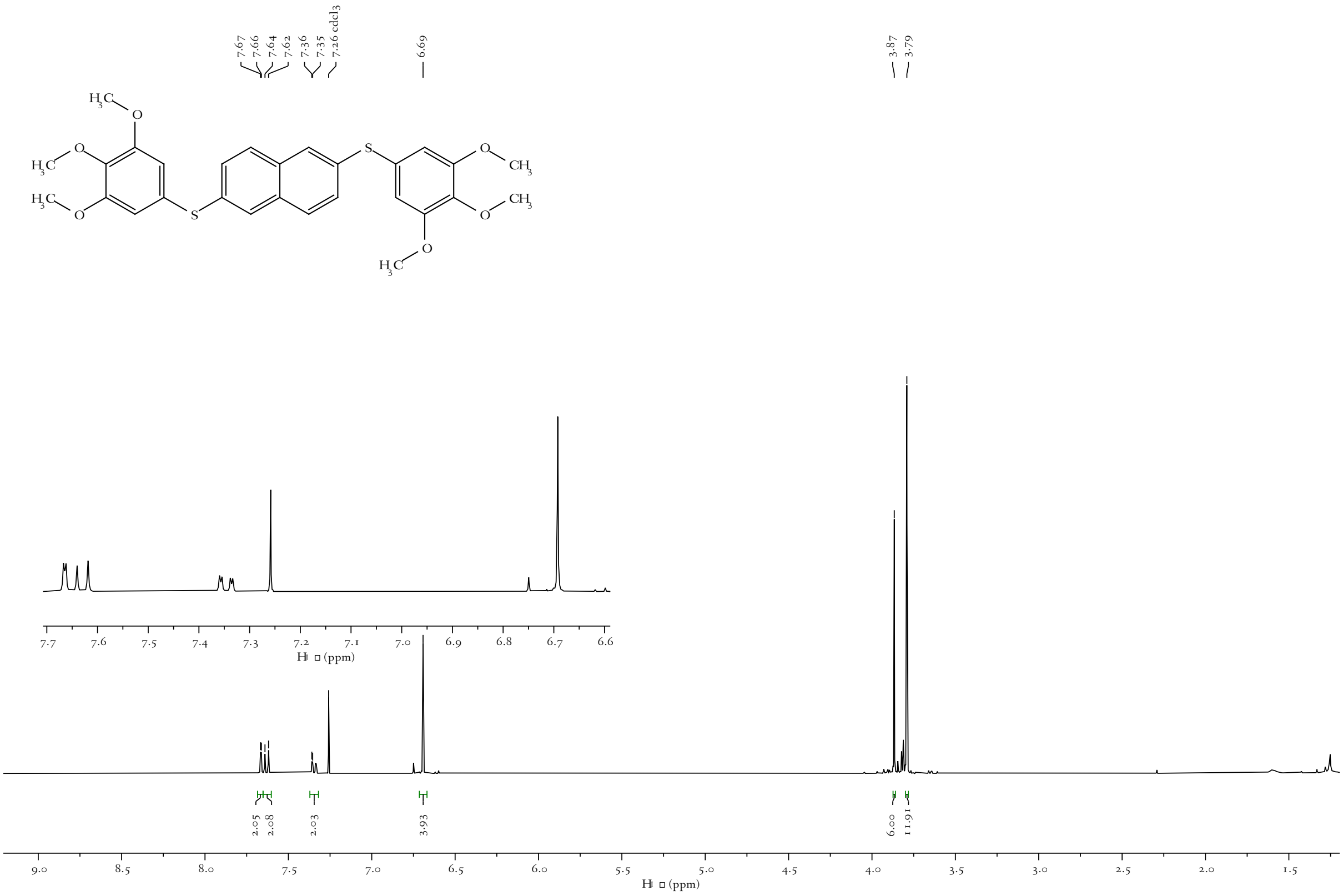
7.67
7.66
7.64
7.62
7.36
7.35
7.26 cdcl3
6.69
3.87
3.79
2.05
2.08
2.03
3.93
6.00
11.91
H1 (ppm)

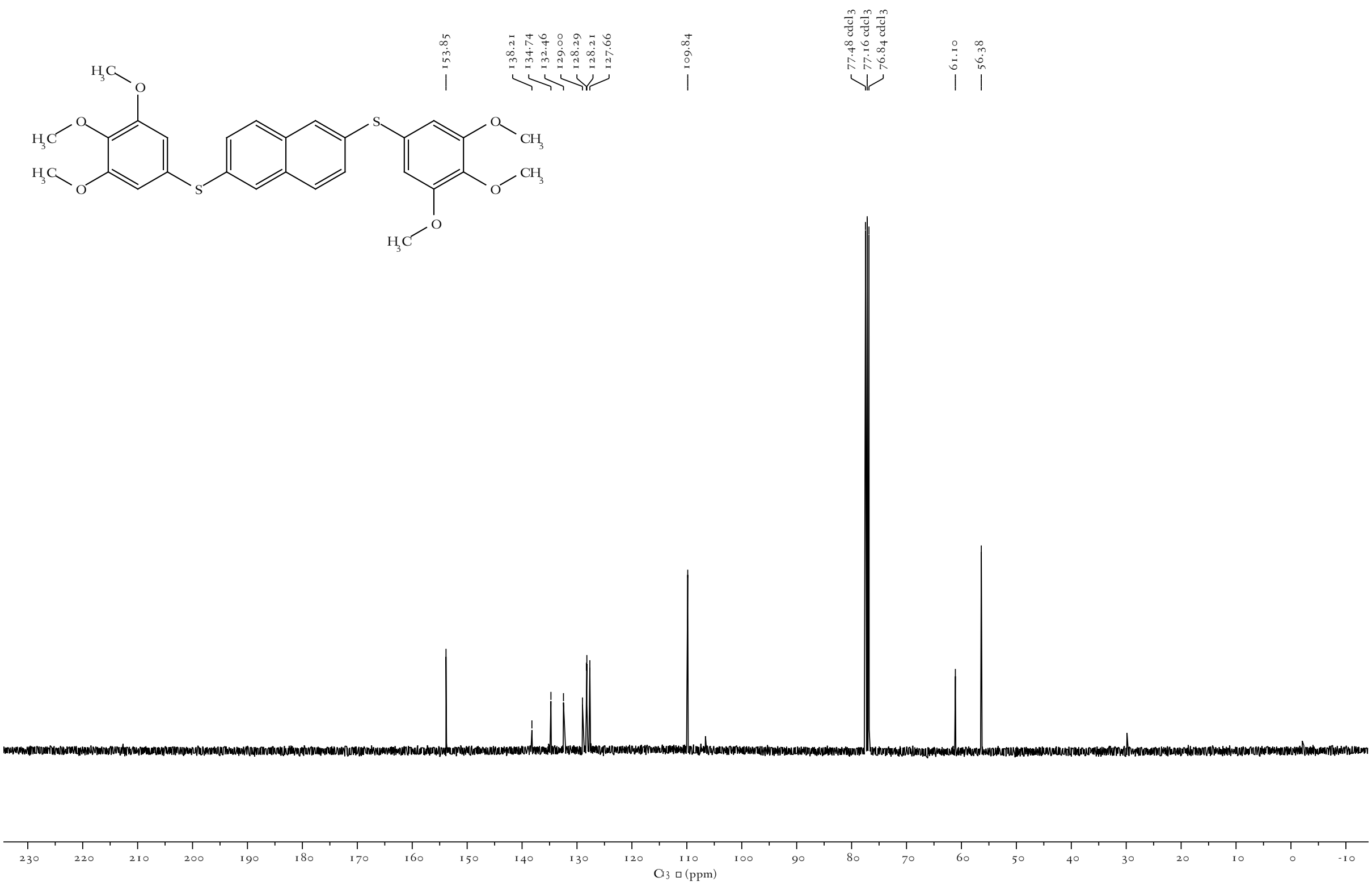
153.85
138.21
134.74
132.46
129.00
128.29
128.21
127.66
109.84
77.48 cdcl3
77.16 cdcl3
76.84 cdcl3
61.10
56.38
C13 (ppm)

6h

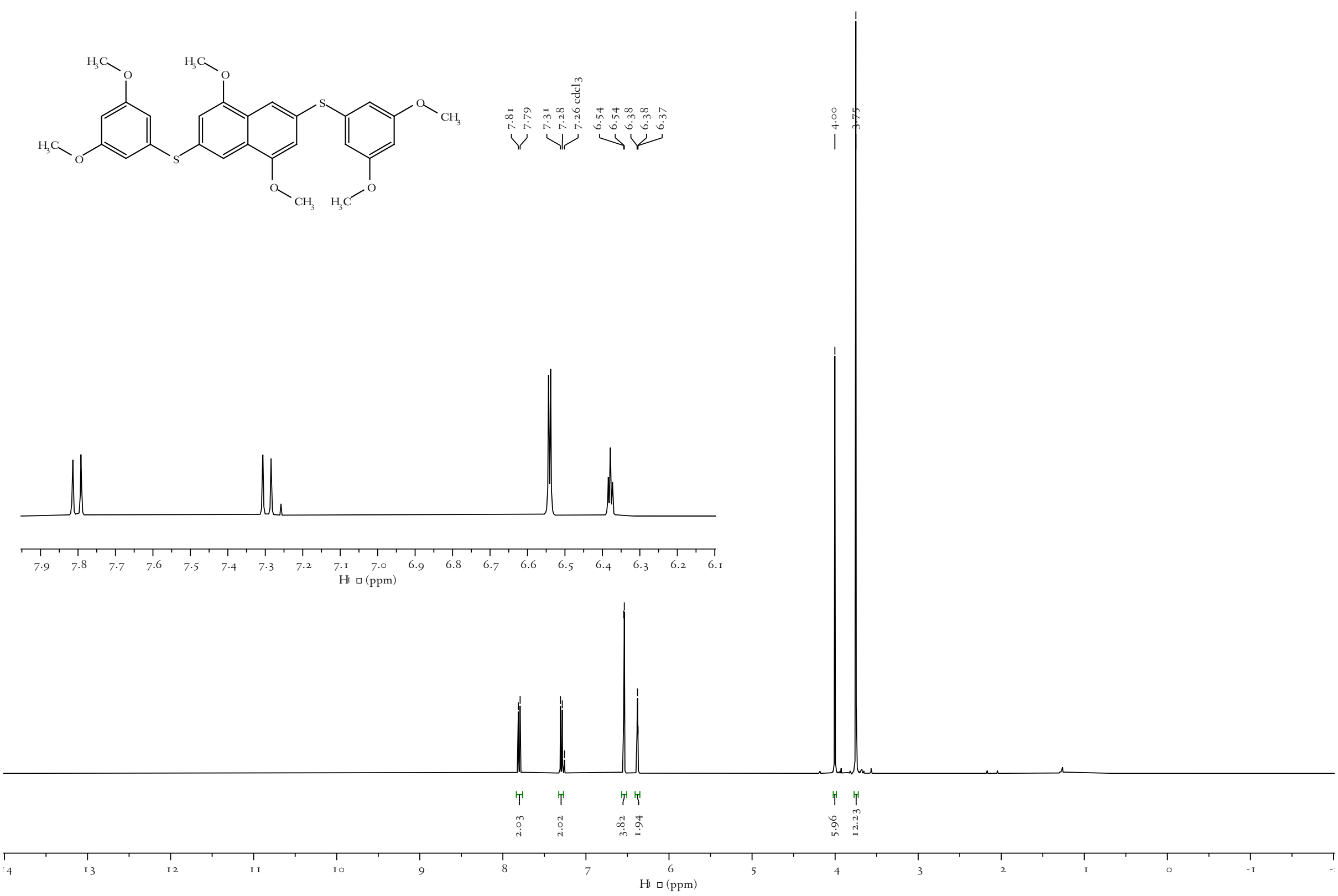
7.81
7.79
7.31
7.28
7.26 cdcl3
6.54
6.54
6.38
6.38
6.37
4.00
3.75
2.03
2.02
3.82
1.94
5.96
12.23

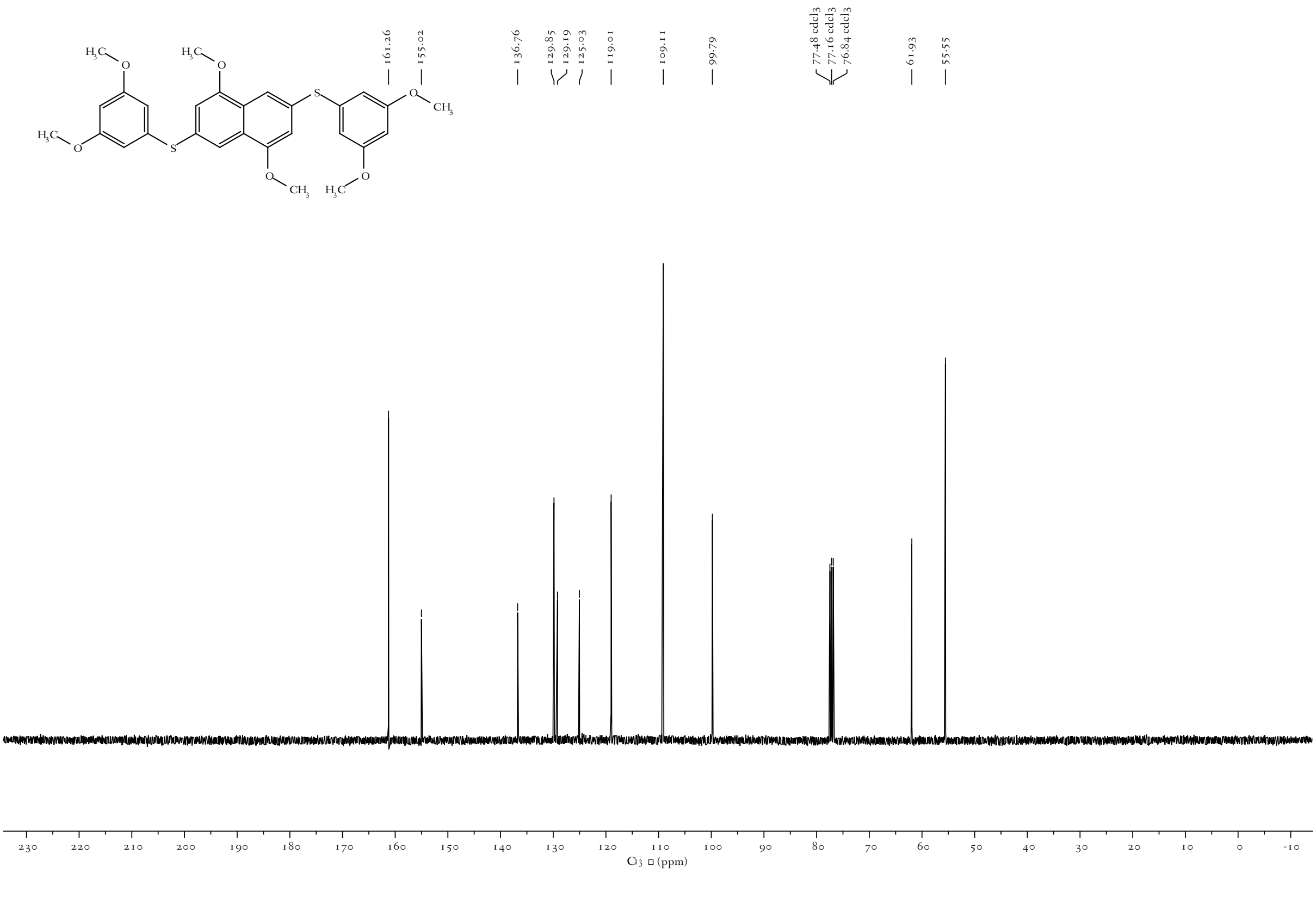
161.26
155.02
136.76
129.85
129.19
125.03
119.01
109.11
99.79
77.48 cdcl3
77.16 cdcl3
76.84 cdcl3
61.93
55.55

7a

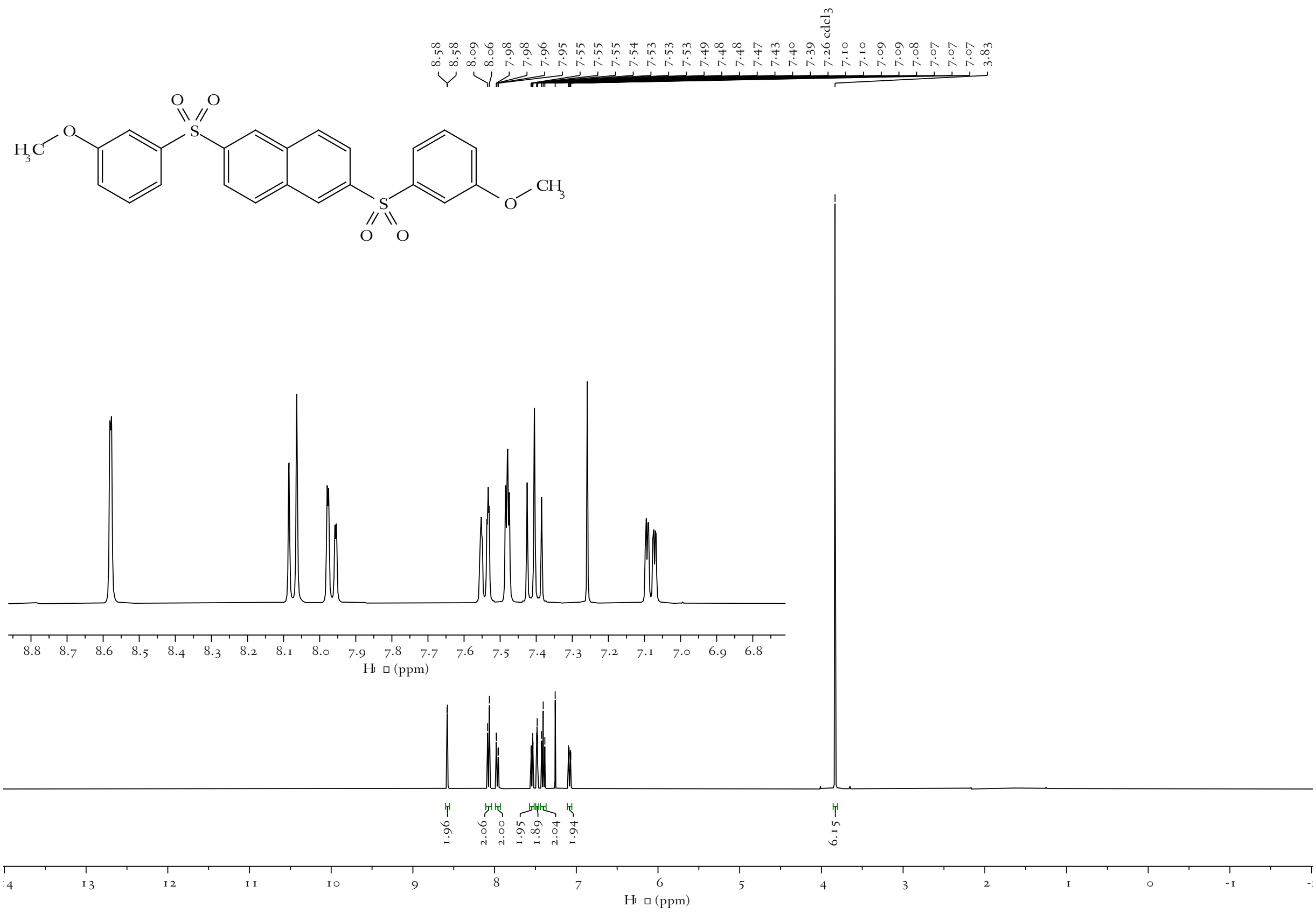

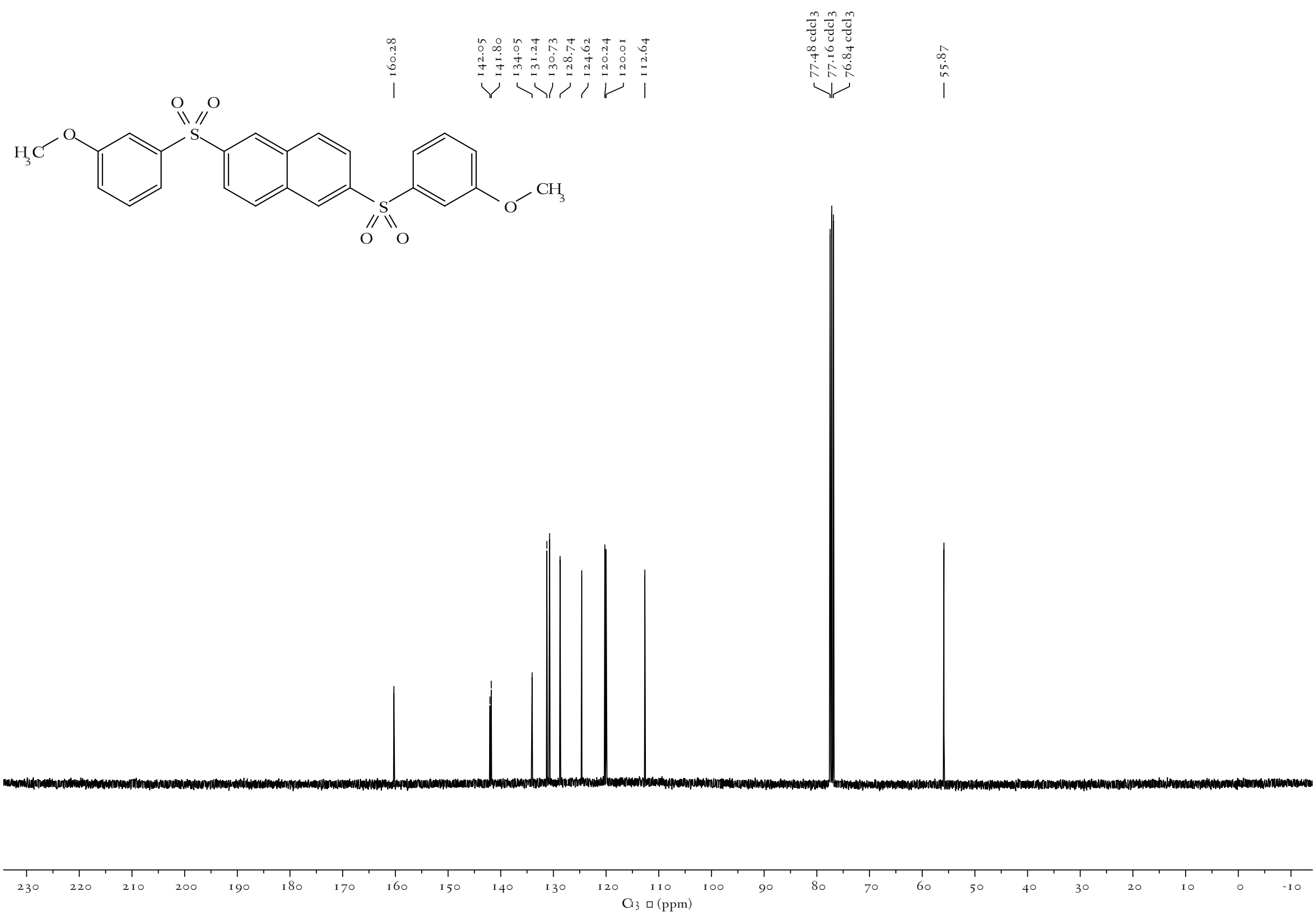

7b

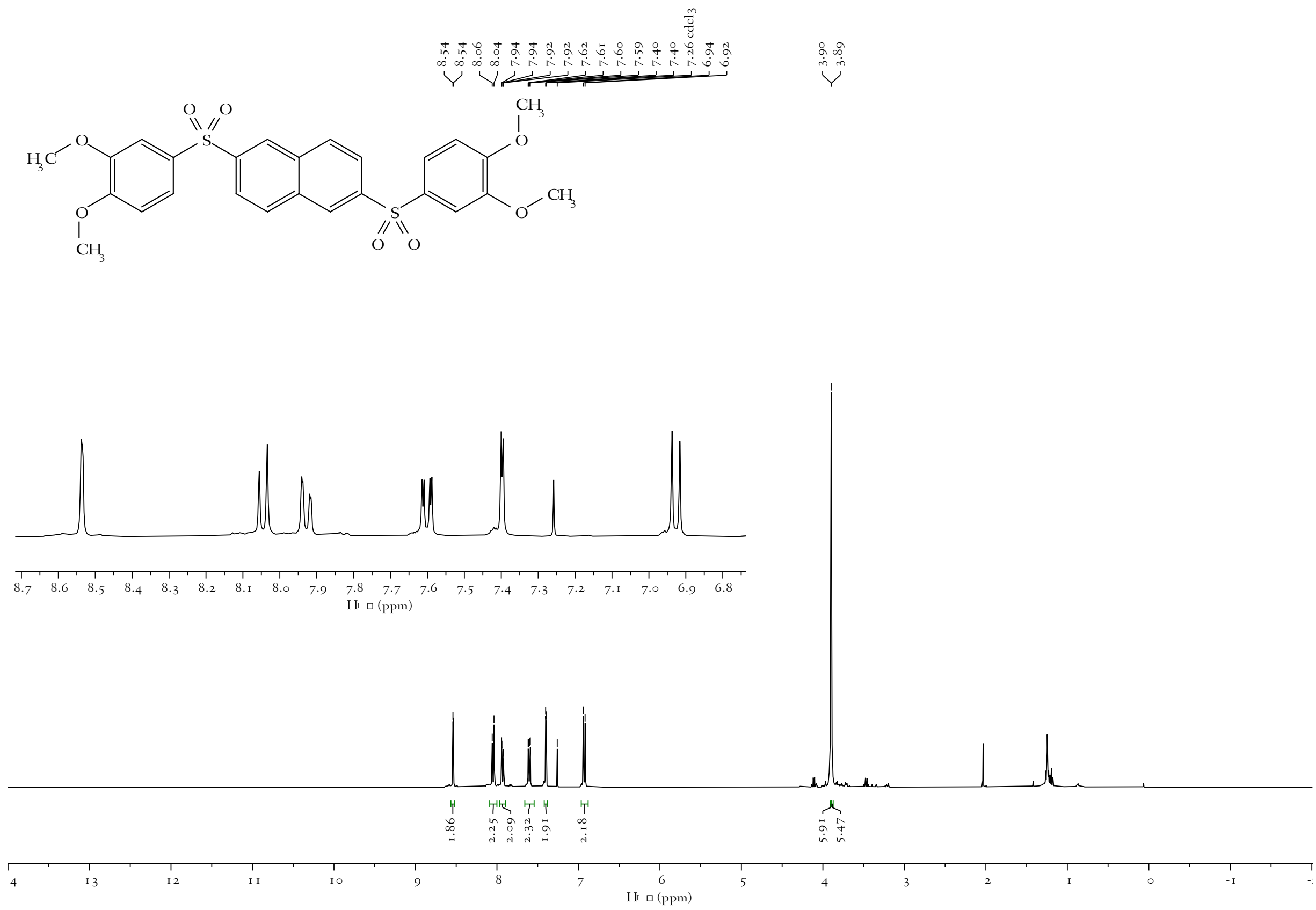

8.54
8.54
8.06
8.04
7.94
7.94
7.92
7.92
7.62
7.61
7.60
7.59
7.40
7.40
7.26 cdcl3
6.94
6.92
3.90
3.89
1.86
2.25
2.09
2.32
1.91
2.18
5.91
5.47
(ppm)

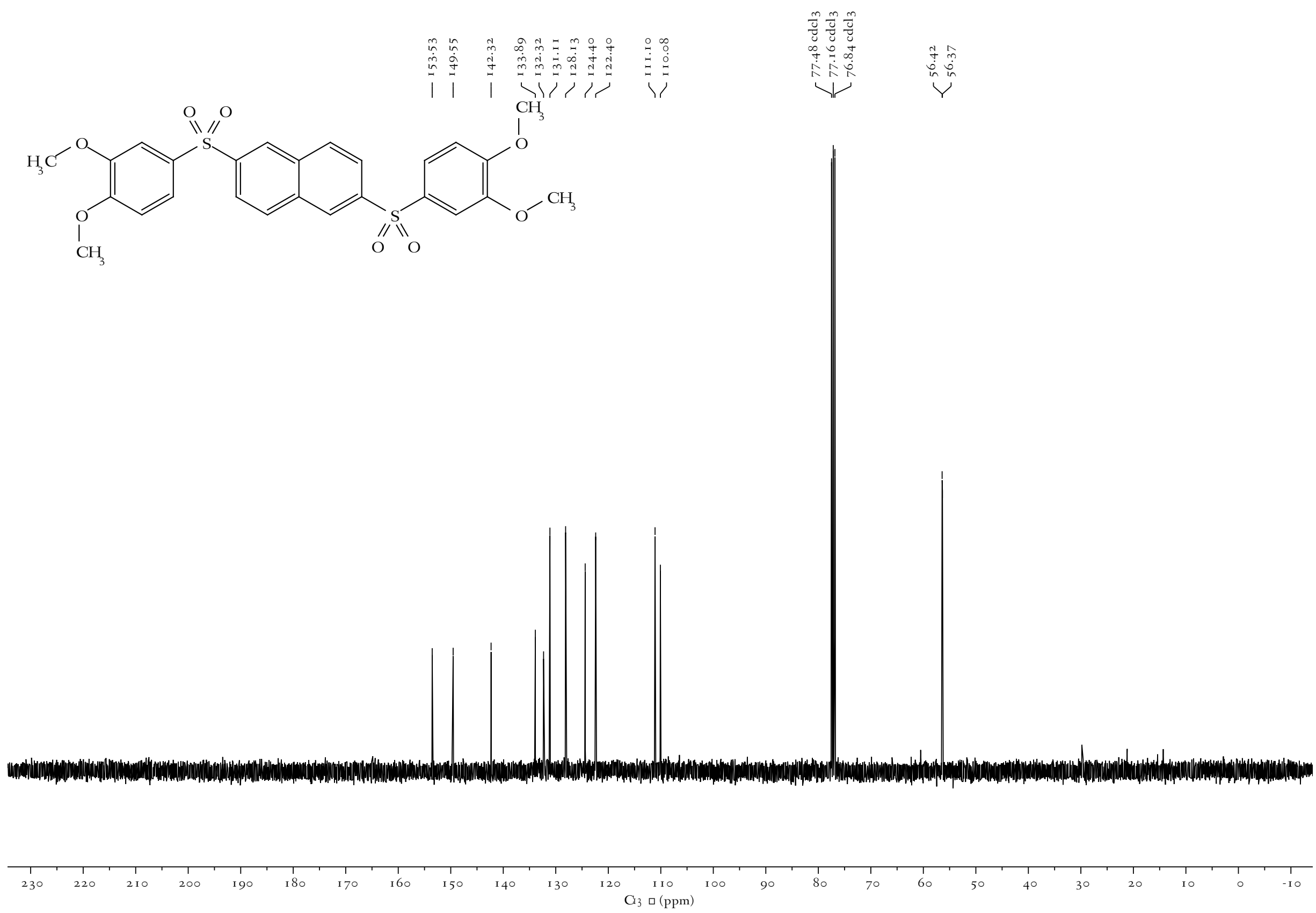

153.53
149.55
142.32
133.89
132.32
131.11
128.13
124.40
122.40
111.10
110.08
77.48 cdcl3
77.16 cdcl3
76.84 cdcl3
56.42
56.37
(ppm)

7c

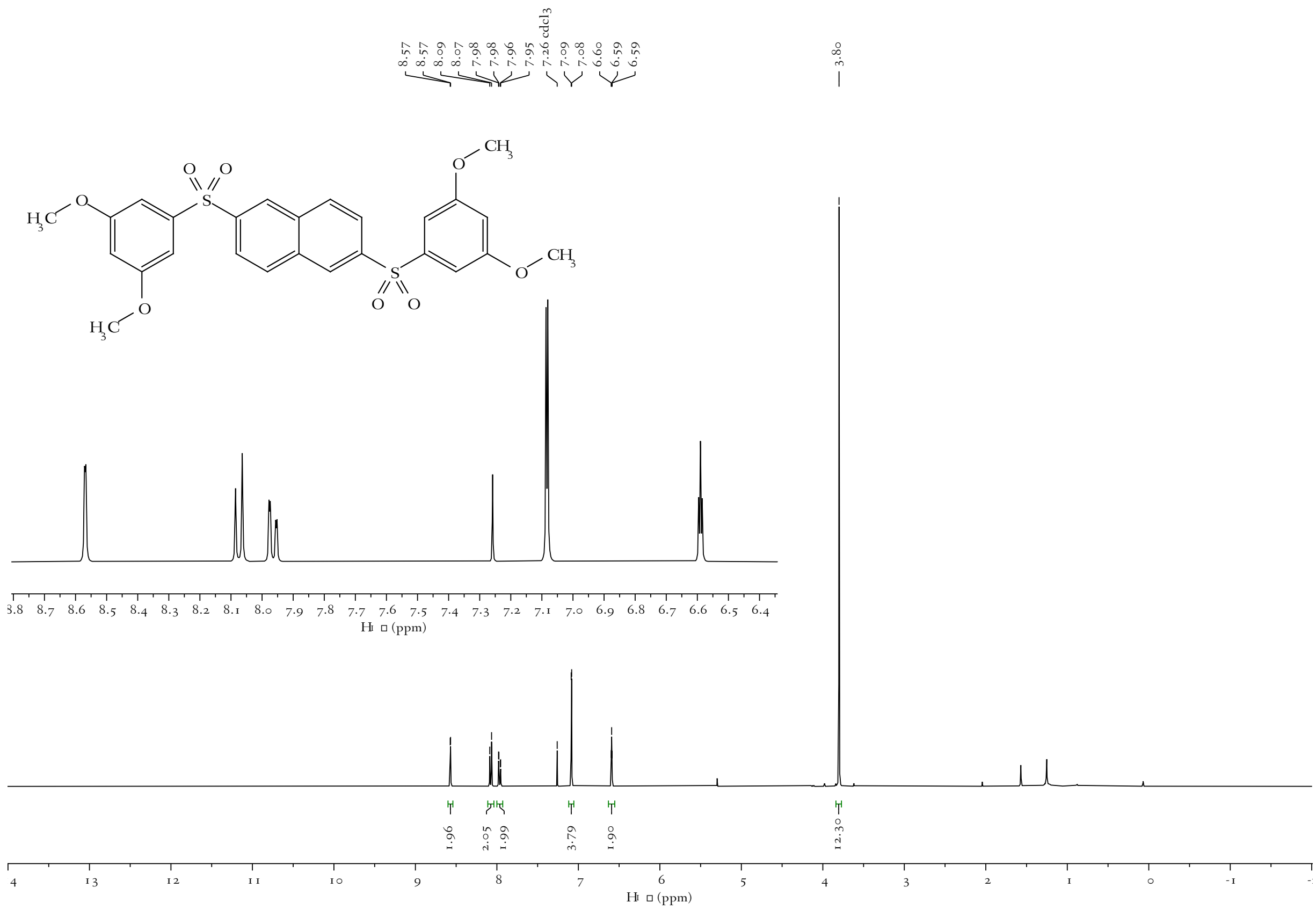
8.57
8.57
8.09
8.07
7.98
7.98
7.96
7.95
7.26 cdcl3
7.09
7.08
6.60
6.59
6.59
3.80
H_3C
O
S
O O
O
CH_3
O
CH_3
H_3C
1.96
2.05
1.99
3.79
1.90
12.30
(ppm)

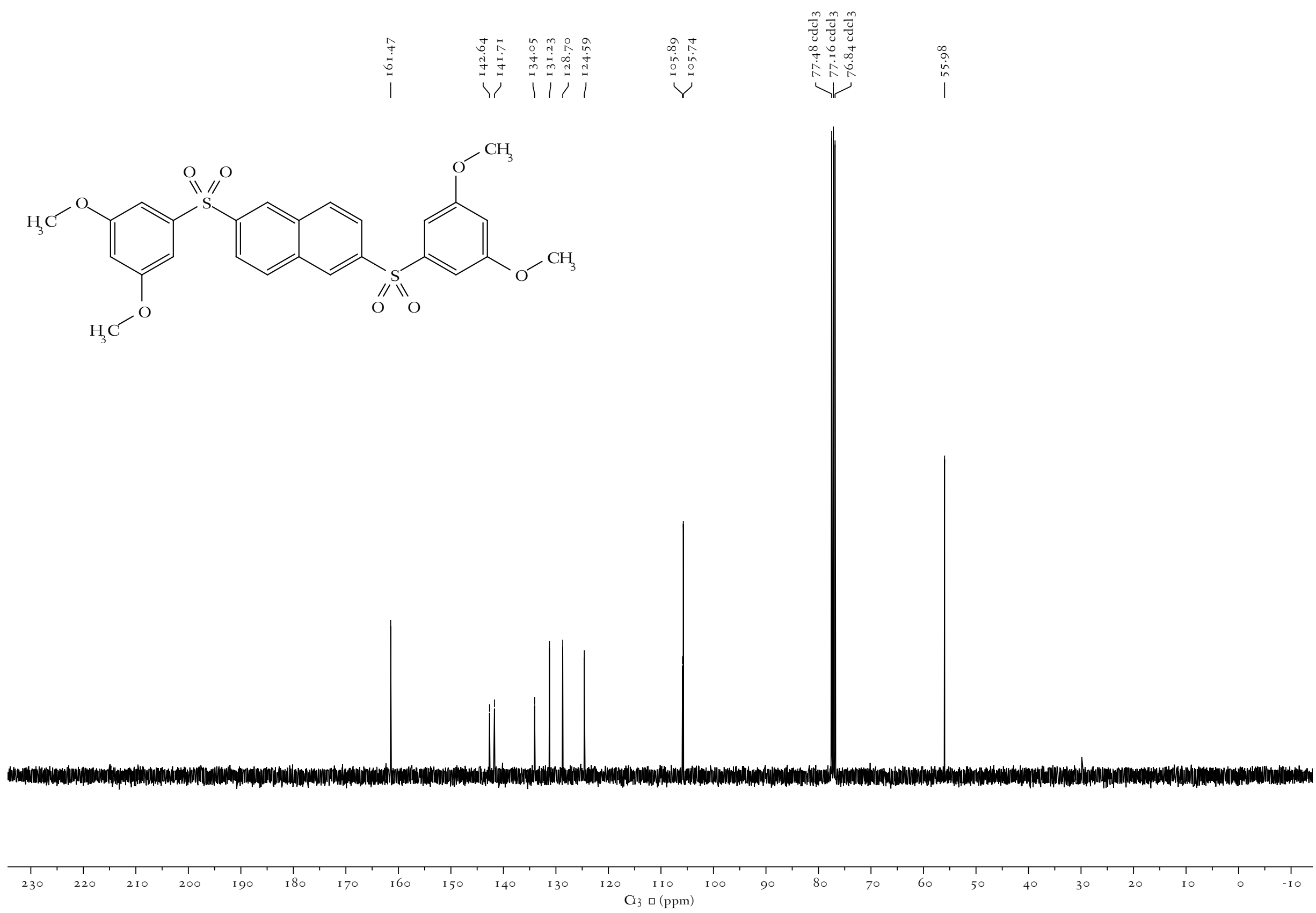
161.47
142.64
141.71
134.05
131.23
128.70
124.59
105.89
105.74
77.48 cdcl3
77.16 cdcl3
76.84 cdcl3
55.98
H_3C
O
S
O O
O
CH_3
O
CH_3
H_3C
(ppm)

7d

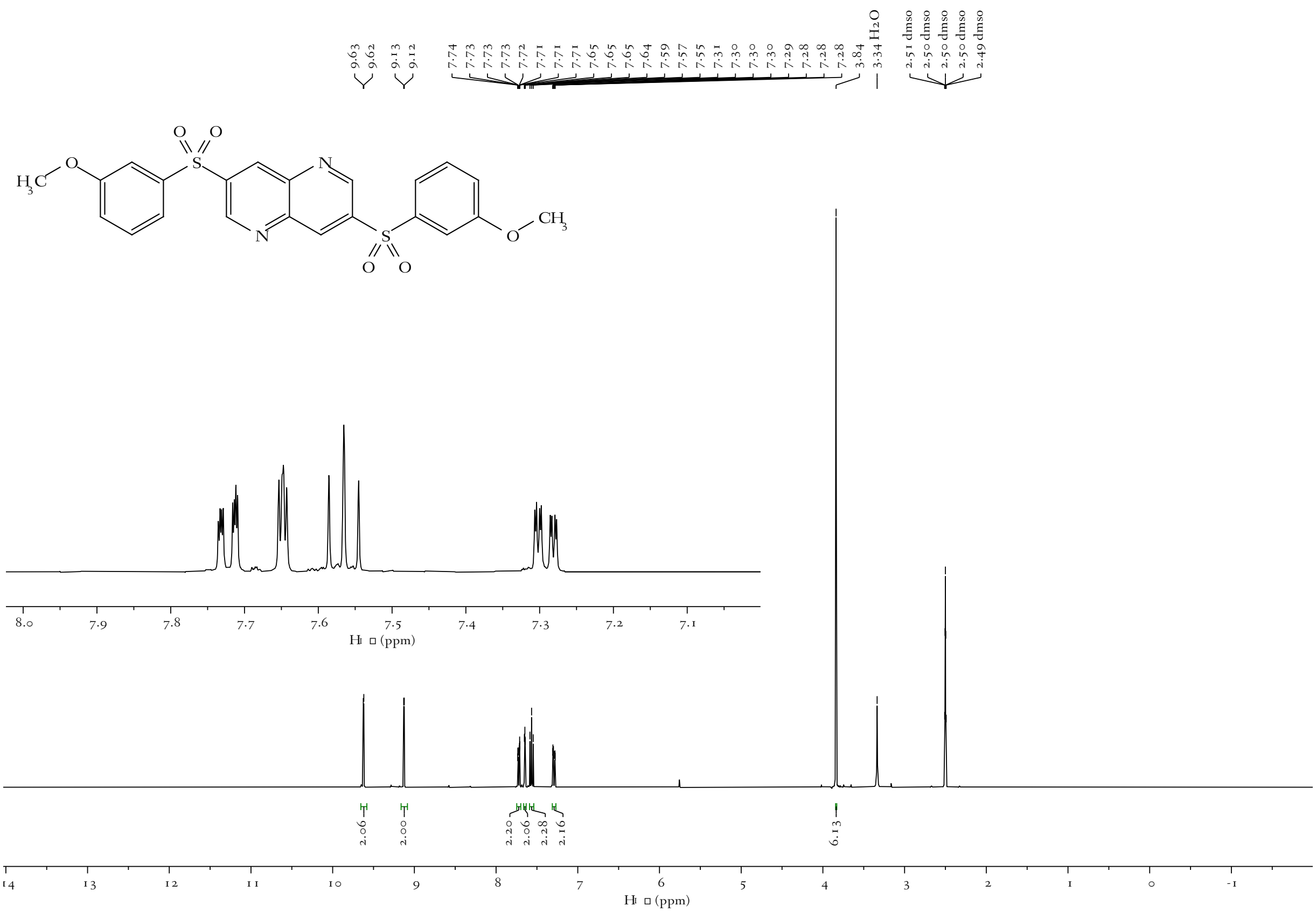

9.63
9.62
9.13
9.12
7.74
7.73
7.73
7.73
7.72
7.71
7.71
7.71
7.65
7.65
7.65
7.64
7.59
7.57
7.55
7.31
7.30
7.30
7.29
7.28
7.28
7.28
3.84
3.34 H2O
2.51 dmso
2.50 dmso
2.50 dmso
2.50 dmso
2.49 dmso
2.06
2.00
2.20
2.06
2.28
2.16
6.13
f1 (ppm)

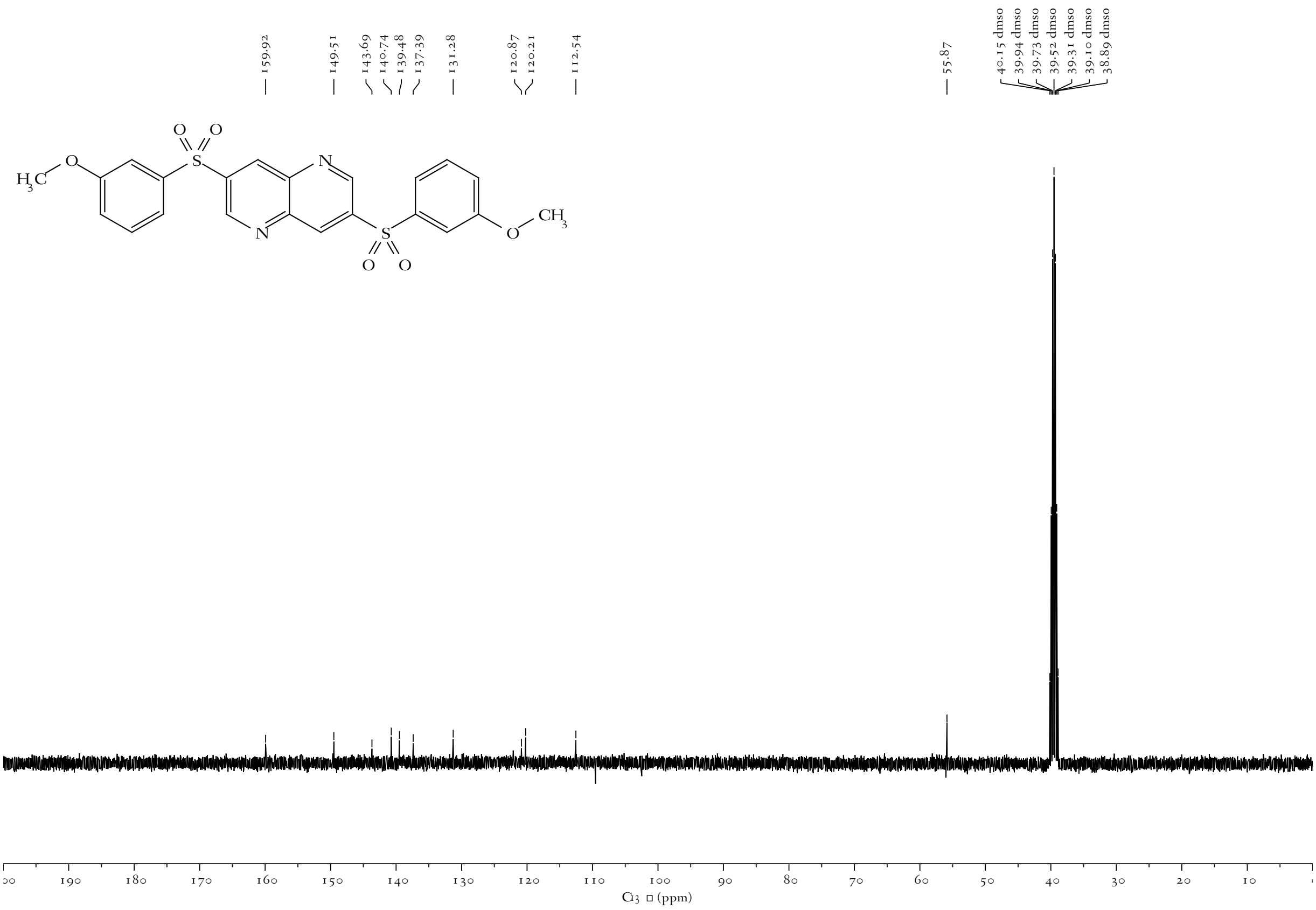

159.92
149.51
143.69
140.74
139.48
137.39
131.28
120.87
120.21
112.54
55.87
40.15 dmso
39.94 dmso
39.73 dmso
39.52 dmso
39.31 dmso
39.10 dmso
38.89 dmso
f1 (ppm)

7e

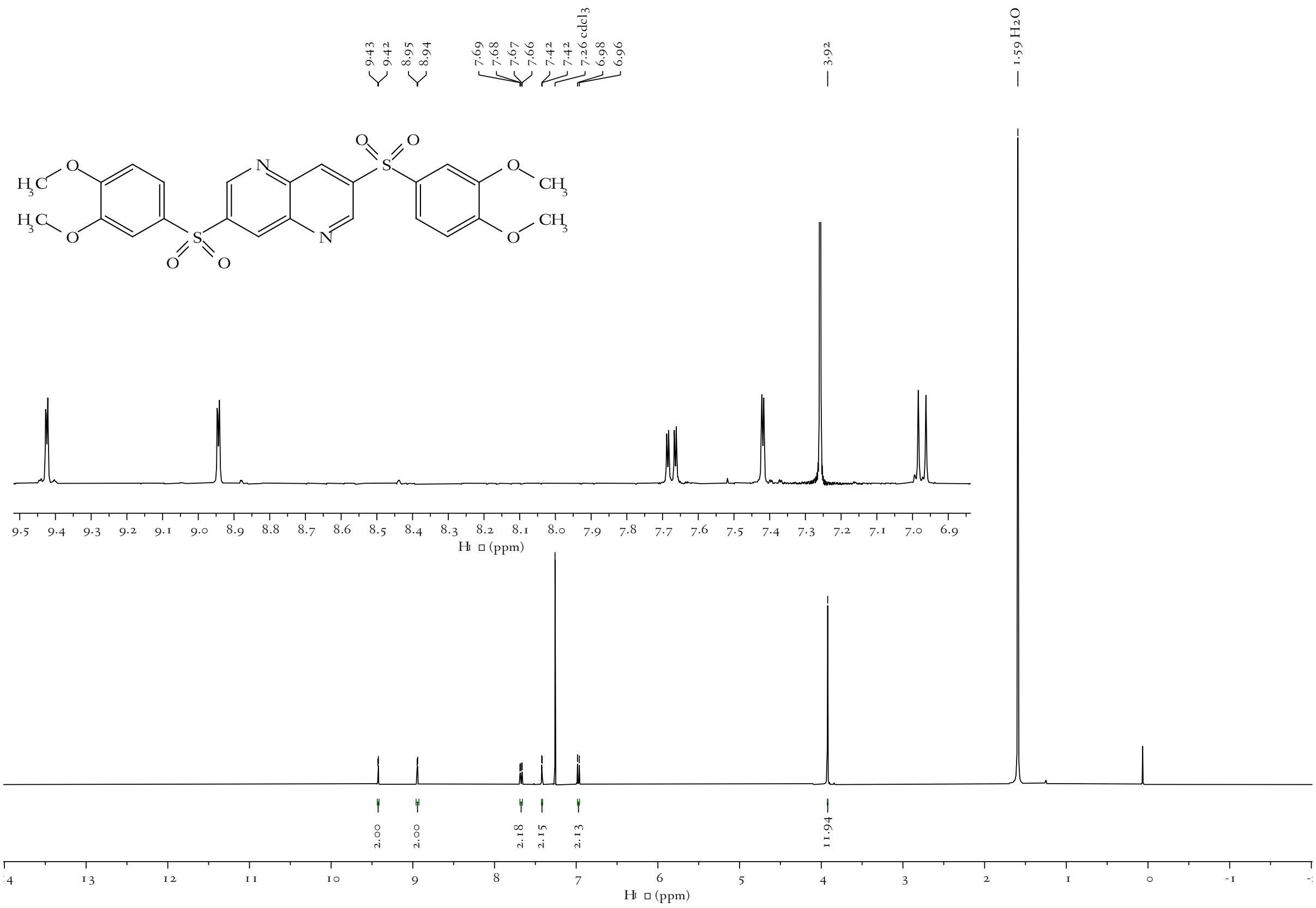

9.43
9.42
8.95
8.94
7.69
7.68
7.67
7.66
7.42
7.42
7.26 cdcl3
6.98
6.96
3.92
1.59 H2O
2.00
2.00
2.18
2.15
2.13
11.94
(ppm)

7f

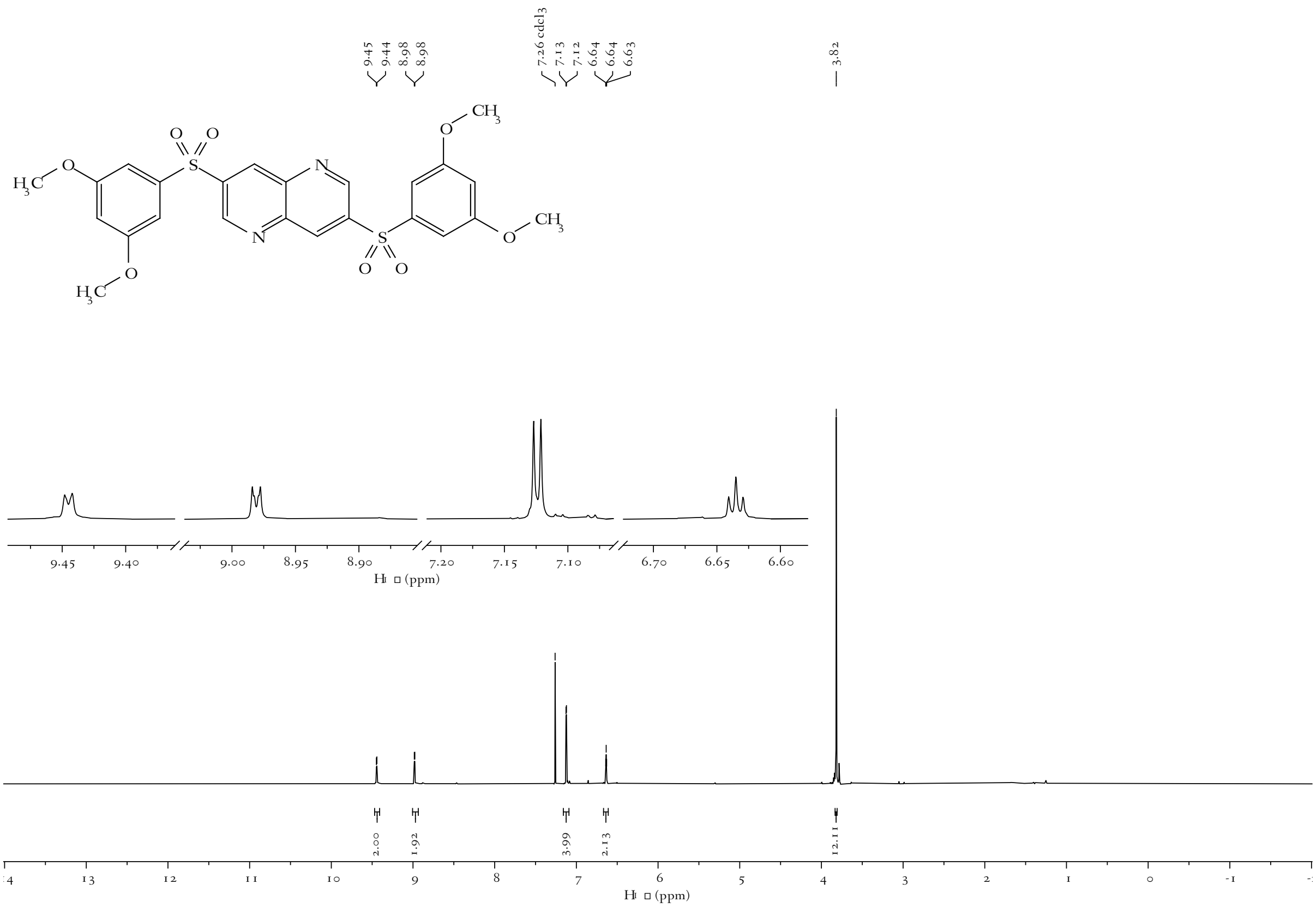

9.45
9.44
8.98
8.98
7.26 cdcl3
7.13
7.12
6.64
6.64
6.63
3.82
2.00
1.92
3.99
2.13
12.11
(ppm)

7g

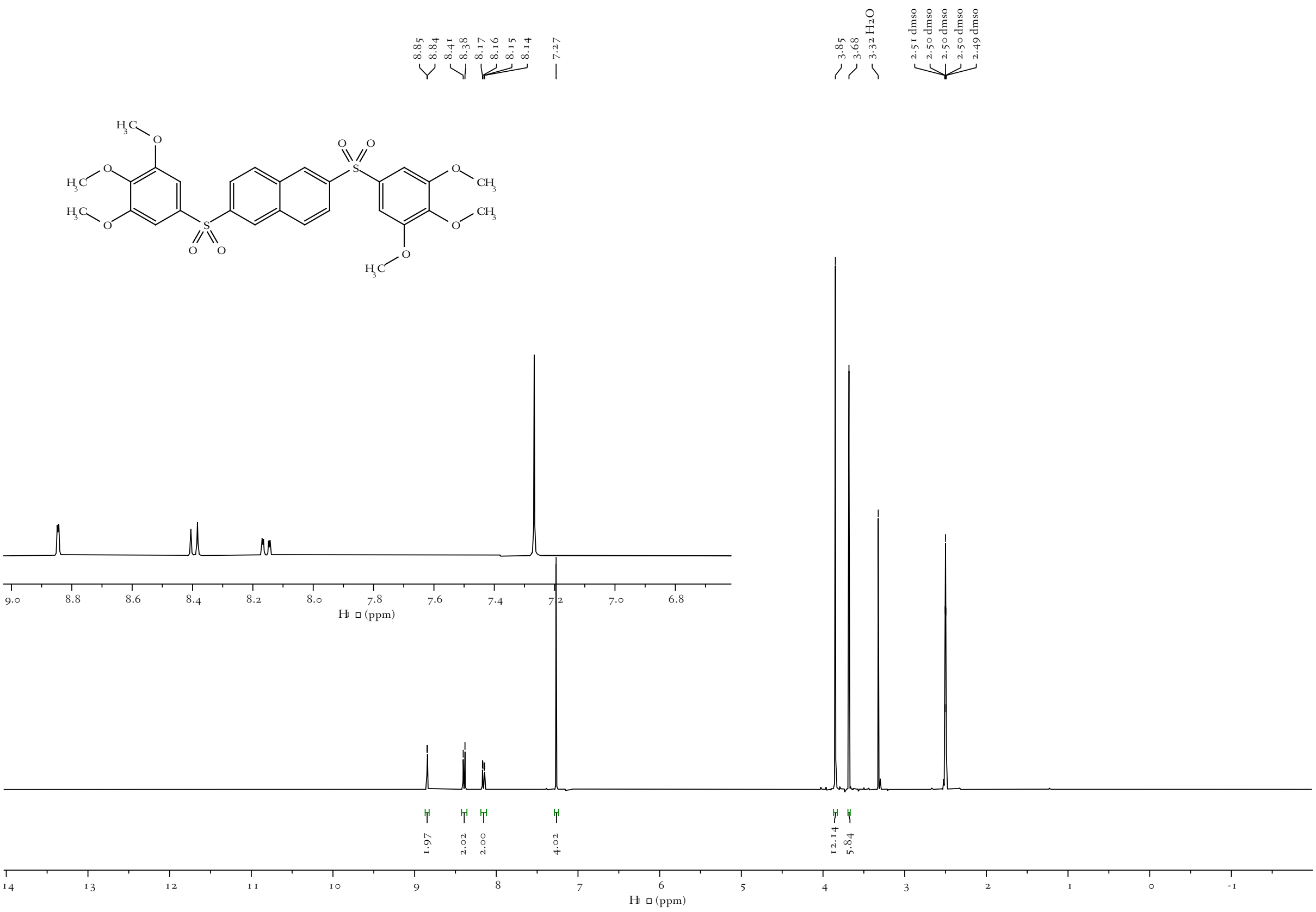
8.85
8.84
8.41
8.38
8.17
8.16
8.15
8.14
7.27
3.85
3.68
3.32 H2O
2.51 dmso
2.50 dmso
2.50 dmso
2.50 dmso
2.49 dmso
1.97
2.02
2.00
4.02
12.14
5.84
(ppm)

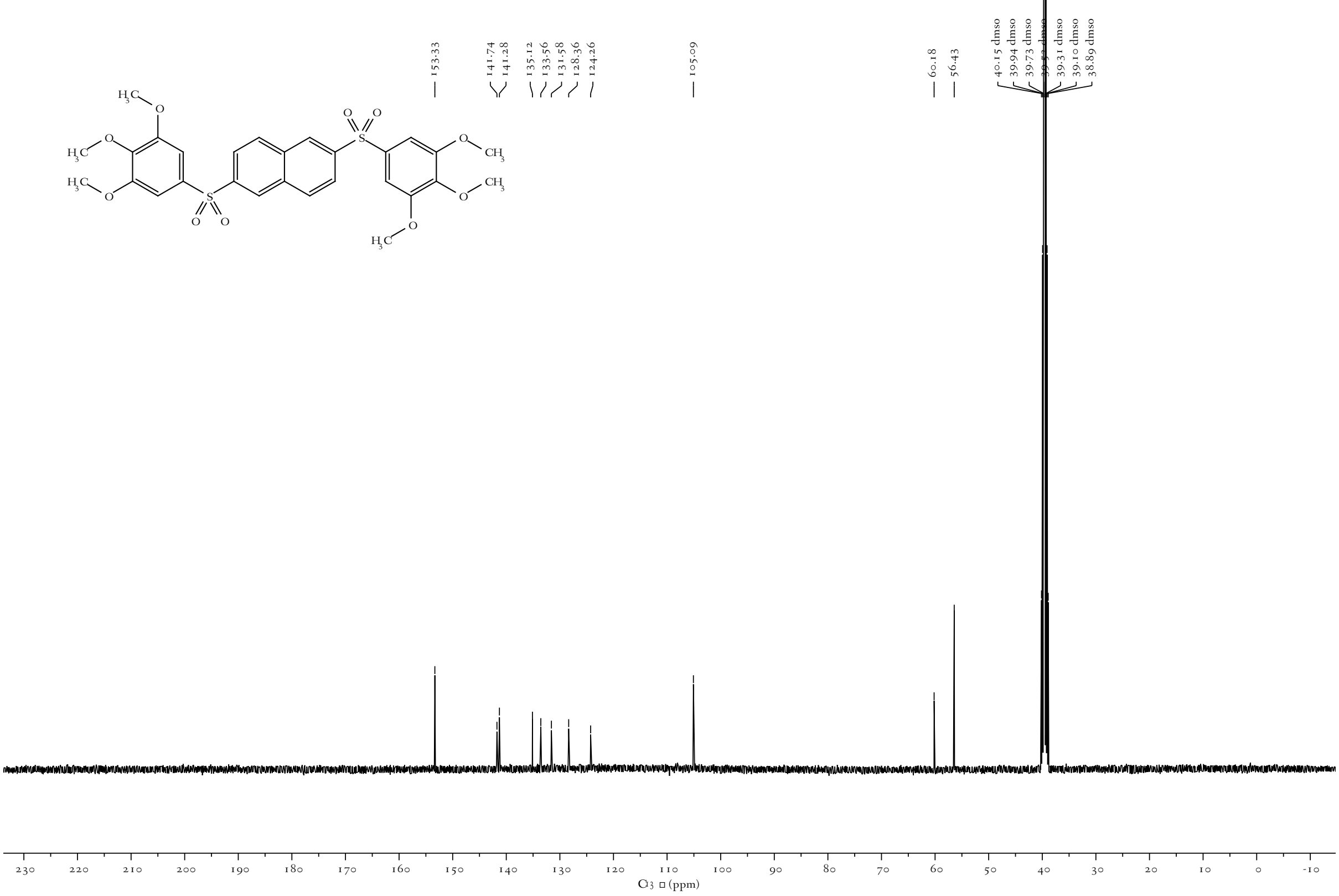
153.33
141.74
141.28
135.12
133.56
131.58
128.36
124.26
105.09
60.18
56.43
40.15 dmso
39.94 dmso
39.73 dmso
39.31 dmso
39.10 dmso
38.89 dmso
(ppm)

7h

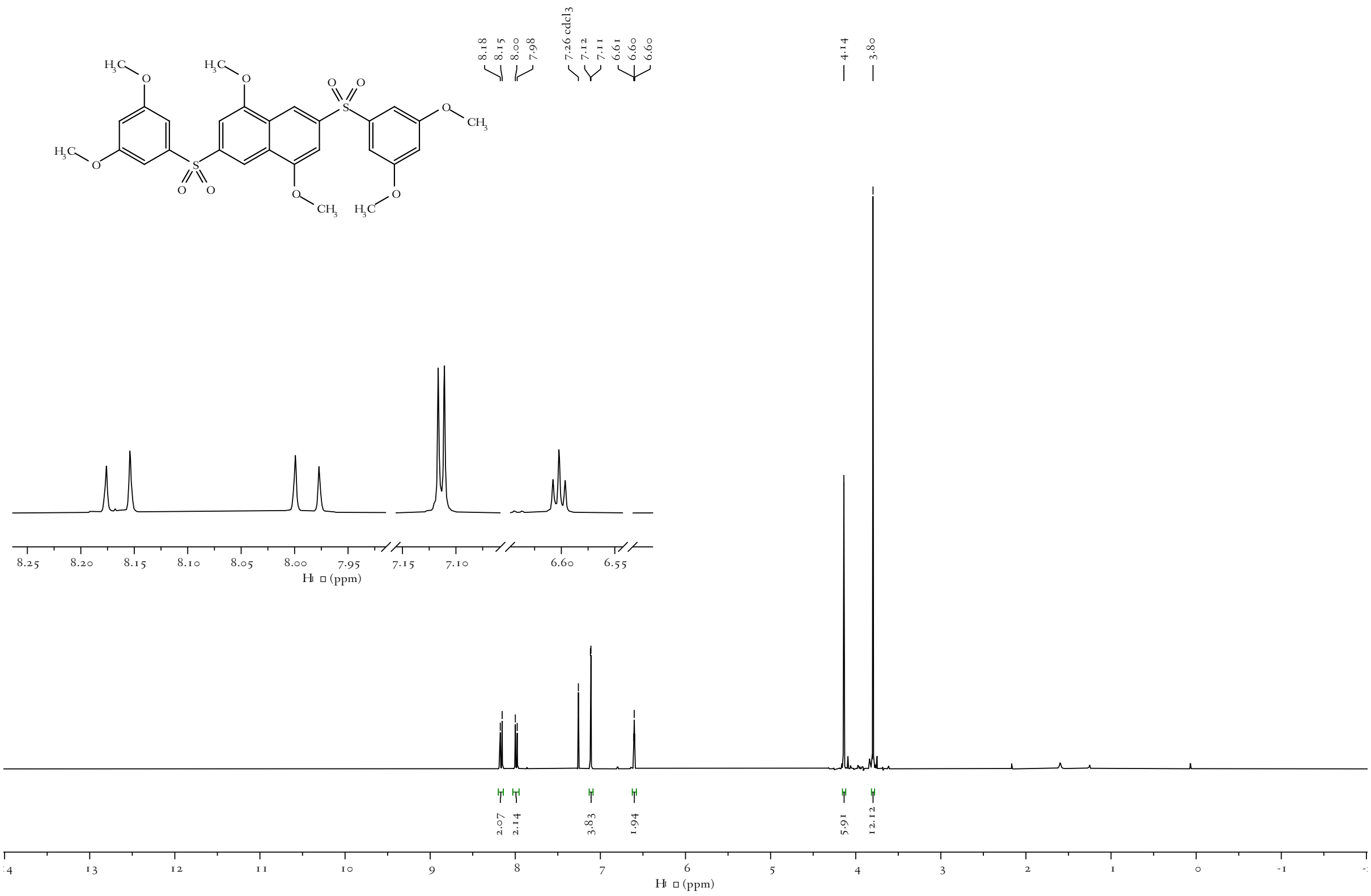

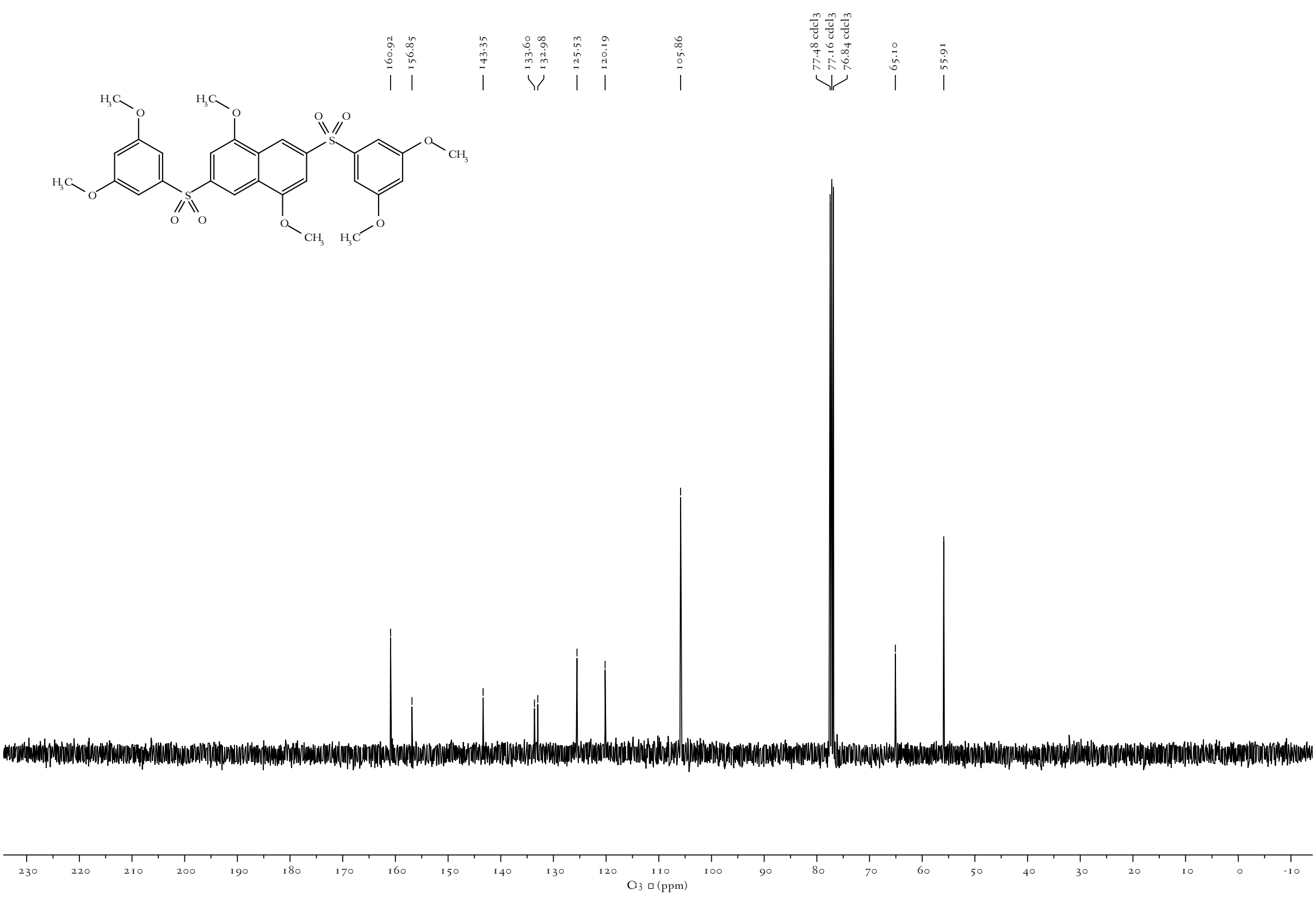

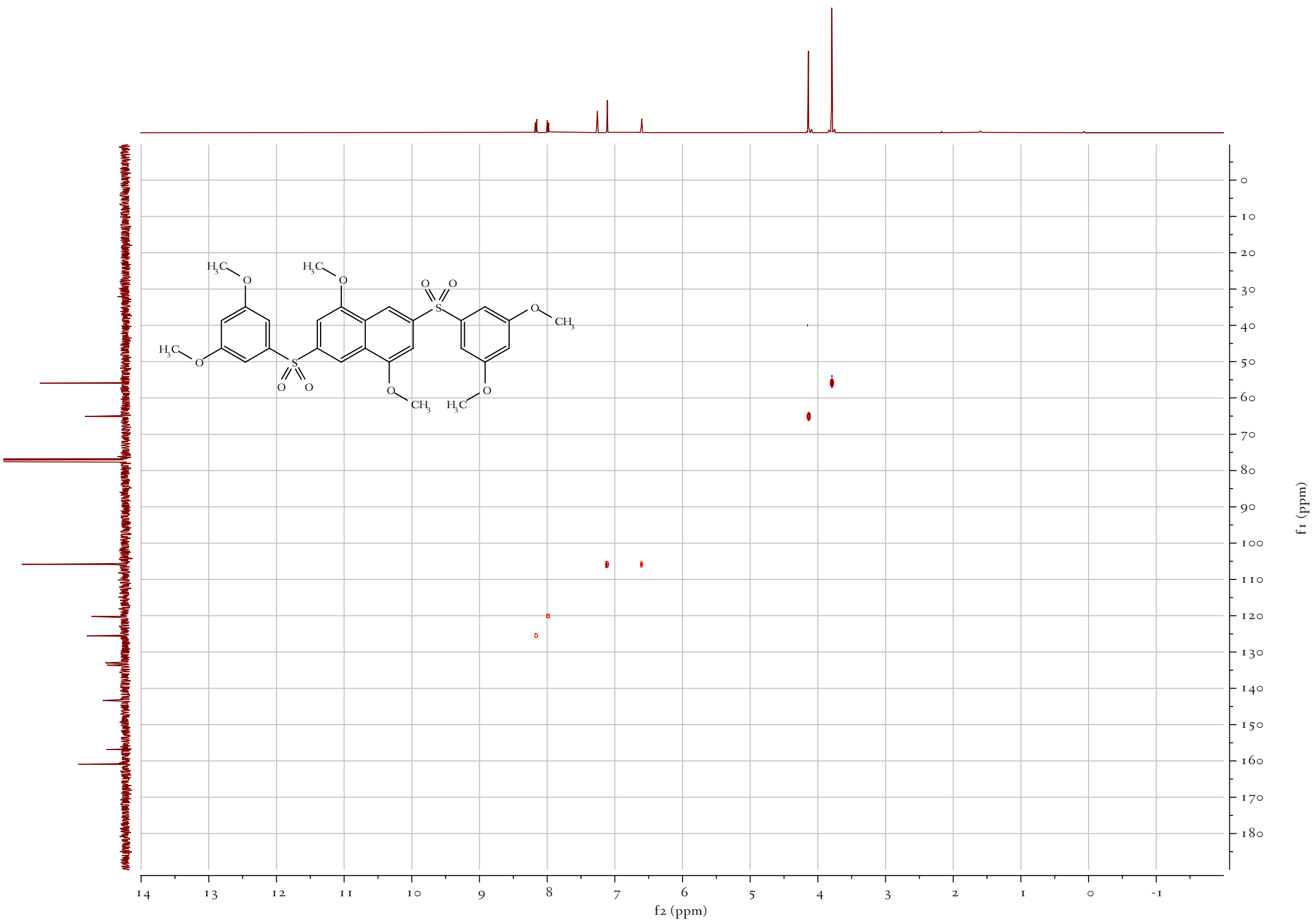
f2 (ppm)
f1 (ppm)

8a

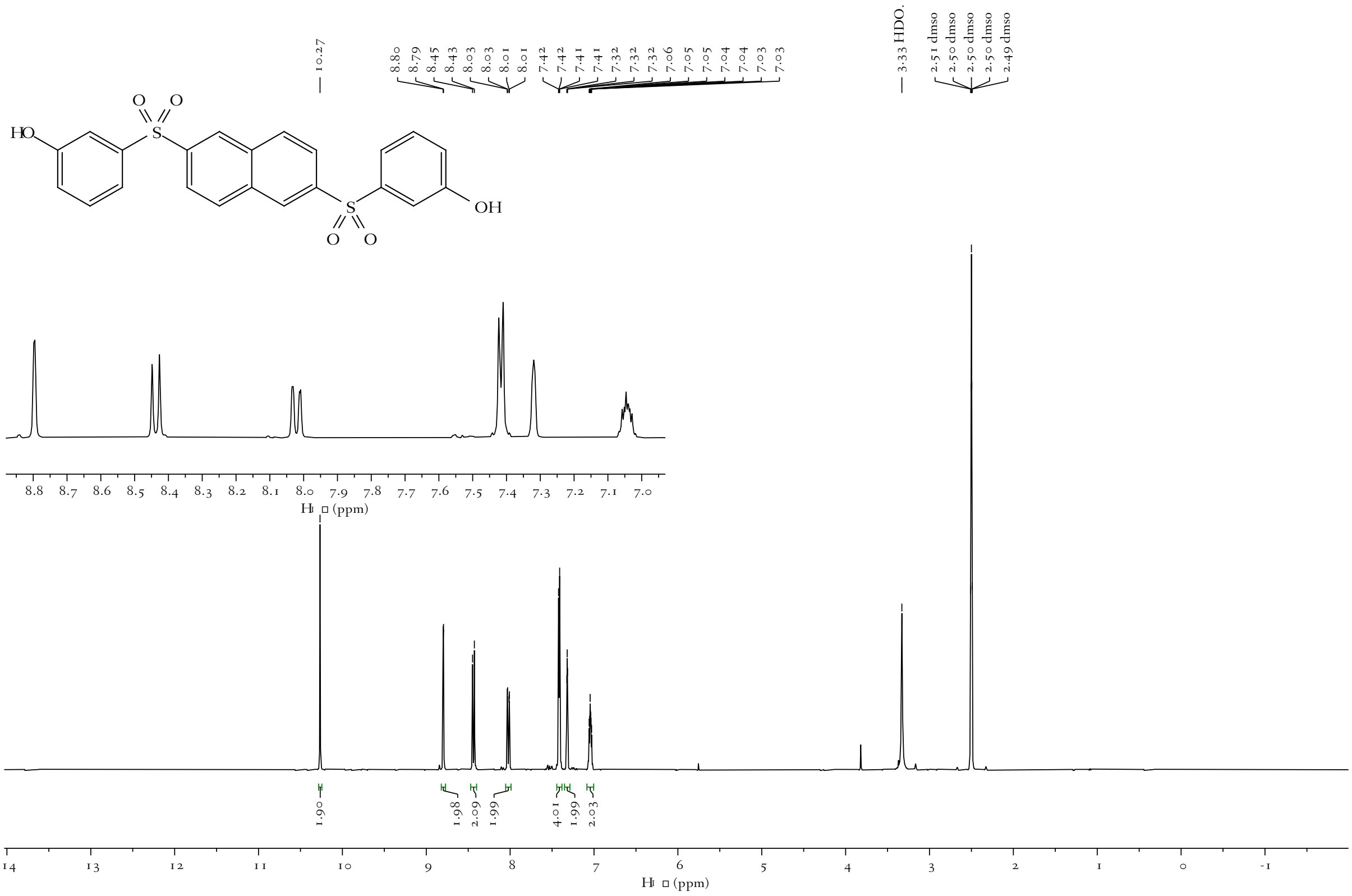

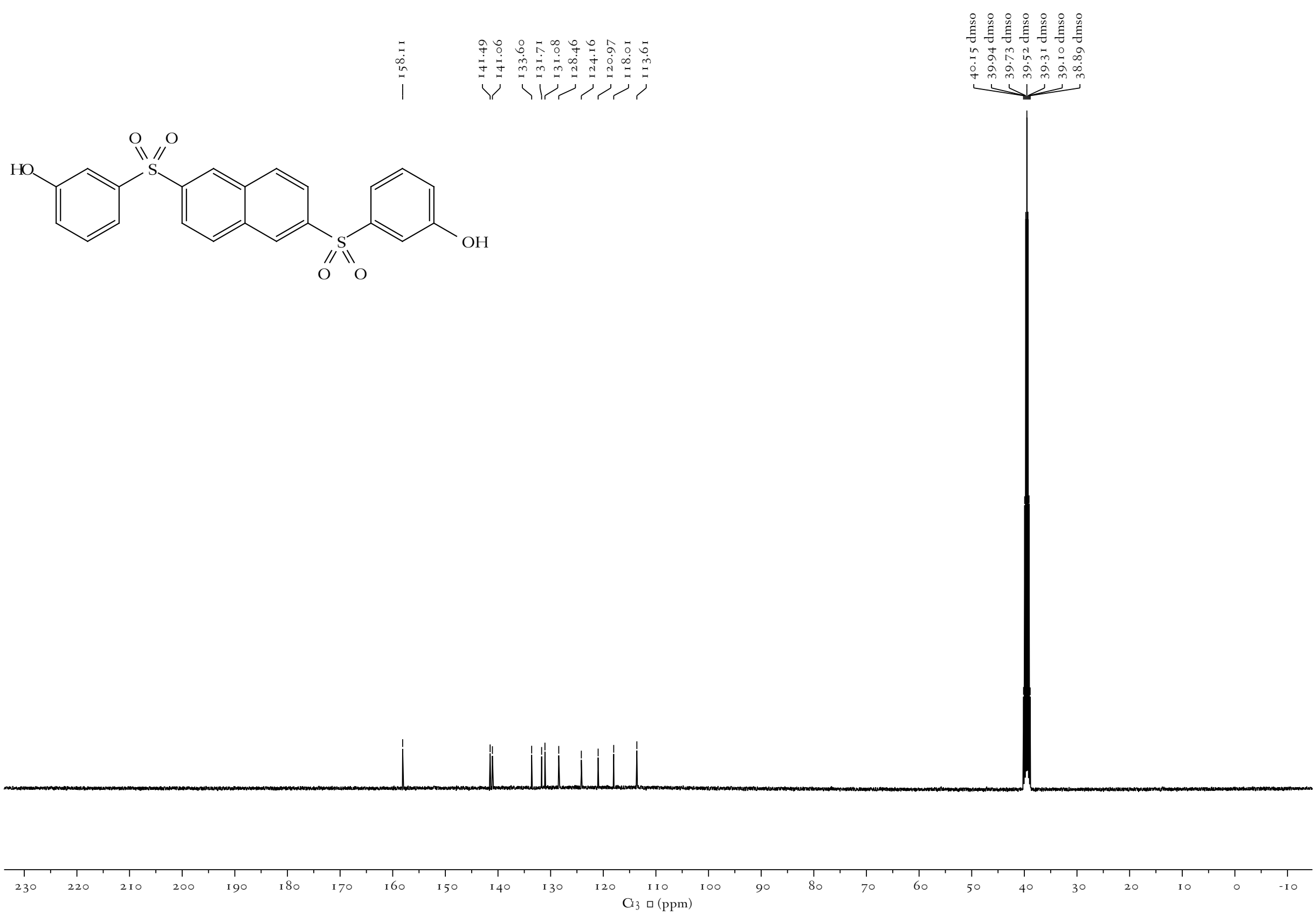

8b

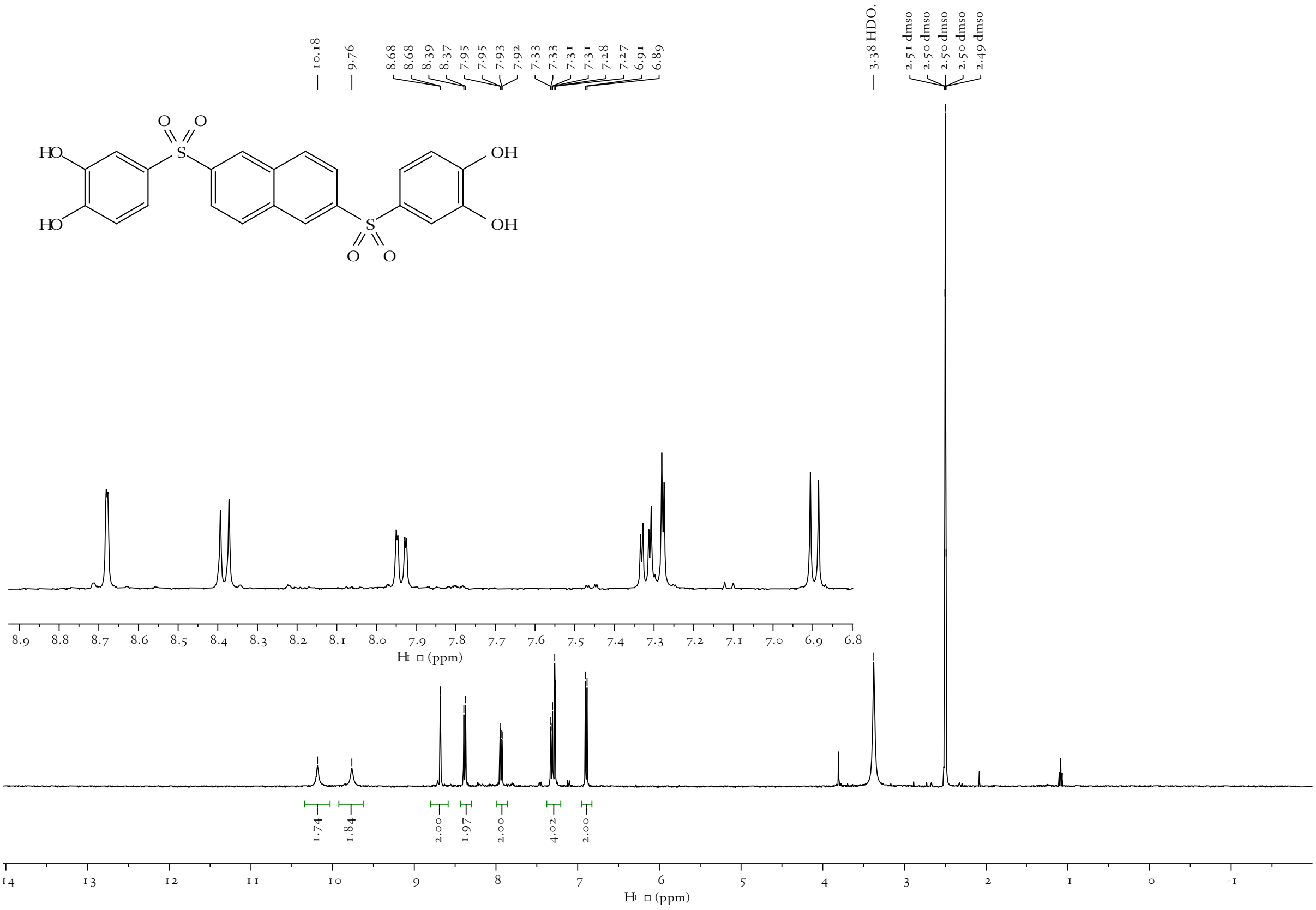

10.18
9.76
8.68 8.68 8.39 8.37 7.95 7.95 7.93 7.92 7.33 7.33 7.31 7.31 7.28 7.27 6.91 6.89
3.38 HDO.
2.51 dmso 2.50 dmso 2.50 dmso 2.50 dmso 2.49 dmso
HO
HO
OH
OH
1.74 1.84 2.00 1.97 2.00 4.02 2.00
f1 (ppm)

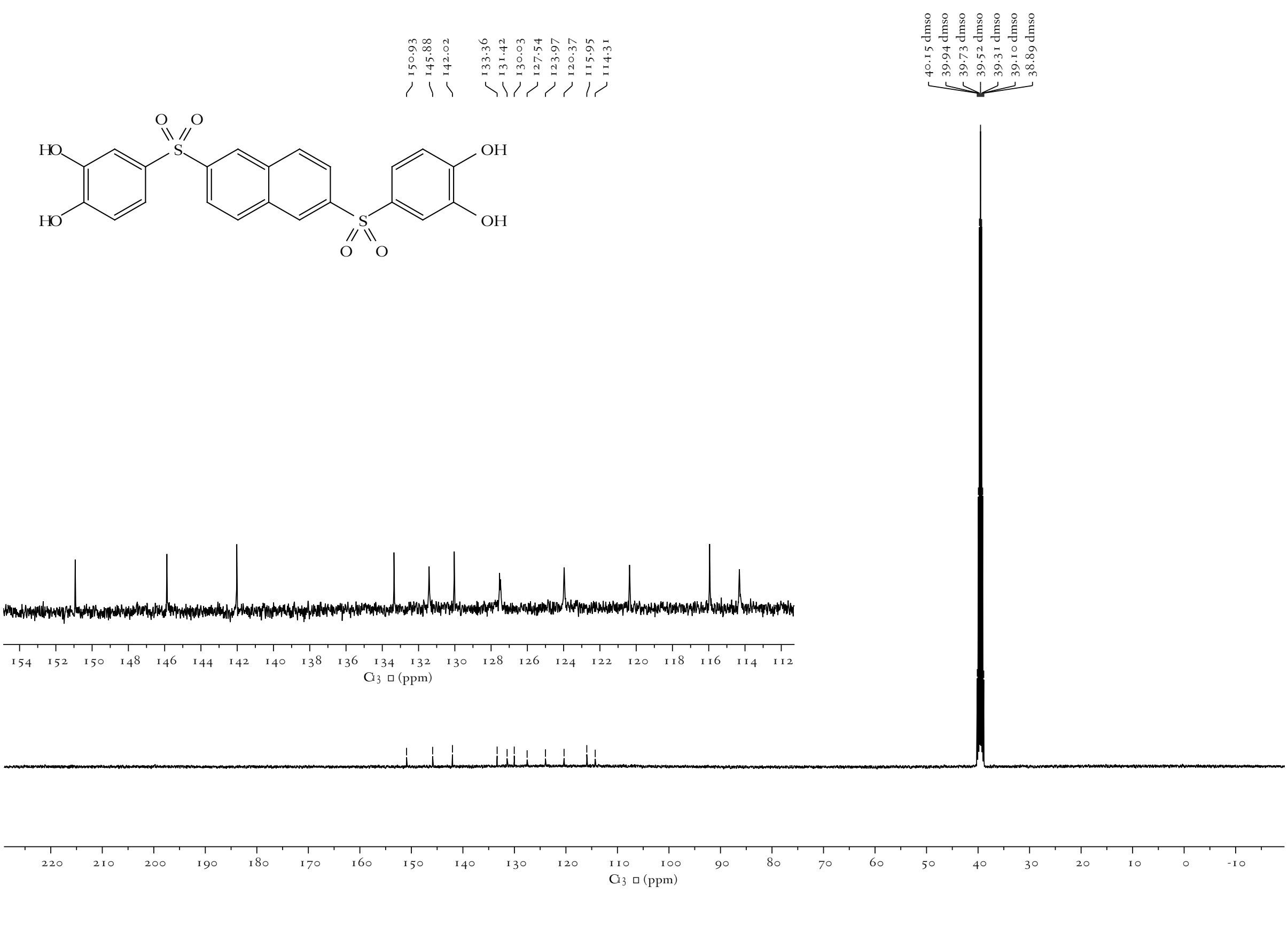

150.93 145.88 142.02 133.36 131.42 130.03 127.54 123.97 120.37 115.95 114.31
40.15 dmso 39.94 dmso 39.73 dmso 39.52 dmso 39.31 dmso 39.10 dmso 38.89 dmso
HO
HO
OH
OH
f1 (ppm)

8c

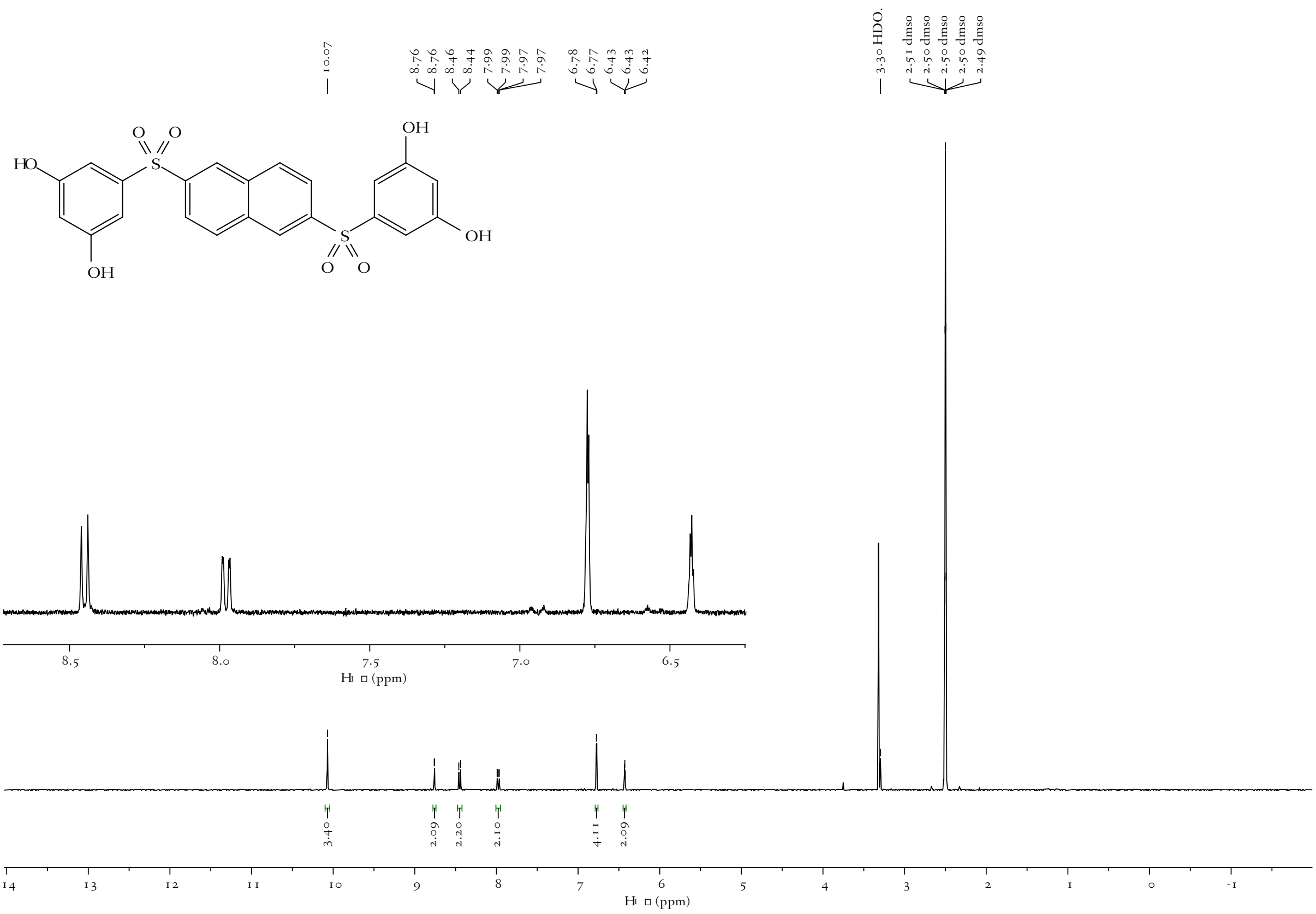
10.07
8.76
8.76
8.46
8.44
7.99
7.99
7.97
7.97
6.78
6.77
6.43
6.43
6.42
3.30 HDO.
2.51 dmso
2.50 dmso
2.50 dmso
2.50 dmso
2.49 dmso
3.40
2.09
2.20
2.10
4.11
2.09
H ppm

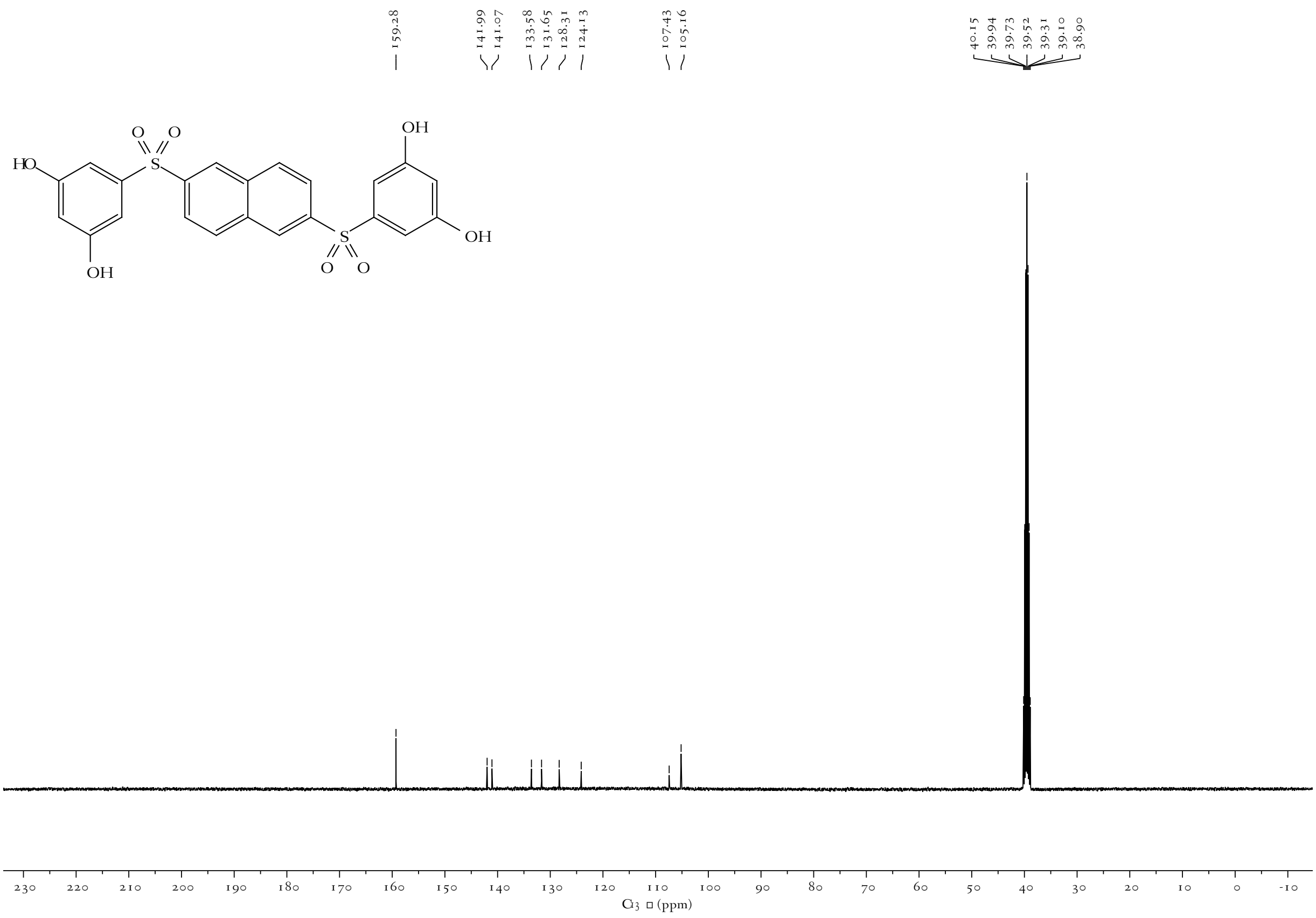
159.28
141.99
141.07
133.58
131.65
128.31
124.13
107.43
105.16
40.15
39.94
39.73
39.52
39.31
39.10
38.90
C3 ppm

8d

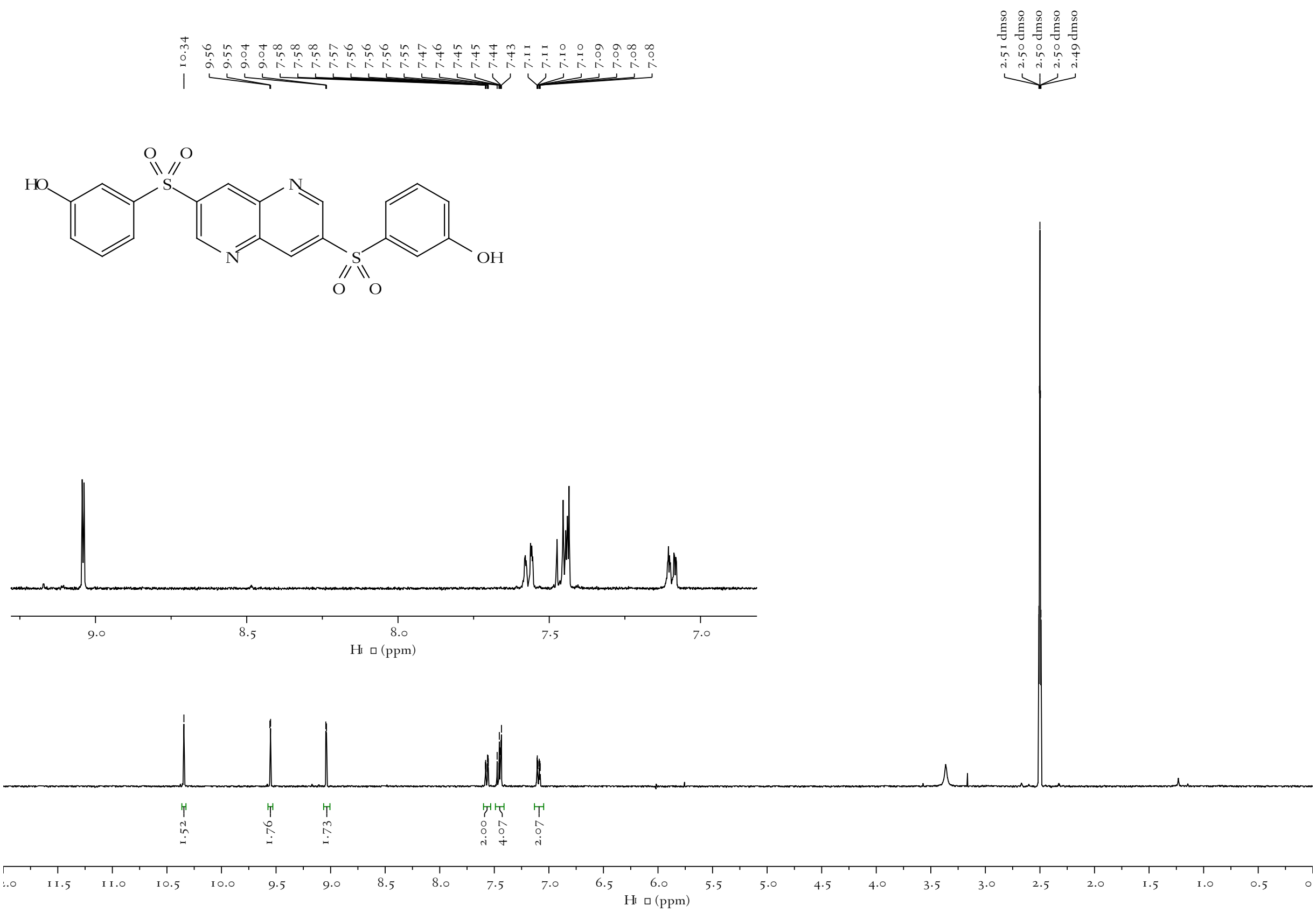

10.34
9.56
9.55
9.04
9.04
7.58
7.58
7.58
7.57
7.56
7.56
7.56
7.55
7.47
7.46
7.45
7.45
7.44
7.43
7.11
7.11
7.10
7.10
7.09
7.09
7.08
7.08
2.51 dmso
2.50 dmso
2.50 dmso
2.50 dmso
2.49 dmso
HO
S
O
O
N
N
S
O
O
OH
1.52
1.76
1.73
2.00
4.07
2.07

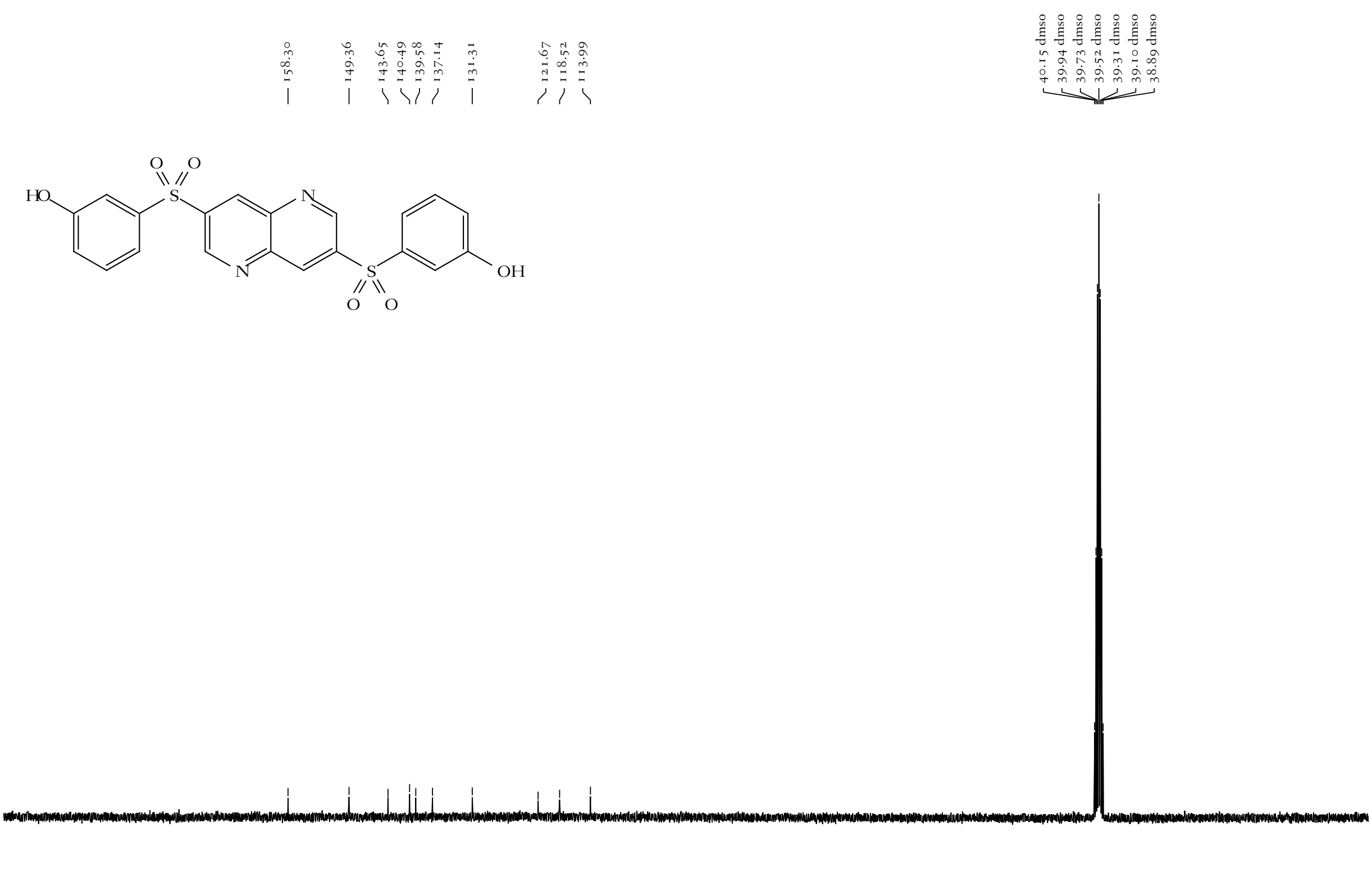

158.30
149.36
143.65
140.49
139.58
137.14
131.31
121.67
118.52
113.99
40.15 dmso
39.94 dmso
39.73 dmso
39.52 dmso
39.31 dmso
39.10 dmso
38.89 dmso
HO
S
O
O
N
N
S
O
O
OH

8e

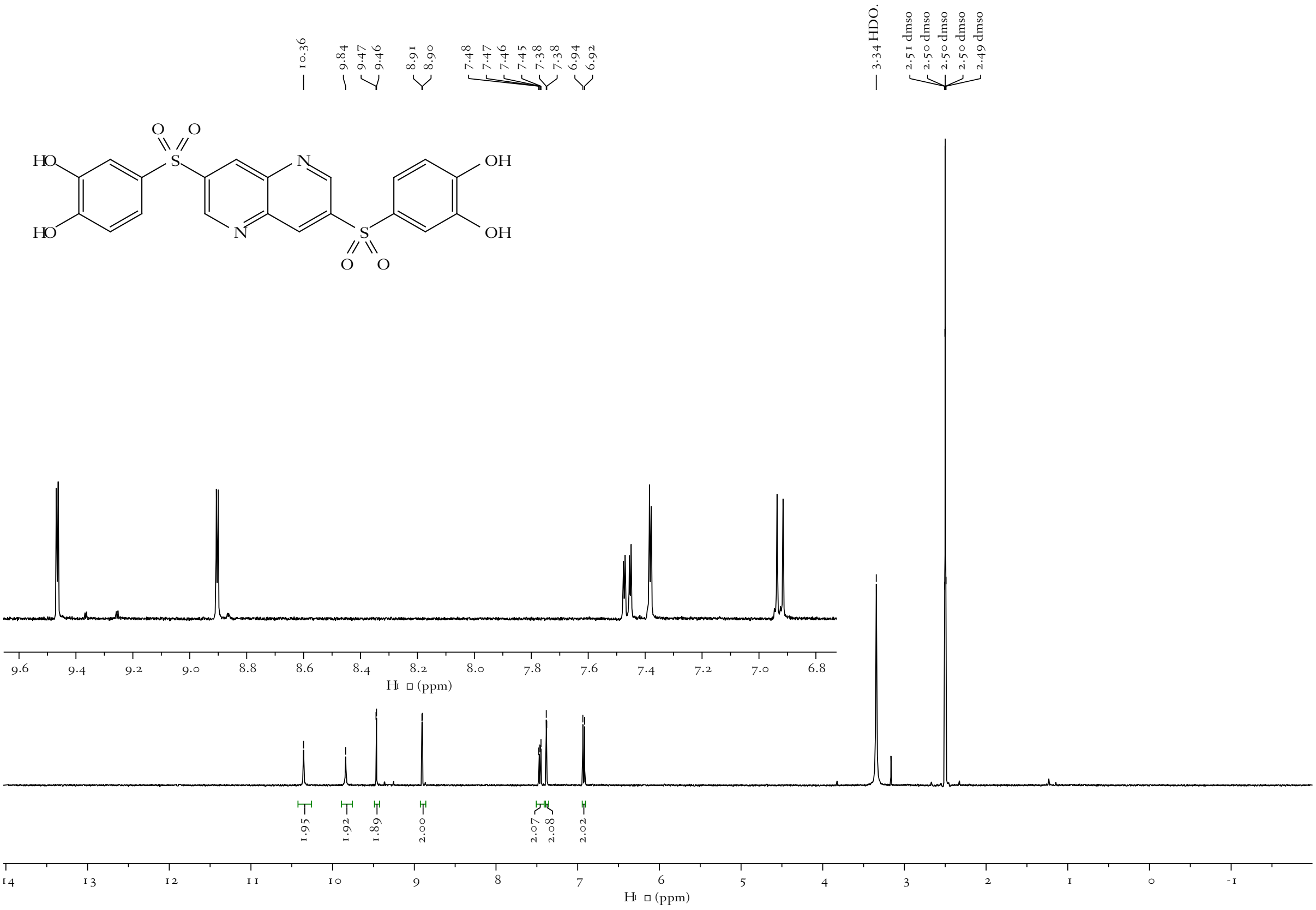
10.36
9.84
9.47
9.46
8.91
8.90
7.48
7.47
7.46
7.45
7.38
7.38
6.94
6.92
3.34 HDO.
2.51 dmso
2.50 dmso
2.50 dmso
2.50 dmso
2.49 dmso
HO
HO
OH
OH
N
N
S
O
1.95
1.92
1.89
2.00
2.07
2.08
2.02
(ppm)

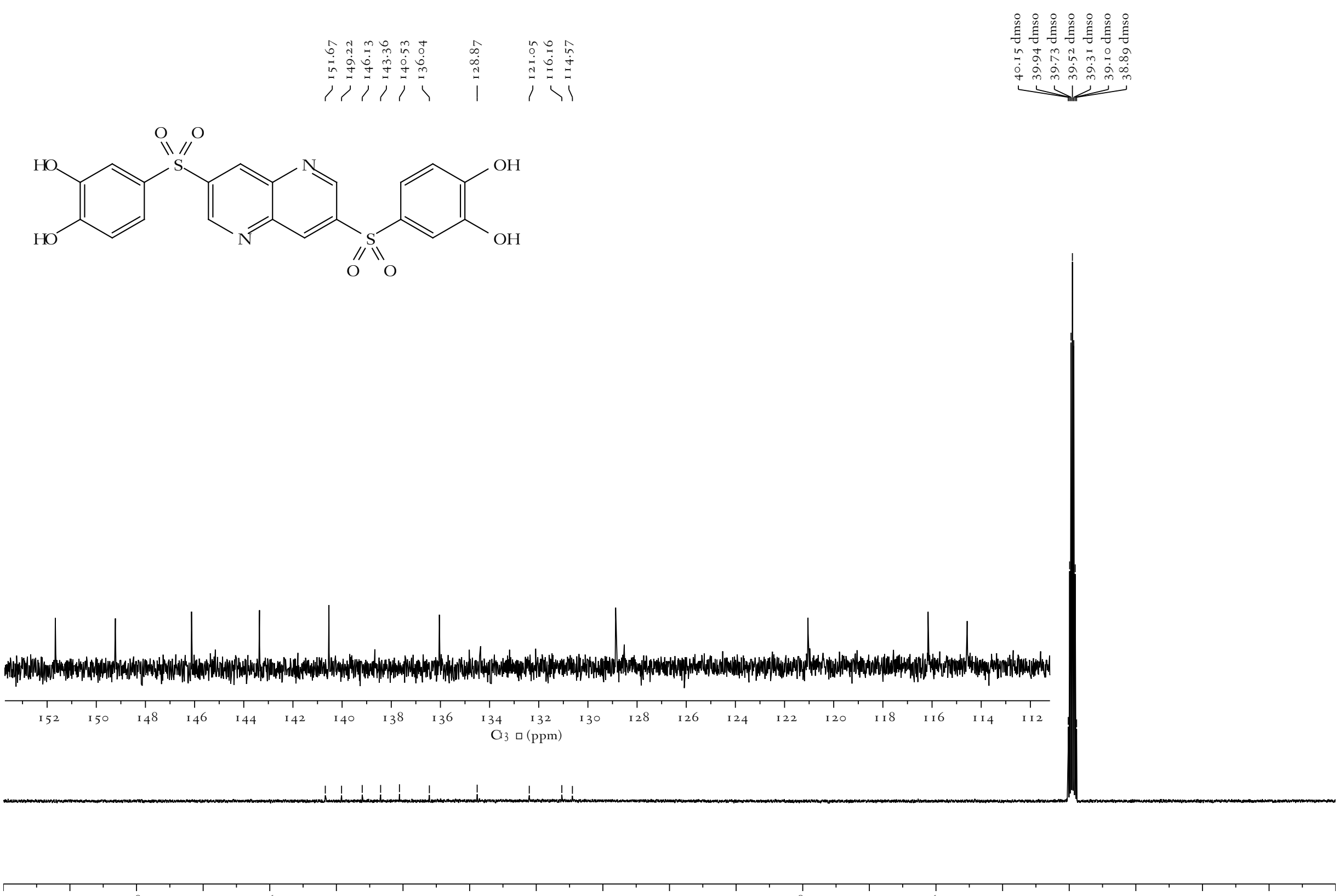
151.67
149.22
146.13
143.36
140.53
136.04
128.87
121.05
116.16
114.57
40.15 dmso
39.94 dmso
39.73 dmso
39.52 dmso
39.31 dmso
39.10 dmso
38.89 dmso
HO
HO
OH
OH
N
N
S
O
(ppm)

8f

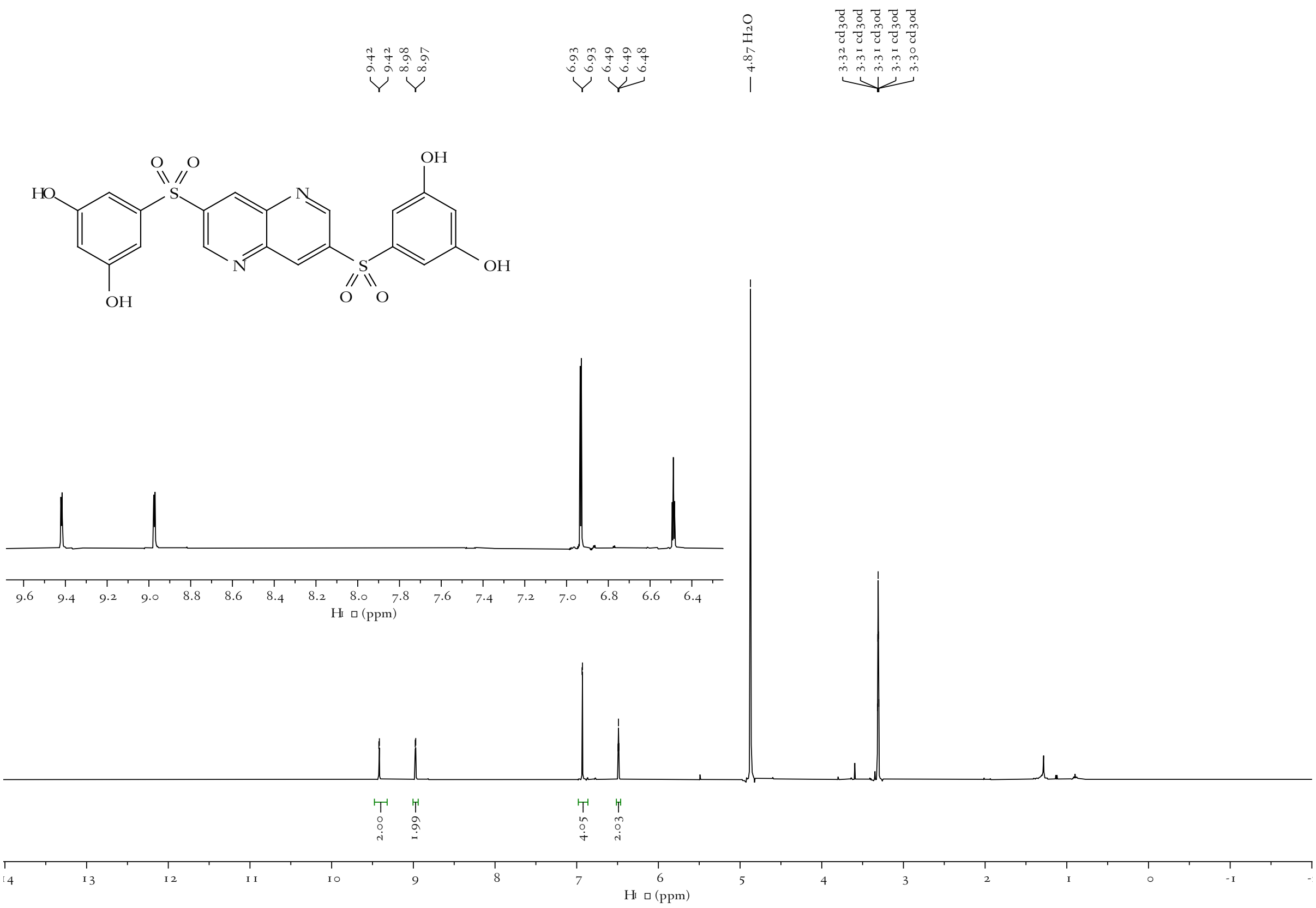

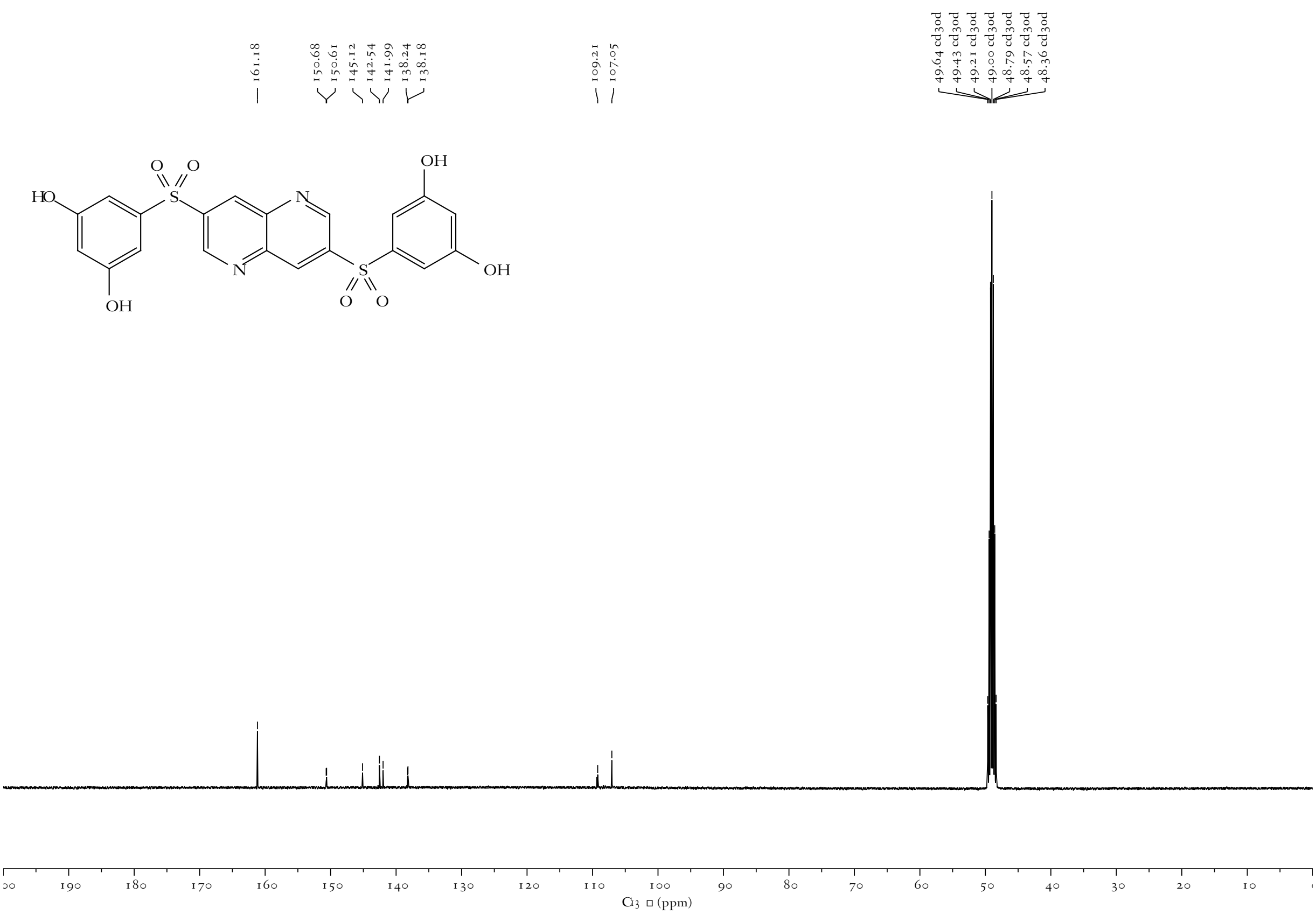

8g

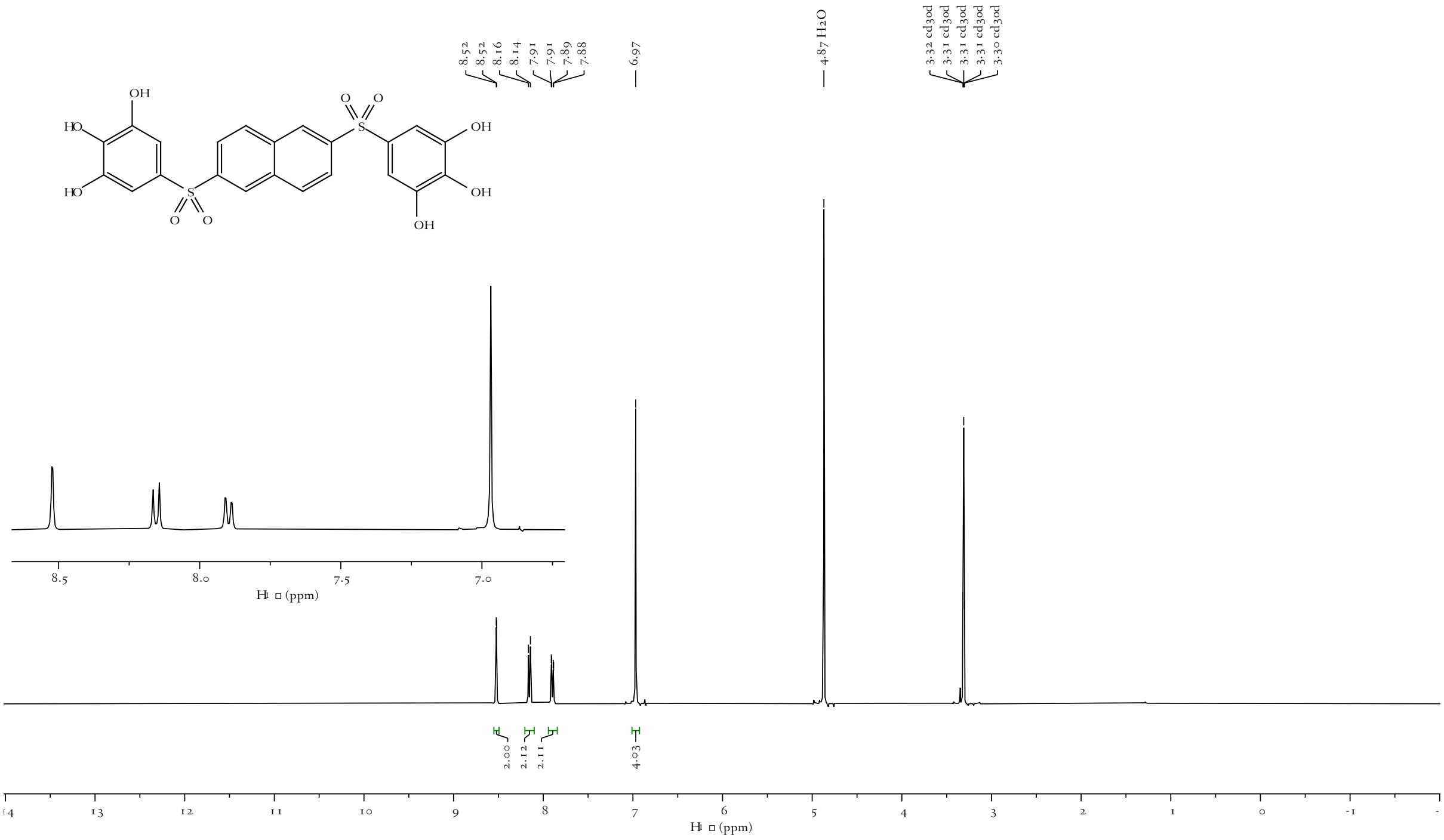

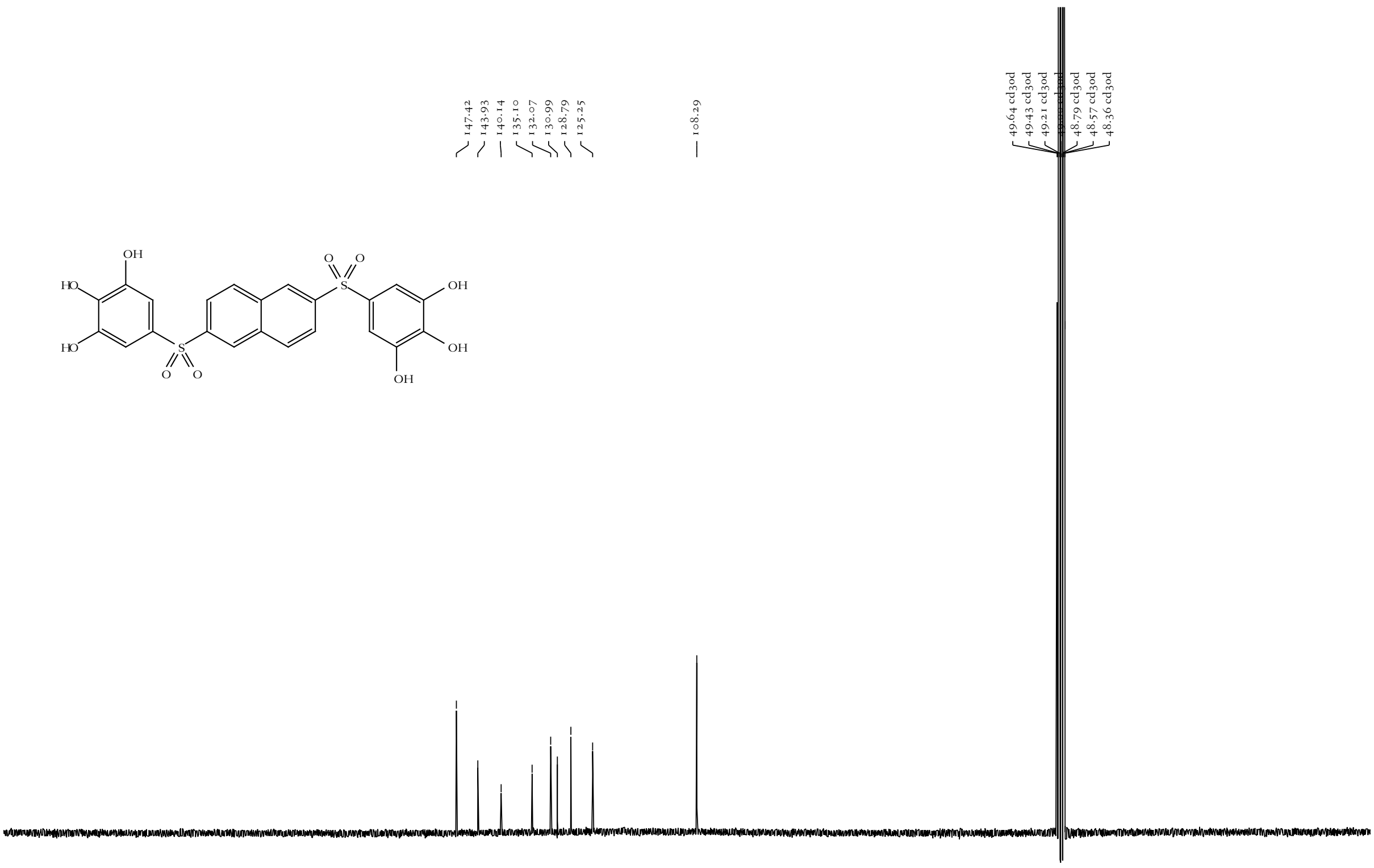

8h

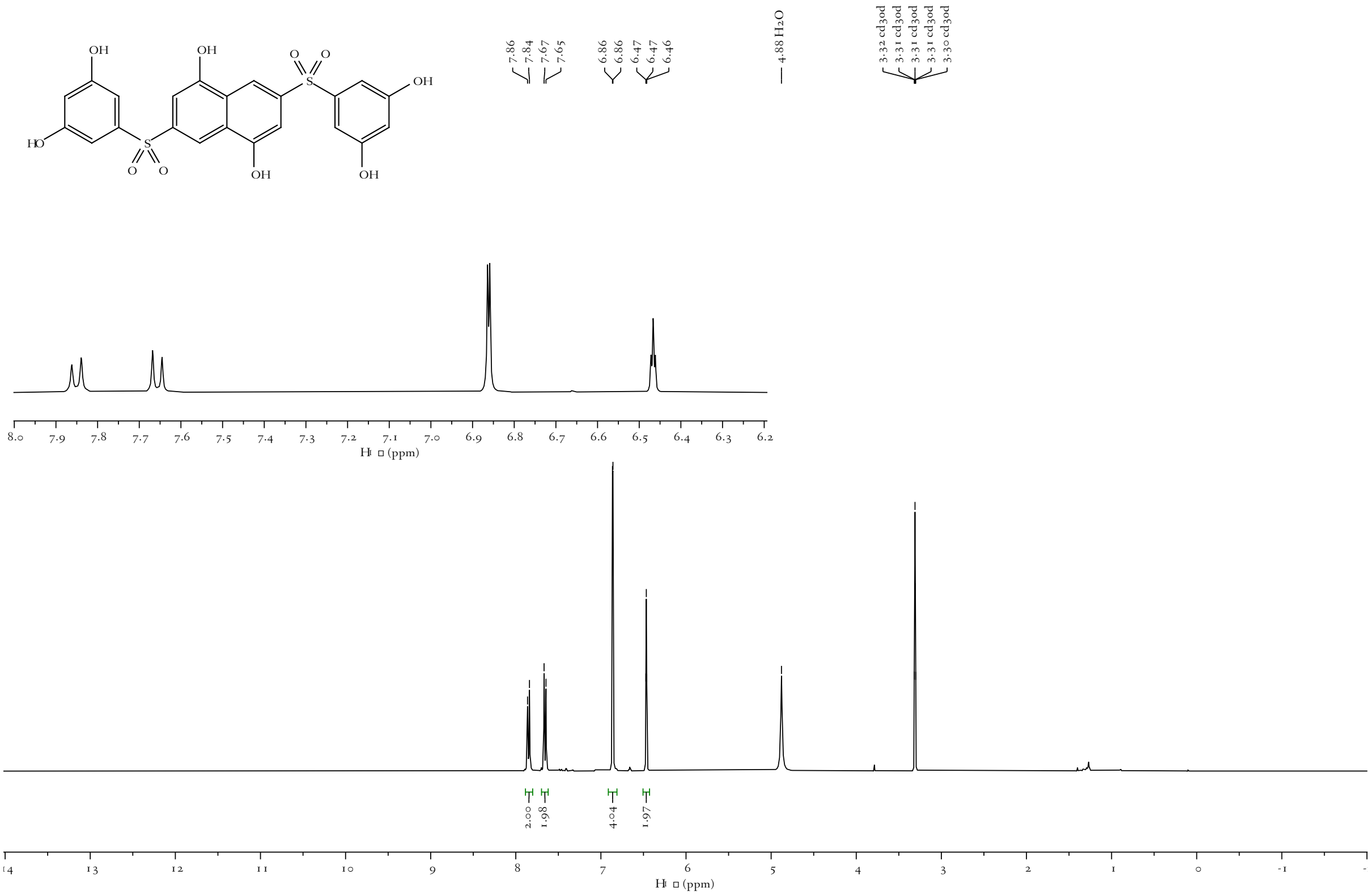

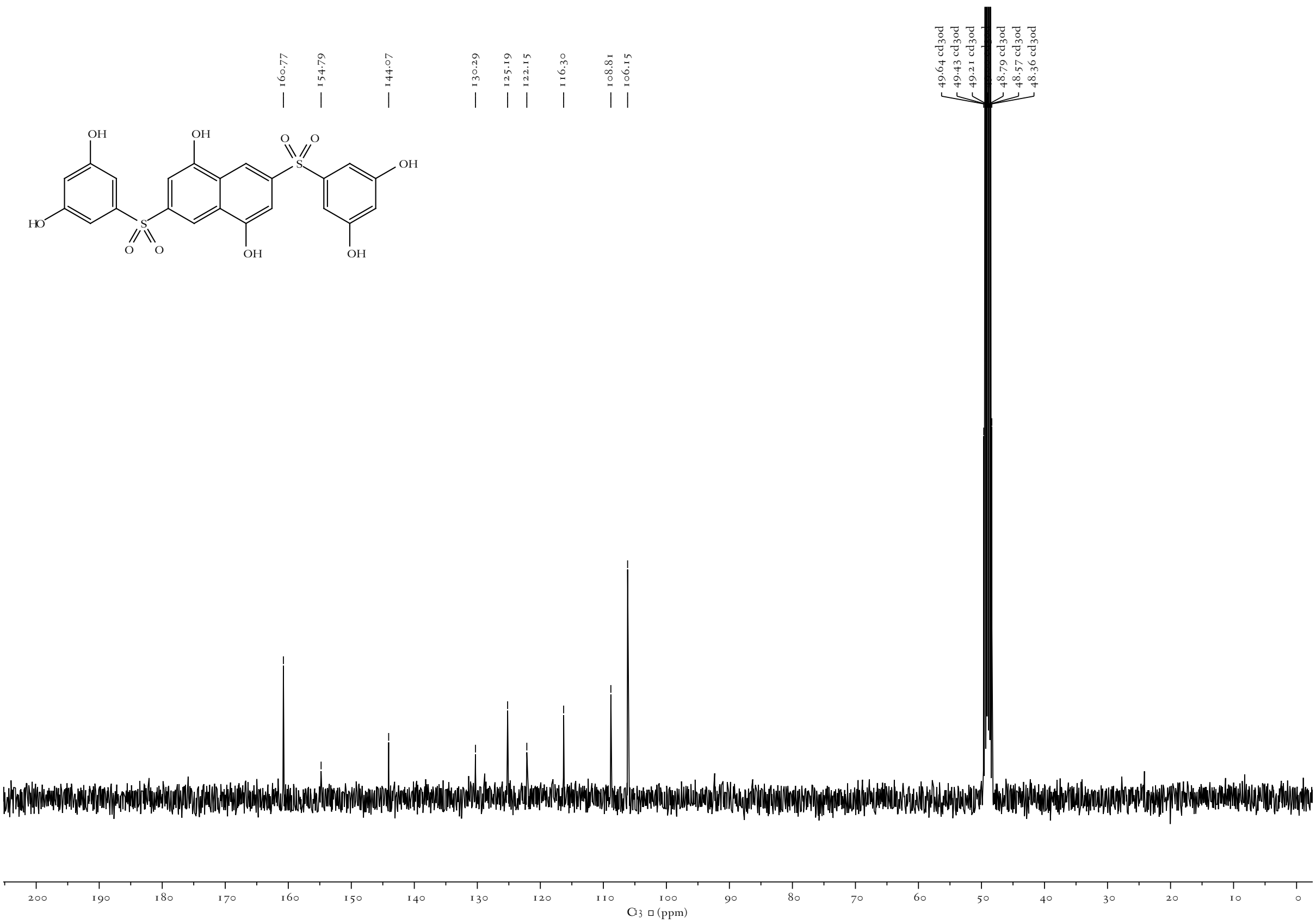

Abbreviations

AcOH/Acetic acid; DABCO/1,4-diazabicyclo[2.2.2]octane; DIPEA/Diisopropylethylamine; DMF/*N,N*-Dimethylformamide; DMSO/Dimethylsulfoxide; DPPF/1,1′-Bis(diphenylphosphino)ferrocene; EtOAc/Ethyl acetate; h/hour; HRMS; High-resolution mass spectroscopy; LC-MS/Liquid chromatography mass spectroscopy; m.p./Melting point; *m*CPBA/*meta*-Chlorobenzoic acid; min/minute(s); NaOAc/Sodium acetate; NMR/Nuclear magnetic resonance; r.t./Room temperature; *t*-BuLi/*tert*-Butyllithium; THF/Tetrahydrofuran.

References

[1] C. W. Cavanagh, M. H. Aukland, A. Hennessy, D. J. Procter, *Chem. Commun.* **2015**, *51*, 9272-9275, https://doi.org/10.1039/c5cc02676h.

[2] S. Kang, J. Huang, B. K. Lee, Y. S. Jung, E. Im, J. M. Koh, D. S. Im, *Biochim. Biophys. Acta. Mol. Cell. Biol. Lipids.* **2018**, *1863*, 105-116, https://doi.org/10.1016/j.bbalip.2017.11.002.

[3] O. Nilsson, A. P. Valaka, L. Haversen, A. Bogucka, I. Koteles, P. Brear, M. Rutberg, A. Gunnarsson, M. Hyvonen, M. Grotli, *Angew Chem Int Ed Engl* **2025**, *64*, e202513969, https://doi.org/10.1002/anie.202513969.

[4] J. Rivoal, C. R. Smith, T. F. Moraes, D. H. Turpin, W. C. Plaxton, *Anal. Biochem.* **2002**, *300*, 94-99, https://doi.org/10.1006/abio.2001.5445.

[5] A. Nain-Perez, O. Nilsson, A. Lulla, L. Haversen, P. Brear, S. Liljenberg, M. Hyvönen, J. Borén, M. Grøtli, *Eur. J. Med. Chem.* **2023**, *250*, 115-177, https://doi.org/10.1016/j.ejmech.2023.115177.
